# Validation of electronic health record-based International Society on Thrombosis and Haemostasis bleeding algorithms in a US health system

**DOI:** 10.1016/j.rpth.2026.106616

**Published:** 2026-04-30

**Authors:** Jaejin An, Alexander Hartenstein, Soon Kyu Choi, Bernadine Dizon, Hui Zhou, Xuan Huang, Susie Flores, Ming-Sum Lee, Brian Hocum, Kai Vogtländer, Khaled Abdelgawwad

**Affiliations:** 1 Department of Research & Evaluation, Kaiser Permanente Southern California, Pasadena, California, USA; 2 Department of Health Systems Science, Kaiser Permanente Bernard J. Tyson School of Medicine, Pasadena, California, USA; 3 Global Medical and Evidence, Bayer AG, Berlin, Germany; 4 Department of Cardiology, Kaiser Permanente Los Angeles Medical Center, Los Angeles, California, USA; 5 Division of Pharmaceuticals, Medical Affairs (HEOR), Bayer U.S. LLC, Whippany, New Jersey, USA; 6 Medical Affairs Statistics, Bayer AG, Wuppertal, Germany

**Keywords:** algorithms, electronic health records, hemorrhage, validation studies as topic, anticoagulants

## Abstract

**Background:**

Novel algorithms were developed to identify International Society on Thrombosis and Haemostasis (ISTH)-defined major and clinically relevant nonmajor (CRNM) bleeding events using electronic health record (EHR) data.

**Objectives:**

We applied and validated these EHR-based algorithms in a large US integrated health system.

**Methods:**

We conducted a retrospective cohort and chart review study of adults with atrial fibrillation and ≥1 oral anticoagulant (OAC) prescription between 2013 and 2021. EHR-based algorithms were applied to identify ISTH-defined major and CRNM bleeding from the first OAC fill until disenrollment, death, or June 2022, whichever occurred first. Through conditional sampling, 433 patients were selected for clinician chart review. Sensitivity, specificity, positive predictive values, and negative predictive values were calculated.

**Results:**

Among 62,106 adults (mean age, 72.6 years; 57.1% male), the ISTH major and CRNM bleeding event rates per the algorithms were 3.54 and 19.67 per 100 person-years, respectively, during a median follow-up of 4.3 years. Sensitivity and specificity were 0.91 (95% CI, 0.86, 0.94) and 0.92 (95% CI, 0.88, 0.95) for major bleeding, respectively, and 0.66 (95% CI, 0.54, 0.76) and 0.86 (95% CI, 0.82, 0.90) for CRNM bleeding, respectively. The positive and negative predictive values were 0.91 (95% CI, 0.87, 0.94) and 0.92 (95% CI, 0.89, 0.95) for major bleeding, respectively, and 0.52 (95% CI, 0.44, 0.60) and 0.92 (95% CI, 0.90, 0.94) for CRNM bleeding, respectively.

**Conclusion:**

The EHR-based ISTH major bleeding algorithm demonstrated high sensitivity and specificity, while the CRNM bleeding algorithm showed suboptimal sensitivity. The EHR-based ISTH algorithms may enhance understanding of OAC safety using real-world evidence.

## Essentials

• We tested how well electronic health record-based algorithms can identify bleeding cases.
• We compared algorithm-identified bleeding cases with chart review in a US healthcare system.
• The algorithms correctly identified most major bleeding cases but missed some nonmajor bleeding cases.
• Electronic health record-based algorithms could effectively identify bleeding cases.

## 1 Introduction

Patients with atrial fibrillation (AF) are recommended to take oral anticoagulants (OACs) such as vitamin K antagonists or direct OACs to prevent thromboembolic events [1,2]. However, bleeding is the main complication of OAC therapy, placing many patients with AF at high risk of bleeding [3]. Monitoring AF patients treated with OACs for bleeding events in clinical practice using electronic health records (EHRs) is critical. Yet, definitions of major bleeding vary widely, creating challenges for comparing bleeding rates across different settings and studies [4].

One of the most commonly used clinical definitions of bleeding events are the International Society on Thrombosis and Haemostasis (ISTH) definitions of major and clinically relevant nonmajor (CRNM) bleeding [4,5]. Among patients with AF receiving OAC treatment, bleeding rates have been estimated at approximately 2% to 4% per year for major bleeding and 4% to 12% per year for CRNM bleeding based on clinical trial data [[6], [7], [8], [9]]. Among AF patients not receiving OAC treatment, observational cohort studies reported rates of major bleeding ranging from 1.4% to 6% per year [10,11]. Furthermore, although the ISTH bleeding definitions are commonly used in clinical trial settings, they are seldom applied to observational studies using real-world data sources [12]. In the context of retrospective observational research using EHRs or administrative claims, it is necessary to accurately translate the concepts of ISTH major and CRNM bleeding into a real-world data environment.

Hartenstein et al. [12] developed novel algorithms to identify ISTH-defined major and CRNM bleeding events using a real-world data source: the Optum deidentified EHR. However, the performance of these algorithms has not been externally validated or assessed.

This study aimed to evaluate and validate the performance of these novel EHR-based algorithms for ISTH-defined major and CRNM bleeding in patients with AF in a large US integrated healthcare system.

## 2 Methods

### 2.1 Study setting

The Kaiser Permanente Southern California (KPSC) integrated healthcare delivery system provides comprehensive medical care services to more than 4.8 million members through 16 large, community-based hospitals and more than 200 medical offices throughout Southern California. All aspects of healthcare, including office visits, hospitalizations, pharmacy, laboratory services, and claims for healthcare utilization outside KPSC, are captured through clinical and administrative databases. The KPSC member population is socioeconomically diverse and broadly representative of the racial and ethnic groups living in Southern California [13]. The KPSC AF population is comparable to the general US AF population, but it includes a lower proportion of individuals who self-identify as White (65% vs 92%) [14,15].

### 2.2 Study design

We conducted a retrospective cohort and chart review study of AF patients with at least 1 prior OAC prescription, regardless of their treatment status at the time of the bleeding event, as baseline bleeding risk is clinically relevant when considering OAC initiation or reinitiation. The KPSC Institutional Review Board approved and granted a waiver of informed consent. The study followed the Strengthening the Reporting of Observational Studies in Epidemiology guidelines for observational research.

### 2.3 Study population

We included KPSC members aged ≥18 years with a confirmed AF diagnosis who had filled ≥1 OAC prescription (either warfarin or direct OAC) between January 1, 2013, and December 31, 2021 (Supplementary Figure). The index date was defined as the first date of OAC filling after AF diagnosis. A confirmed AF diagnosis was defined as ≥1 diagnosis (International Classification of Diseases [ICD], Ninth Revision, Clinical Modification [ICD-9-CM]: 427.31; and ICD, 10th Revision, Clinical Modification [ICD-10-CM]: I48.0, I48.1x, I48.2x, and I48.91) in hospital discharge records or emergency department visits in any position or ≥2 AF diagnoses in outpatient settings [16]. Members were excluded if they did not have an AF diagnosis 1 year prior to the index date, did not have 1 year of continuous membership (allowing a 45-day gap) prior to the index date, had any indication of pregnancy between January 1, 2013, and December 31, 2021, or had a diagnosis of any valvular heart disease (mechanical heart valve prosthesis or any heart valve pathology) 1 year prior to the index date. Members were followed from their index date to the end of KPSC membership, death, or the study end date (June 30, 2022), whichever occurred first.

### 2.4 EHR-based algorithms

**Figure 1:**
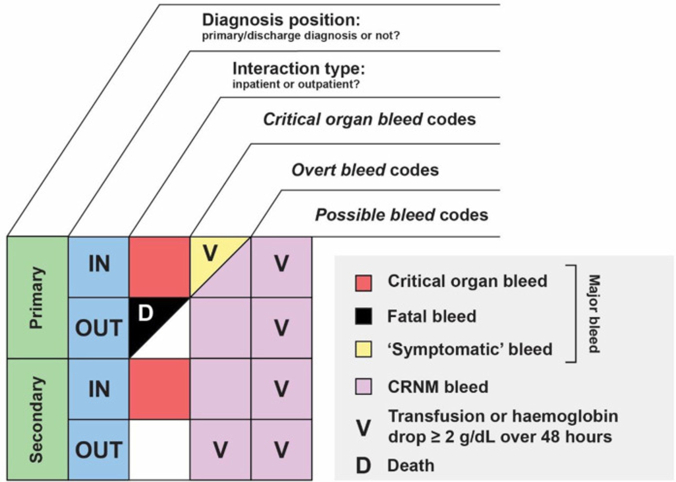
Bleeding algorithms for identifying bleeding events in real-world records [14]. “Primary” refers to a diagnosis code in the primary or discharge diagnosis position. CRNM, clinically relevant nonmajor; D, death; IN, inpatient interaction type; OUT, outpatient interaction type; V, transfusion or hemoglobin drop of ≥2 g/dL over 48 hours.

We applied the EHR-based algorithms developed by Hartenstein et al. [12] using ICD-9-CM and ICD-10-CM diagnosis codes, Current Procedural Terminology codes, laboratory data, and death data during the follow-up period to identify ISTH major bleeding (fatal bleeding, critical organ bleeding, and symptomatic bleeding) and CRNM bleeding (Figure 1 and Supplementary Table S1) [12]. Fatal bleeding was defined as the presence of a critical organ bleeding code in any position for an inpatient encounter or as the primary diagnosis in an outpatient setting, occurring within 45 days prior to death. A 45-day timeframe was selected because mortality data in the algorithm development dataset were available by month rather than the actual date of death [12]. Critical organ bleeding was defined as the presence of a critical organ bleeding diagnosis code in any position for an inpatient encounter. Symptomatic bleeding was defined as the presence of an overt bleeding diagnosis code (eg, gastrointestinal bleeding) in the principal position for an inpatient encounter, per bleed verification criteria, to identify severe bleeding events that could be categorized as major bleeding.

CRNM bleeding was defined as (1) the presence of overt bleeding diagnosis codes for an inpatient encounter, (2) overt bleeding diagnosis codes in the primary position in an outpatient setting, (3) overt bleeding diagnosis codes in the secondary position in an outpatient setting meeting the bleed verification criteria or (4) possible bleeding diagnosis codes in any position for an inpatient or outpatient setting meeting the bleed verification criteria. Bleed verification criteria were defined as (1) a drop in hemoglobin levels of ≥2 g/dL (1.24 mmol/L) within 48 hours of the encounter date compared with the most recent hemoglobin laboratory value within 6 months prior to the encounter, (2) a drop in hemoglobin levels of ≥2 g/dL (1.24 mmol/L) within 48 hours of another hemoglobin laboratory value, when ≥2 values are available after the encounter date, or (3) blood transfusion codes within 48 hours of the encounter date.

For encounters that met multiple bleeding algorithms, hierarchy was applied by bleeding type, in the order of fatal, critical organ, symptomatic, and CRNM bleeding. Algorithms were applied to the following in-person encounters: inpatient and emergency department encounters, office visits, and urgent care visits. Any encounters within ±1 day of another encounter with the same bleeding type were considered 1 bleeding event. Similarly, any outpatient encounter that led to an inpatient or emergency department encounter was considered part of an inpatient encounter per the algorithms.

### 2.5 Conditional sampling for chart review

Using conditional sampling, we selected 433 unique members, stratified by bleeding type, per EHR-based algorithms for manual chart review. We used conditional validation sampling, as it reduces the burden of random sampling, particularly when disease prevalence is expected to be low [17]. Previous studies have shown that the conditional sampling method yields better estimates than the unconditional simple random sampling method when the subsampling fraction falls within the recommended range [17].

We selected 200 ISTH major bleeding cases (50 cases as fatal bleeds, 50 cases as critical organ bleeds, and 100 cases as symptomatic bleeds), 100 CRNM bleeding cases, and 133 nonbleeding cases. Although a member may have multiple ISTH major bleeding events, only 1 randomly selected event per member was included in the chart review. Once a member was selected for chart review, they were removed from the subsequent selection pool. We selected 100 CRNM bleeding cases from those who were not selected for ISTH major bleeding for chart review. Finally, we selected 133 nonbleeding cases, comprising 34 borderline bleeding (with critical organ bleeding or overt bleeding diagnosis codes, but not meeting ISTH major or CRNM bleeding algorithm criteria), 33 borderline possible bleeding (with possible bleeding codes but not meeting CRNM bleeding algorithm criteria), and 66 expected cases without bleeding (no bleeding-related diagnosis codes).

### 2.6 Chart review

Chart adjudication was conducted by a trained research nurse (S.F.) who reviewed the 433 cases using the ISTH definitions to adjudicate major [4] and CRNM bleeding [5] (Supplementary Table S2). To standardize the chart review process and reduce bias and variability, the first 20 cases were independently reviewed by the research nurse (S.F.) and a study cardiologist (M.-S.L.). Data were entered into a chart review form using the electronic data capture tool Research Electronic Data Capture [18] and extracted for analysis. Once the double adjudication agreement level reached 90% for the initial cases, the research nurse independently reviewed the remaining cases. The study team was blinded to the bleeding results from the EHR-based algorithm. Unclear cases were reviewed by the study cardiologist and discussed with the study team.

For inpatient care or emergency department encounter cases, the chart adjudicator reviewed the entire episode of care up to the first postdischarge follow-up visit. For outpatient encounter cases, the adjudicator reviewed up to 7 days before and 7 days after the health encounter. Prespecified conditions to identify ISTH-defined major and CRNM bleeding (Supplementary Table S2), and additional questions (eg, patient’s chief complaint, whether the encounter addressed a new bleed or a historical bleed, bleed site, and whether the bleed was caused by trauma or an instrumented/medical procedure), were completed by the research nurse using a chart abstraction manual and form. For cases with multiple bleeding events, bleeding was adjudicated based on the most critical bleed event.

### 2.7 Demographic and clinical characteristics

We assessed sociodemographic characteristics, including age, sex (male and female), and self-reported race and ethnicity (Asian or Pacific Islander, non-Hispanic Black, Hispanic, non-Hispanic White, and other races), at the index date.

Clinical characteristics measured 12 months prior to the index date included hypertension, hyperlipidemia, diabetes, heart failure, myocardial infarction, ischemic and hemorrhagic stroke, transient ischemic attack (TIA), unstable angina, deep vein thrombosis, pulmonary embolism, systemic embolism, peripheral artery disease, anemia, cancer, chronic kidney disease, dementia, thrombocytopenia, alcohol use, the CHA_2_DS_2_-VASc (congestive heart failure, hypertension, age, diabetes mellitus, prior stroke or TIA or thromboembolism, vascular disease, age, sex category) score [19,20], HAS-BLED (hypertension, abnormal renal or liver function, stroke, bleeding history, labile international normalized ratio (INR), elderly age [>65 years], medication use predisposing to bleeding, alcohol use) score [21,22], and the Charlson Comorbidity Index [23], all defined using diagnostic codes, laboratory values, or medication use. We also assessed the history of ISTH major and CRNM bleeding using the EHR-based algorithms.

At the index date, we measured current smoking status (self-reported) and body mass index (<18.5, 18.5-24, and 25-29 kg/m^2^; class I or II obesity: 30-39 kg/m^2^; class III obesity: ≥40 kg/m^2^). Medication use of antihypertensives, antiplatelet agents (low-dose aspirin and clopidogrel), and nonsteroidal anti-inflammatory drugs in the 12 months prior to the index date was also identified using generic product identifier codes.

### 2.8 Statistical analysis

We used descriptive statistics (mean [SD] and counts [percentages]) to characterize the study cohort of eligible AF patients to whom the ISTH major and CRNM bleeding algorithms were applied. We also calculated the incidence of ISTH major and CRNM bleeding (event rate per 100 person-years) in the study cohort. The primary analytic dataset of the 433 members was used to evaluate the performance of the EHR-based algorithms. We evaluated measures of algorithm validity separately for ISTH major bleeding and CRNM bleeding. For each type of bleed, we calculated sensitivity, specificity, positive predictive value (PPV), and negative predictive value (NPV), considering true positives, false positives, true negatives, and false negatives in the sample population. For sensitivity and specificity, we computed 95% CIs using normal approximation [24]. For PPV and NPV, we computed 95% CIs using the formula by Mercaldo et al. [25]. All statistical analyses were performed using SAS Enterprise Guide (version 9.4; SAS Institute).

### 2.9 Sensitivity analyses

Several sensitivity analyses were conducted to assess which components of the EHR algorithms improved performance. For ISTH major bleeding, we assessed the performance after (1) removing all bleed verification requirements from the algorithms; (2) we removed bleed codes that were categorized as trauma per the algorithms [12] and reclassified cases that were adjudicated as ISTH major or CRNM bleeding due to trauma or an instrumented/medical procedure as nonbleeding events; (3) we reclassified overt bleeding codes in the inpatient setting and in *any position* (rather than the primary position) with bleed verification as ISTH major bleeding per the algorithms; (4) we reclassified overt bleeding codes per the algorithms in the inpatient setting and in any position with *or without* bleed verification (rather than requiring bleed verification) as ISTH major bleeding per the algorithms; (5) we reclassified all critical organ bleeding codes in the inpatient setting and in the *primary position only* (rather than allowing all positions) as ISTH major bleeding per the algorithms; (6) we reclassified critical organ bleeding codes in *any* setting and in any position (rather than an inpatient setting only) as ISTH major bleeding per the algorithms; and (7) required *death to occur during the encounter* (rather than within a 45-day window postencounter) for the fatal bleed definition per the chart review.

For sensitivity analyses defining CRNM bleeding, we also assessed performance after (1) removing all bleed verification requirements from the algorithms and after (2) removing bleed codes that were categorized as trauma. In addition, (3) we classified any overt bleeding diagnosis codes per the algorithms in the outpatient setting, secondary position, and *without* bleed verification (rather than requiring bleed verification) as CRNM bleeding; (4) reclassified any diagnosis codes that were classified as possible bleeds per the algorithms as nonbleeding diagnosis codes; (5) reclassified only anemia-related diagnosis codes under possible bleeding codes in the secondary position per the algorithms as nonbleeding; (6) reclassified overt bleeding codes in the inpatient setting and in *any position* (rather than the primary position) with bleed verification as ISTH major bleeding per the algorithms; and (7) reclassified overt bleeding codes in the inpatient setting and in any position with *or without* bleed verification (rather than requiring bleed verification) as ISTH major bleeding per the algorithms.

## 3 Results

**Figure 2:**
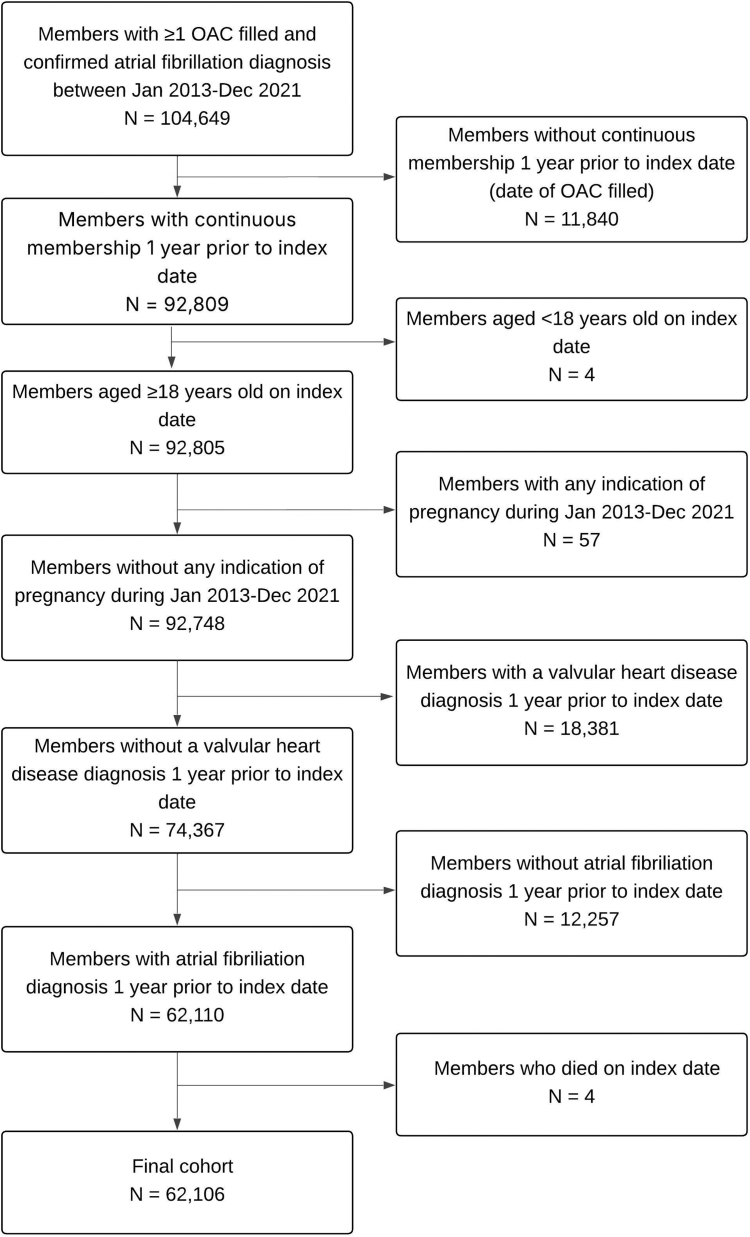
Study cohort. OAC, oral anticoagulant.

The study cohort included 62,106 adults with a confirmed AF diagnosis who had filled ≥1 OAC prescription (mean ± SD age, 72.6 ± 10.8 years; 57.1% male; 62.6% non-Hispanic White; Figure 2). Among the study cohort, 2.1% had a prior ISTH major bleeding event, and 14.2% had a prior CRNM bleeding event (Table 1). Additionally, 79.0% of the study cohort had hypertension, 81.6% hyperlipidemia, 22.4% heart failure, 13.3% myocardial infarction, 12.1% ischemic stroke, 11.9% pulmonary embolism, 34.4% anemia, 13.1% cancer, and 17.7% chronic kidney disease; 26.9% were treated with antiplatelet agents or nonsteroidal anti-inflammatory drugs. Demographic and clinical characteristics of the 433 adults in our primary analytic dataset for chart review were similar to those of the overall study cohort. The event rates for ISTH major and CRNM bleeding were 3.54 (95% CI, 3.47, 3.61) and 19.67 (95% CI, 19.51, 19.83) per 100 person-years, respectively, with a median follow-up of 4.3 years (Table 2).

**Table 1:** Cohort demographic and clinical characteristics.

Characteristics	Total cohort (*N* = 62,106)	Chart review sample (*n* = 433)
**Age** (y)		
Mean (SD)	72.6 ± 10.8	74.6 ± 9.3
Median (IQR)	74.0 (66.0, 80.0)	76.0 (70.0, 81.0)
**Sex**		
Male	35,472 (57.1)	237 (54.7)
Female	26,634 (42.9)	196 (45.3)
**Race and ethnicity**		
Non-Hispanic White	38,851 (62.6)	271 (62.6)
Non-Hispanic Black	4835 (7.8)	37 (8.5)
Hispanic	12,518 (20.2)	82 (18.9)
Asian/Pacific Islander	5291 (8.5)	41 (9.5)
Other	611 (1.0)	2 (0.5)
**BMI (kg/m^2^)**	30.0 ± 7.2	29.7 ± 6.6
**BMI categories (kg/m^2^)**		
<18.5	821 (1.3)	8 (1.9)
18.5 to <25	14,180 (23.0)	82 (19.0)
25 to <30	20,510 (33.2)	176 (40.7)
30-40, class I-II obesity	20,797 (33.7)	136 (31.5)
>40, class III obesity	5393 (8.7)	30 (6.9)
Missing, *n*	405	1
**Health behaviors**		
Alcohol use	2699 (4.3)	22 (5.1)
Current smoker	2824 (4.5)	23 (5.3)
**History of bleeding**		
ISTH major bleeding	1305 (2.1)	6 (1.4)
CRNM bleeding	8820 (14.2)	83 (19.2)
**Cardiometabolic disease**		
Hypertension	49,058 (79.0)	368 (85.0)
Hyperlipidemia	50,694 (81.6)	371 (85.7)
Diabetes	21,972 (35.4)	166 (38.3)
Heart failure	13,941 (22.4)	103 (23.8)
Myocardial infarction	8230 (13.3)	54 (12.5)
Ischemic stroke	7492 (12.1)	64 (14.8)
Hemorrhagic stroke	394 (0.6)	2 (0.5)
Transient ischemic attack	1526 (2.5)	12 (2.8)
Unstable angina	575 (0.9)	4 (0.9)
Deep vein thrombosis	2009 (3.2)	8 (1.8)
Pulmonary embolism	7372 (11.9)	61 (14.1)
Systemic embolism	1217 (2.0)	7 (1.6)
Peripheral artery disease	899 (1.4)	13 (3.0)
**Other high-risk bleeding conditions**		
Anemia	21,359 (34.4)	174 (40.2)
Cancer	8153 (13.1)	59 (13.6)
Chronic kidney disease	10,989 (17.7)	94 (21.7)
Dementia	1864 (3.0)	10 (2.3)
Thrombocytopenia	2979 (4.8)	24 (5.5)
**Stroke/bleed risk scores**		
CHA_2_DS_2_-VASc score	3.3 ± 1.5	3.6 ± 1.5
HAS-BLED score	2.3 ± 1.1	2.5 ± 1.1
**Charlson Comorbidity Index**	2.2 ± 1.7	2.5 ± 1.7
**Medication use**		
Antihypertensives	58,255 (93.8)	413 (95.4)
Antiplatelet agents or nonsteroidal anti-inflammatory drugs	16,715 (26.9)	115 (26.6)

Data are presented as *n* (%), mean ± SD, or median (IQR). CHA_2_DS_2_-VASc: range, 0 to 8; HAS-BLED: range, 0 to 7; Charlson Comorbidity Index: range, 0 to 10.

BMI, body mass index; CHA_2_DS_2_-VASc, congestive heart failure, hypertension, age, diabetes mellitus, prior stroke or TIA or thromboembolism, vascular disease, age, sex category; CRNM, clinically relevant nonmajor; HAS-BLED, hypertension, abnormal renal or liver function, stroke, bleeding history, labile international normalized ratio (INR), elderly age [>65 years], medication use predisposing to bleeding, alcohol use; ISTH, International Society on Thrombosis and Haemostasis.

**Table 2:** International Society on Thrombosis and Haemostasis major and clinically relevant nonmajor bleeding event rates during follow-up.

ISTH major and CRNM bleeding	Event rates per 100 person-years (95% CI)
ISTH major bleeding	3.54 (3.47, 3.61)
Fatal bleeding	0.50 (0.48, 0.53)
Critical organ bleeding	1.36 (1.32, 1.40)
Symptomatic bleeding	1.68 (1.63, 1.72)
CRNM bleeding	19.67 (19.51, 19.83)

CRNM, clinically relevant nonmajor; ISTH, International Society on Thrombosis and Haemostasis.

The primary analysis results for the EHR-based ISTH major bleeding algorithms showed a sensitivity of 0.91 (95% CI, 0.86, 0.94), a specificity of 0.92 (95% CI, 0.88, 0.95), a PPV of 0.91 (95% CI, 0.87, 0.94), and an NPV of 0.92 (95% CI, 0.89, 0.95; Table 3). Performance indices were also calculated for bleeding categories within the ISTH major bleeding definition, per the EHR-based algorithms (Supplementary Table S3). After removing bleed verification from all algorithms, sensitivity remained unchanged, but specificity decreased to 0.82 (95% CI, 0.76, 0.87) and PPV decreased to 0.81 (95% CI, 0.77, 0.85) for ISTH major bleeding algorithms (Table 3). Removal of trauma-related bleeding codes, per the EHR-based algorithms and chart review, resulted in decreased sensitivity, specificity, and PPV, but a minimal increase in NPV (0.02) compared with the primary analysis assessing ISTH major bleeding performance.

**Table 3:** Performance of the International Society on Thrombosis and Haemostasis major bleeding algorithms.

ISTH major bleeding	Sensitivity estimate (95% CI)	Specificity estimate (95% CI)	PPV estimate (95% CI)	NPV estimate (95% CI)
**Primary analysis**	0.91 (0.86, 0.94)	0.92 (0.88, 0.95)	0.91 (0.87, 0.94)	0.92 (0.89, 0.95)
**Sensitivity analysis**				
(1) Removal of all bleed verification^a^ requirements per the algorithms	0.91 (0.86, 0.95)	0.82 (0.76, 0.87)	0.81 (0.77, 0.85)	0.91 (0.88, 0.95)
(2) Removal of trauma bleeding diagnosis codes per the algorithms and chart review	0.88 (0.81, 0.93)	0.88 (0.83, 0.91)	0.76 (0.71, 0.82)	0.94 (0.92, 0.97)
(3) Overt bleeding codes in the inpatient setting and in *any position* (rather than the primary position) with bleed verification	0.95 (0.90, 0.97)	0.91 (0.87, 0.94)	0.90 (0.86, 0.94)	0.95 (0.92, 0.98)
(4) Overt bleeding codes in the inpatient setting and in any position with *or without* bleed verification (rather than requiring bleed verification)	0.95 (0.91, 0.98)	0.73 (0.66, 0.78)	0.75 (0.71, 0.79)	0.94 (0.91, 0.98)

ISTH, International Society on Thrombosis and Haemostasis; NPV, negative predictive value; PPV, positive predictive value.

a Bleed verification per electronic health record-based algorithms is defined as a (1) drop in hemoglobin levels of ≥2 g/dL (1.24 mmol/L) within 48 hours of an encounter, compared with the closest value within 6 months prior; (2) a drop in hemoglobin levels of ≥2 g/dL (1.24 mmol/L) within 48 hours during an encounter; or (3) blood transfusion codes within 48 hours of an inpatient encounter or within 48 hours of an outpatient encounter.

When we modified the overt bleeding code algorithm to allow any position (rather than the primary position) with bleed verification, sensitivity increased from 0.91 (95% CI, 0.86, 0.94) to 0.95 (95% CI, 0.90, 0.97); there were minimal decreases (0.01) in specificity and PPV compared with the primary analysis results. There were minimal changes in performance indices after changing the settings or positions of critical organ bleeding code algorithms (Supplementary Table S4). When we defined fatal bleeding, a category within ISTH major bleeding, as death occurring during an encounter rather than within 45 days of admission, sensitivity, specificity, and PPV decreased, though there was no change in performance indices for overall ISTH major bleeding compared with the primary analysis.

The primary analysis results for the EHR-based CRNM bleeding algorithms showed a sensitivity of 0.66 (95% CI, 0.54, 0.76), a specificity of 0.86 (95% CI, 0.82, 0.90), a PPV of 0.52 (95% CI, 0.44, 0.60), and an NPV of 0.92 (95% CI, 0.90, 0.94; Table 4). Removing the bleed verification requirement from CRNM bleeding decreased the sensitivity to 0.63 (95% CI, 0.52, 0.74), specificity to 0.75 (95% CI, 0.70, 0.80), and PPV to 0.36 (95% CI, 0.31, 0.42). Removing trauma-related bleeding codes per the algorithms and chart review also resulted in decreased sensitivity, specificity, and PPV, but increased NPV compared with the primary analysis results. When we no longer required bleed verification for overt bleeding codes in the outpatient setting and in the secondary position, sensitivity increased to 0.82 (95% CI, 0.72, 0.90), with minimal changes in specificity, PPV, and NPV. After reclassifying all possible bleed codes as no-bleed codes for CRNM bleeding, specificity increased to 0.91 (95% CI, 0.87, 0.94), and PPV increased to 0.62 (95% CI, 0.53, 0.71), with the same sensitivity and NPV. Reclassifying only anemia-related codes (within possible bleed codes) in the secondary position to no-bleed codes yielded similar results (Supplementary Table S5).

**Table 4:** Performance of the clinically relevant nonmajor bleeding algorithms.

CRNM bleeding	Sensitivity estimate (95% CI)	Specificity estimate (95% CI)	PPV estimate (95% CI)	NPV estimate (95% CI)
**Primary analysis**	0.66 (0.54, 0.76)	0.86 (0.82, 0.90)	0.52 (0.44, 0.60)	0.92 (0.90, 0.94)
**Sensitivity analysis**				
(1) Removal of all bleed verification^a^ requirements per the algorithms	0.63 (0.52, 0.74)	0.75 (0.70, 0.80)	0.36 (0.31, 0.42)	0.90 (0.88, 0.93)
(2) Removal of trauma bleeding diagnosis codes per the algorithms and chart review	0.63 (0.48, 0.77)	0.85 (0.81, 0.89)	0.35 (0.28, 0.43)	0.95 (0.93, 0.97)
(3) Overt bleeding codes in the outpatient setting and in the secondary position *without* bleed verification (rather than requiring bleed verification)	0.82 (0.72, 0.90)	0.82 (0.78, 0.86)	0.50 (0.44, 0.56)	0.95 (0.93, 0.97)
(4) Reclassifying possible bleeding codes as no bleeds per the algorithms	0.66 (0.54, 0.76)	0.91 (0.87, 0.94)	0.62 (0.53, 0.71)	0.92 (0.90, 0.94)

CRNM, clinically relevant nonmajor; NPV, negative predictive value; PPV, positive predictive value.

a Bleed verification per electronic health record-based algorithms is defined as a (1) drop in hemoglobin levels of ≥2 g/dL (1.24 mmol/L) within 48 hours of an encounter, compared with the closest value within 6 months prior; (2) a drop in hemoglobin levels of ≥2 g/dL (1.24 mmol/L) within 48 hours during an encounter; or (3) blood transfusion codes within 48 hours of an inpatient encounter or within 48 hours of an outpatient encounter.

**Figure 3:**
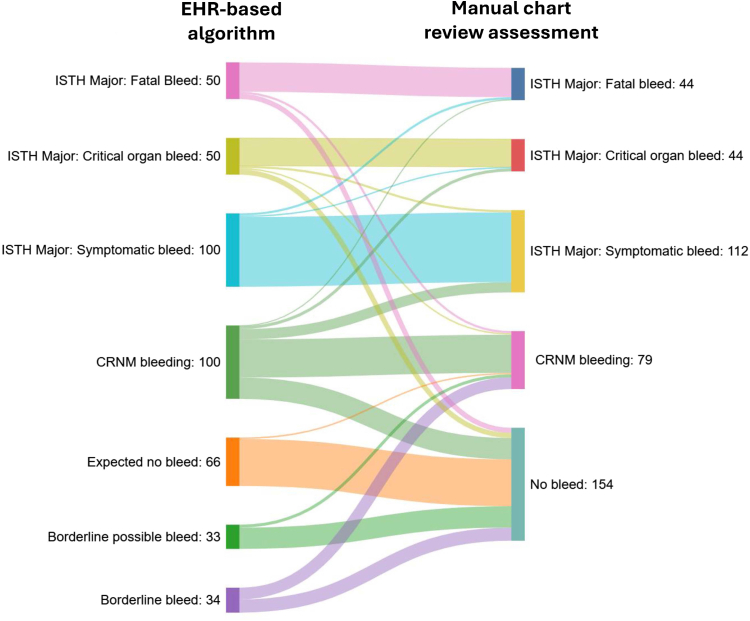
Sankey diagram of the electronic health record (EHR)-based algorithms and chart review results. CRNM, clinically relevant nonmajor; ISTH, International Society on Thrombosis and Haemostasis.

The Sankey diagram of the primary analysis results visually illustrates the validation of the EHR-based algorithms through chart adjudication (Figure 3). The majority of cases identified as ISTH major bleeding per the algorithms were also identified as ISTH major bleeding per chart adjudication. Fourteen ISTH major bleeding cases (7 fatal bleeds and 7 critical organ bleeds) identified by the EHR-based algorithms were adjudicated as nonbleeding cases by chart review: no evidence of bleeding was identified in the medical charts, nor did the patient have a history of bleeding, and the diagnosis codes were carried forward. Results for CRNM bleeding per the algorithms were more fragmented, with 19 cases identified as ISTH major bleeding and 29 as nonbleeding events per chart adjudication. Of the 29 nonbleeding cases, patients had a history of bleeding with a bleeding diagnosis code carried forward, an anemia-related diagnosis code with no evidence of bleeding in the medical charts, or a minor bleeding event with a bleeding diagnosis in the secondary position. Among the nonbleeding cases identified by the algorithms as CRNM bleeding per chart adjudication, all met the CRNM bleeding criterion of bleeding prompting a face-to-face clinic evaluation; 4 cases also met other CRNM bleeding criteria.

## 4 Discussion

The current study demonstrated that the ISTH major bleeding algorithms performed well (sensitivity, specificity, PPV, and NPV all >0.90), suggesting that these EHR-based algorithms are a reliable method for identifying ISTH major bleeding using secondary databases. The sensitivity analyses suggest that the ISTH major bleeding algorithms can still be applied reliably without bleed verification criteria; thus, these algorithms are expected to perform well in databases that lack the laboratory values required for bleed verification, such as claims data. The sensitivity of the CRNM bleeding algorithm was suboptimal, though performance increased significantly after modifying its components, underscoring the importance of understanding each component.

While ISTH major and CRNM bleeding are commonly used in clinical trials, their application to secondary databases has been limited. The novel algorithms developed by Hartenstein et al. [12] use ICD-9-CM and ICD-10-CM bleeding diagnosis codes, hemoglobin values, blood transfusion procedural codes, and death data, as well as ICD diagnosis code settings (inpatient or outpatient) and positions (primary or secondary). ICD diagnosis codes categorized under critical organ bleeding codes and overt bleeding codes [12] were key components of the ISTH major bleeding algorithm performance. The ISTH major bleeding algorithm required critical organ bleeding codes to be in the inpatient setting and in the primary position. However, sensitivity analyses that modified the position and setting of critical organ bleeding codes had either no effect or only a minimal decrease in specificity and PPV, indicating that the diagnosis codes categorized as critical organ bleeding correctly captured the severity of major bleeding and the hospitalization for such events. Overt bleeding codes in the inpatient setting, with bleed verification, also identified ISTH major bleeding reasonably well. In our sensitivity analyses, allowing overt bleeding codes in any position while requiring bleed verification increased sensitivity to 0.95, with minimal decrease in specificity and PPV.

Algorithm development and validation studies identifying major bleeding using EHR data are varied and limited. The definition of major bleeding was inconsistent across studies, though many included the same algorithm components as those used by Hartenstein et al. [12,[26], [27], [28], [29]]. These studies are not limited to an AF population treated with OACs. Moreover, validation studies reporting the sensitivity of these algorithms are limited by the need for larger sample sizes, including cases without bleeding. However, our study results align with other algorithm validation studies of major bleeding, though these algorithms do not conceptually align with the ISTH major bleeding definition. Cunningham et al. [29] developed and validated an algorithm for “bleeding related hospitalization” and reported PPVs ranging from 0.88 to 0.99 by bleeding site, using validated major bleeding algorithms, in a sample of 186 patients. Pasea et al. [26] reviewed 283 cases and reported a sensitivity of 0.71 (95% CI, 0.48, 0.89), a specificity of 0.99 (95% CI, 0.97, 1.00), and a PPV of 0.88 (95% CI, 0.64, 0.99) for validated major bleeding algorithms. Friberg and Skeppholm [30] validated bleeding algorithms on an AF population of 761 patients in Sweden, reporting a sensitivity of 0.85 (95% CI, 0.80, 0.88), a specificity of 0.96 (95% CI, 0.94, 1.00), and a PPV of 0.93 (95% CI, 0.89, 0.96) for overall major bleeding events.

In some cases, EHR-based bleeding algorithms relied solely on ICD codes, without bleed verification, blood transfusion data, or requirements for diagnosis, position, or setting. In these studies, the sensitivity and specificity of major bleeding algorithms ranged from 0.16 to 0.65 and from 0.99 to 1.00, respectively [27,28], with one study showing improvements in both after bleed verification data were included [27]. In our validation of the Hartenstein et al. [12] algorithms, removal of bleed verification still resulted in strong performance in sensitivity (0.91), specificity (0.82), and PPV (0.81) for ISTH major bleeding, indicating that this EHR-based algorithm can be optimized and applied to secondary datasets that may not have access to hemoglobin laboratory values or blood transfusion data.

While algorithm performance for ISTH major bleeding was strong, the results for CRNM bleeding were suboptimal, with a sensitivity of 0.66 and a PPV of 0.52. Although the ISTH has set recommendations for defining CRNM bleeding [5], few studies have assessed the performance of CRNM bleeding algorithms. One previous study assessed the performance of the CRNM bleeding algorithm, reporting a sensitivity of 0.50 to 0.56 and a PPV of 0.50 [27]. While our study findings are similar to those of the previous study [27], the sensitivity analysis improved the CRNM bleeding algorithm’s sensitivity to 0.82 when overt bleeding codes were used in the outpatient setting, and the bleed verification requirement was removed. Additionally, removing all possible bleed codes also improved the CRNM bleeding algorithm’s specificity to 0.91, as CRNM bleeding identified with possible bleed codes, particularly anemia-related codes, was mainly false-positive cases. These findings suggest how best to optimize these algorithms for CRNM bleeding. Nonetheless, it is important to recognize that some false-positive CRNM bleeding cases were attributed to a history of bleeding carried forward in the EHR. Future studies could explore methods to better distinguish between a history of bleeding and active bleeding using various components of the EHR.

The validated EHR-based algorithms included trauma-related bleeding codes [12]. The inclusion of trauma bleeding codes improved the performance of ISTH major and CRNM bleeding, whereas removing the codes reduced performance (sensitivity = 0.88 and PPV = 0.76). Cunningham et al. [29] performed explicit trauma removal procedures, identifying patients who experienced trauma and removing them, even if bleeding occurred. Most algorithm validation studies do not specify how, or whether, trauma-related bleeding codes were included in their algorithms [26,27]. ISTH major and CRNM bleeding definitions also do not explicitly provide guidance on how trauma-related bleeding should be considered [5]. Yet, our results indicate that trauma-related bleeding codes may be important to include, especially in the context of OAC usage, which may greatly exacerbate trauma-related bleeding. Explicit guidance that reflects real-world scenarios, including trauma-related bleeding, would improve the application of ISTH major bleeding algorithms.

This study has several limitations. First, the EHR-based algorithms defined fatal death as death occurring within 45 days postencounter, based on the availability of death data in the algorithm development dataset (Optum); we applied the same logic to our chart review adjudication to match the algorithm. However, allowing a 45-day time window may result in increased false positives for fatal bleeds, and we could not confirm those cases. Second, the algorithms used procedural codes to identify blood transfusions, but details on the number of units transfused and the timing of transfusion(s) were not captured. For encounters that occurred outside KPSC, we were unable to apply the hemoglobin drop bleed verification requirements per the algorithms because laboratory values were not consistently available. Third, the performance metrics were calculated directly from the adjudicated 2 × 2 tables, without applying weights to account for conditional sampling. These estimates may not fully reflect the algorithms’ performance across the overall population. Finally, our validation study was conducted only at a single integrated health system; thus, performance in other health systems or datasets remains unclear. However, sensitivity analyses showed that algorithm performance remained high without bleed verification data and could be further optimized by modifying its components, indicating that the algorithms may still perform well across different healthcare settings in the US.

## 5 Conclusion

The EHR-based ISTH major bleeding algorithms demonstrated strong sensitivity and specificity, whereas the sensitivity and PPV of the CRNM bleeding algorithms were suboptimal. These findings suggest that EHR-based ISTH algorithms may enhance the understanding of OAC safety using real-world evidence.
