# Supplementary material for: Validation of electronic health record-based International Society on Thrombosis and Haemostasis bleeding algorithms in a US health system
Source: Res Pract Thromb Haemost. 2026 Apr 30;10(4):106616. doi: 10.1016/j.rpth.2026.106616 (PMC13224354; doi:10.1016/j.rpth.2026.106616)
Supplement: Supplementary Figure S1 and Tables S1-S5 [file mmc1.docx]

**Validation of electronic health record-based International Society of Thrombosis and Haemostasis bleeding algorithms in a US health system**

Short title: Validation of ISTH bleeding algorithms

Jaejin An, PhD,^1,2^ Alexander Hartenstein, MD ^3^ Soon Kyu Choi, MSc,^1^ Bernadine Dizon, MPH,^1^ Hui Zhou, PhD,^1,2^ Xuan Huang, MS,^1^ Susie Flores, BSN,^1^ Ming-Sum Lee, MD, PhD,^2,4^ Brian Hocum, PharmD, MS,^5^ Kai Vogtländer, MSc,^6^ Khaled Abdelgawwad, MSc^3^

^1^ Department of Research & Evaluation, Kaiser Permanente Southern California, Pasadena, CA

^2^ Department of Health Systems Science, Kaiser Permanente Bernard J. Tyson School of Medicine, Pasadena, CA

^3^ Global Medical and Evidence, Bayer AG, Berlin, Germany

^4^ Department of Cardiology, Kaiser Permanente Los Angeles Medical Center, Los Angeles, CA

^5^ Pharmaceuticals Division, Medical Affairs (HEOR), Bayer U.S. LLC, Whippany, NJ, USA

^6^ Medical Affairs Statistics, Bayer AG, Wuppertal, Germany

Address for correspondence: Jaejin An, BPharm, Ph.D. Department of Research & Evaluation, Kaiser Permanente Southern California. 100 S Los Robles, 2nd Floor, Pasadena, CA 91101. Fax: (626) 564-3409. Phone: (626) 564-5906. Email: jaejin.x.an@kp.org.

**Supplementary Figure. Study Design Schema**

**
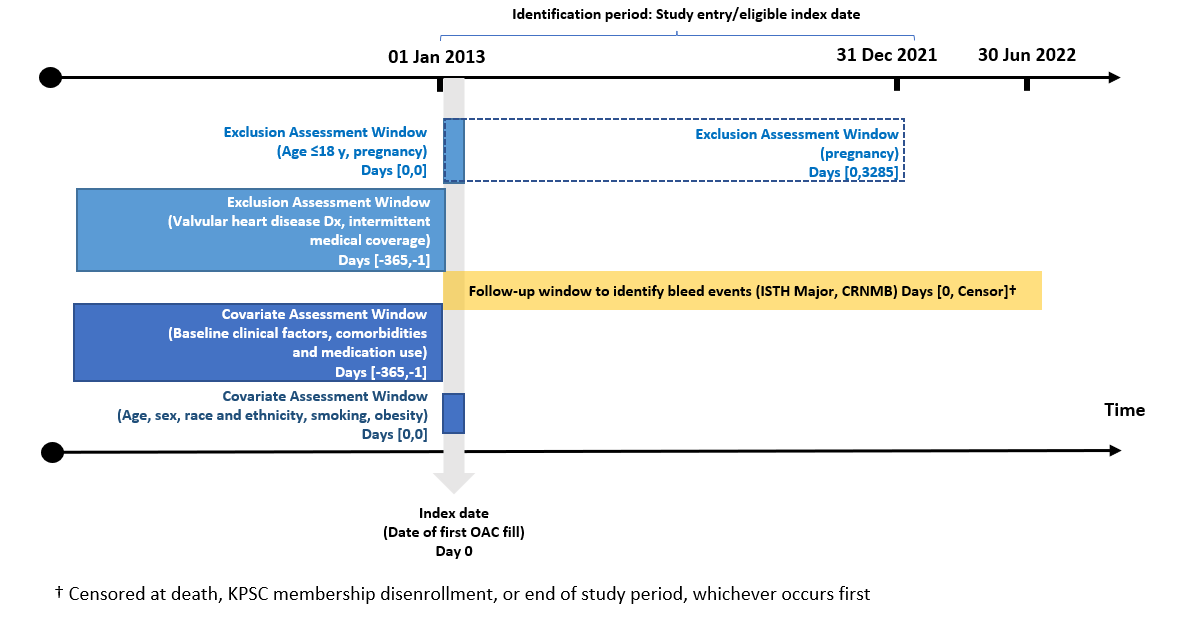
**

**Supplementary Table S1. Bleeding Identification Diagnosis and Procedural Codes for Algorithms**

Supplementary Table S1a. Critical organ bleed disease codes, ICD-9-CM and ICD-10-CM

| **Code** | **Code Type** | **Description** | **Trauma Code Flag** |
| --- | --- | --- | --- |
| 800.20 | ICD9CM | Closed fracture of vault of skull with subarachnoid, subdural, and extradural hemorrhage, unspecified state of consciousness | 1 |
| 800.21 | ICD9CM | Closed fracture of vault of skull with subarachnoid, subdural, and extradural hemorrhage, with no loss of consciousness | 1 |
| 800.22 | ICD9CM | Closed fracture of vault of skull with subarachnoid, subdural, and extradural hemorrhage, with brief [less than one hour] loss of consciousness | 1 |
| 800.23 | ICD9CM | Closed fracture of vault of skull with subarachnoid, subdural, and extradural hemorrhage, with moderate [1-24 hours] loss of consciousness | 1 |
| 800.24 | ICD9CM | Closed fracture of vault of skull with subarachnoid, subdural, and extradural hemorrhage, with prolonged [more than 24 hours] loss of consciousness and return to pre-existing conscious level | 1 |
| 800.25 | ICD9CM | Closed fracture of vault of skull with subarachnoid, subdural, and extradural hemorrhage, with prolonged [more than 24 hours] loss of consciousness, without return to pre-existing conscious level | 1 |
| 800.26 | ICD9CM | Closed fracture of vault of skull with subarachnoid, subdural, and extradural hemorrhage, with loss of consciousness of unspecified duration | 1 |
| 800.29 | ICD9CM | Closed fracture of vault of skull with subarachnoid, subdural, and extradural hemorrhage, with concussion, unspecified | 1 |
| 800.30 | ICD9CM | Closed fracture of vault of skull with other and unspecified intracranial hemorrhage, unspecified state of consciousness | 1 |
| 800.31 | ICD9CM | Closed fracture of vault of skull with other and unspecified intracranial hemorrhage, with no loss of consciousness | 1 |
| 800.32 | ICD9CM | Closed fracture of vault of skull with other and unspecified intracranial hemorrhage, with brief [less than one hour] loss of consciousness | 1 |
| 800.33 | ICD9CM | Closed fracture of vault of skull with other and unspecified intracranial hemorrhage, with moderate [1-24 hours] loss of consciousness | 1 |
| 800.34 | ICD9CM | Closed fracture of vault of skull with other and unspecified intracranial hemorrhage, with prolonged [more than 24 hours] loss of consciousness and return to pre-existing conscious level | 1 |
| 800.35 | ICD9CM | Closed fracture of vault of skull with other and unspecified intracranial hemorrhage, with prolonged [more than 24 hours] loss of consciousness, without return to pre-existing conscious level | 1 |
| 800.36 | ICD9CM | Closed fracture of vault of skull with other and unspecified intracranial hemorrhage, with loss of consciousness of unspecified duration | 1 |
| 800.39 | ICD9CM | Closed fracture of vault of skull with other and unspecified intracranial hemorrhage, with concussion, unspecified | 1 |
| 800.70 | ICD9CM | Open fracture of vault of skull with subarachnoid, subdural, and extradural hemorrhage, unspecified state of consciousness | 1 |
| 800.71 | ICD9CM | Open fracture of vault of skull with subarachnoid, subdural, and extradural hemorrhage, with no loss of consciousness | 1 |
| 800.72 | ICD9CM | Open fracture of vault of skull with subarachnoid, subdural, and extradural hemorrhage, with brief [less than one hour] loss of consciousness | 1 |
| 800.73 | ICD9CM | Open fracture of vault of skull with subarachnoid, subdural, and extradural hemorrhage, with moderate [1-24 hours] loss of consciousness | 1 |
| 800.74 | ICD9CM | Open fracture of vault of skull with subarachnoid, subdural, and extradural hemorrhage, with prolonged [more than 24 hours] loss of consciousness and return to pre-existing conscious level | 1 |
| 800.75 | ICD9CM | Open fracture of vault of skull with subarachnoid, subdural, and extradural hemorrhage, with prolonged [more than 24 hours] loss of consciousness, without return to pre-existing conscious level | 1 |
| 800.76 | ICD9CM | Open fracture of vault of skull with subarachnoid, subdural, and extradural hemorrhage, with loss of consciousness of unspecified duration | 1 |
| 800.79 | ICD9CM | Open fracture of vault of skull with subarachnoid, subdural, and extradural hemorrhage, with concussion, unspecified | 1 |
| 800.80 | ICD9CM | Open fracture of vault of skull with other and unspecified intracranial hemorrhage, unspecified state of consciousness | 1 |
| 800.81 | ICD9CM | Open fracture of vault of skull with other and unspecified intracranial hemorrhage, with no loss of consciousness | 1 |
| 800.82 | ICD9CM | Open fracture of vault of skull with other and unspecified intracranial hemorrhage, with brief [less than one hour] loss of consciousness | 1 |
| 800.83 | ICD9CM | Open fracture of vault of skull with other and unspecified intracranial hemorrhage, with moderate [1-24 hours] loss of consciousness | 1 |
| 800.84 | ICD9CM | Open fracture of vault of skull with other and unspecified intracranial hemorrhage, with prolonged [more than 24 hours] loss of consciousness and return to pre-existing conscious level | 1 |
| 800.85 | ICD9CM | Open fracture of vault of skull with other and unspecified intracranial hemorrhage, with prolonged [more than 24 hours] loss of consciousness, without return to pre-existing conscious level | 1 |
| 800.86 | ICD9CM | Open fracture of vault of skull with other and unspecified intracranial hemorrhage, with loss of consciousness of unspecified duration | 1 |
| 800.89 | ICD9CM | Open fracture of vault of skull with other and unspecified intracranial hemorrhage, with concussion, unspecified | 1 |
| 801.20 | ICD9CM | Closed fracture of base of skull with subarachnoid, subdural, and extradural hemorrhage, unspecified state of consciousness | 1 |
| 801.21 | ICD9CM | Closed fracture of base of skull with subarachnoid, subdural, and extradural hemorrhage, with no loss of consciousness | 1 |
| 801.22 | ICD9CM | Closed fracture of base of skull with subarachnoid, subdural, and extradural hemorrhage, with brief [less than one hour] loss of consciousness | 1 |
| 801.23 | ICD9CM | Closed fracture of base of skull with subarachnoid, subdural, and extradural hemorrhage, with moderate [1-24 hours] loss of consciousness | 1 |
| 801.24 | ICD9CM | Closed fracture of base of skull with subarachnoid, subdural, and extradural hemorrhage, with prolonged [more than 24 hours] loss of consciousness and return to pre-existing conscious level | 1 |
| 801.25 | ICD9CM | Closed fracture of base of skull with subarachnoid, subdural, and extradural hemorrhage, with prolonged [more than 24 hours] loss of consciousness, without return to pre-existing conscious level | 1 |
| 801.26 | ICD9CM | Closed fracture of base of skull with subarachnoid, subdural, and extradural hemorrhage, with loss of consciousness of unspecified duration | 1 |
| 801.29 | ICD9CM | Closed fracture of base of skull with subarachnoid, subdural, and extradural hemorrhage, with concussion, unspecified | 1 |
| 801.30 | ICD9CM | Closed fracture of base of skull with other and unspecified intracranial hemorrhage, unspecified state of consciousness | 1 |
| 801.31 | ICD9CM | Closed fracture of base of skull with other and unspecified intracranial hemorrhage, with no loss of consciousness | 1 |
| 801.32 | ICD9CM | Closed fracture of base of skull with other and unspecified intracranial hemorrhage, with brief [less than one hour] loss of consciousness | 1 |
| 801.33 | ICD9CM | Closed fracture of base of skull with other and unspecified intracranial hemorrhage, with moderate [1-24 hours] loss of consciousness | 1 |
| 801.34 | ICD9CM | Closed fracture of base of skull with other and unspecified intracranial hemorrhage, with prolonged [more than 24 hours] loss of consciousness and return to pre-existing conscious level | 1 |
| 801.35 | ICD9CM | Closed fracture of base of skull with other and unspecified intracranial hemorrhage, with prolonged [more than 24 hours] loss of consciousness, without return to pre-existing conscious level | 1 |
| 801.36 | ICD9CM | Closed fracture of base of skull with other and unspecified intracranial hemorrhage, with loss of consciousness of unspecified duration | 1 |
| 801.39 | ICD9CM | Closed fracture of base of skull with other and unspecified intracranial hemorrhage, with concussion, unspecified | 1 |
| 801.70 | ICD9CM | Open fracture of base of skull with subarachnoid, subdural, and extradural hemorrhage, unspecified state of consciousness | 1 |
| 801.71 | ICD9CM | Open fracture of base of skull with subarachnoid, subdural, and extradural hemorrhage, with no loss of consciousness | 1 |
| 801.72 | ICD9CM | Open fracture of base of skull with subarachnoid, subdural, and extradural hemorrhage, with brief [less than one hour] loss of consciousness | 1 |
| 801.73 | ICD9CM | Open fracture of base of skull with subarachnoid, subdural, and extradural hemorrhage, with moderate [1-24 hours] loss of consciousness | 1 |
| 801.74 | ICD9CM | Open fracture of base of skull with subarachnoid, subdural, and extradural hemorrhage, with prolonged [more than 24 hours] loss of consciousness and return to pre-existing conscious level | 1 |
| 801.75 | ICD9CM | Open fracture of base of skull with subarachnoid, subdural, and extradural hemorrhage, with prolonged [more than 24 hours] loss of consciousness, without return to pre-existing conscious level | 1 |
| 801.76 | ICD9CM | Open fracture of base of skull with subarachnoid, subdural, and extradural hemorrhage, with loss of consciousness of unspecified duration | 1 |
| 801.79 | ICD9CM | Open fracture of base of skull with subarachnoid, subdural, and extradural hemorrhage, with concussion, unspecified | 1 |
| 801.80 | ICD9CM | Open fracture of base of skull with other and unspecified intracranial hemorrhage, unspecified state of consciousness | 1 |
| 801.81 | ICD9CM | Open fracture of base of skull with other and unspecified intracranial hemorrhage, with no loss of consciousness | 1 |
| 801.82 | ICD9CM | Open fracture of base of skull with other and unspecified intracranial hemorrhage, with brief [less than one hour] loss of consciousness | 1 |
| 801.83 | ICD9CM | Open fracture of base of skull with other and unspecified intracranial hemorrhage, with moderate [1-24 hours] loss of consciousness | 1 |
| 801.84 | ICD9CM | Open fracture of base of skull with other and unspecified intracranial hemorrhage, with prolonged [more than 24 hours] loss of consciousness and return to pre-existing conscious level | 1 |
| 801.85 | ICD9CM | Open fracture of base of skull with other and unspecified intracranial hemorrhage, with prolonged [more than 24 hours] loss of consciousness, without return to pre-existing conscious level | 1 |
| 801.86 | ICD9CM | Open fracture of base of skull with other and unspecified intracranial hemorrhage, with loss of consciousness of unspecified duration | 1 |
| 801.89 | ICD9CM | Open fracture of base of skull with other and unspecified intracranial hemorrhage, with concussion, unspecified | 1 |
| 803.20 | ICD9CM | Other closed skull fracture with subarachnoid, subdural, and extradural hemorrhage, unspecified state of consciousness | 1 |
| 803.21 | ICD9CM | Other closed skull fracture with subarachnoid, subdural, and extradural hemorrhage, with no loss of consciousness | 1 |
| 803.22 | ICD9CM | Other closed skull fracture with subarachnoid, subdural, and extradural hemorrhage, with brief [less than one hour] loss of consciousness | 1 |
| 803.23 | ICD9CM | Other closed skull fracture with subarachnoid, subdural, and extradural hemorrhage, with moderate [1-24 hours] loss of consciousness | 1 |
| 803.24 | ICD9CM | Other closed skull fracture with subarachnoid, subdural, and extradural hemorrhage, with prolonged [more than 24 hours] loss of consciousness and return to pre-existing conscious level | 1 |
| 803.25 | ICD9CM | Other closed skull fracture with subarachnoid, subdural, and extradural hemorrhage, with prolonged [more than 24 hours] loss of consciousness, without return to pre-existing conscious level | 1 |
| 803.26 | ICD9CM | Other closed skull fracture with subarachnoid, subdural, and extradural hemorrhage, with loss of consciousness of unspecified duration | 1 |
| 803.29 | ICD9CM | Other closed skull fracture with subarachnoid, subdural, and extradural hemorrhage, with concussion, unspecified | 1 |
| 803.30 | ICD9CM | Other closed skull fracture with other and unspecified intracranial hemorrhage, unspecified state of unconsciousness | 1 |
| 803.31 | ICD9CM | Other closed skull fracture with other and unspecified intracranial hemorrhage, with no loss of consciousness | 1 |
| 803.32 | ICD9CM | Other closed skull fracture with other and unspecified intracranial hemorrhage, with brief [less than one hour] loss of consciousness | 1 |
| 803.33 | ICD9CM | Other closed skull fracture with other and unspecified intracranial hemorrhage, with moderate [1-24 hours] loss of consciousness | 1 |
| 803.34 | ICD9CM | Other closed skull fracture with other and unspecified intracranial hemorrhage, with prolonged [more than 24 hours] loss of consciousness and return to pre-existing conscious level | 1 |
| 803.35 | ICD9CM | Other closed skull fracture with other and unspecified intracranial hemorrhage, with prolonged [more than 24 hours] loss of consciousness, without return to pre-existing conscious level | 1 |
| 803.36 | ICD9CM | Other closed skull fracture with other and unspecified intracranial hemorrhage, with loss of consciousness of unspecified duration | 1 |
| 803.39 | ICD9CM | Other closed skull fracture with other and unspecified intracranial hemorrhage, with concussion, unspecified | 1 |
| 803.70 | ICD9CM | Other open skull fracture with subarachnoid, subdural, and extradural hemorrhage, unspecified state of consciousness | 1 |
| 803.71 | ICD9CM | Other open skull fracture with subarachnoid, subdural, and extradural hemorrhage, with no loss of consciousness | 1 |
| 803.72 | ICD9CM | Other open skull fracture with subarachnoid, subdural, and extradural hemorrhage, with brief [less than one hour] loss of consciousness | 1 |
| 803.73 | ICD9CM | Other open skull fracture with subarachnoid, subdural, and extradural hemorrhage, with moderate [1-24 hours] loss of consciousness | 1 |
| 803.74 | ICD9CM | Other open skull fracture with subarachnoid, subdural, and extradural hemorrhage, with prolonged [more than 24 hours] loss of consciousness and return to pre-existing conscious level | 1 |
| 803.75 | ICD9CM | Other open skull fracture with subarachnoid, subdural, and extradural hemorrhage, with prolonged [more than 24 hours] loss of consciousness, without return to pre-existing conscious level | 1 |
| 803.76 | ICD9CM | Other open skull fracture with subarachnoid, subdural, and extradural hemorrhage, with loss of consciousness of unspecified duration | 1 |
| 803.79 | ICD9CM | Other open skull fracture with subarachnoid, subdural, and extradural hemorrhage, with concussion, unspecified | 1 |
| 803.80 | ICD9CM | Other open skull fracture with other and unspecified intracranial hemorrhage, unspecified state of consciousness | 1 |
| 803.81 | ICD9CM | Other open skull fracture with other and unspecified intracranial hemorrhage, with no loss of consciousness | 1 |
| 803.82 | ICD9CM | Other open skull fracture with other and unspecified intracranial hemorrhage, with brief [less than one hour] loss of consciousness | 1 |
| 803.83 | ICD9CM | Other open skull fracture with other and unspecified intracranial hemorrhage, with moderate [1-24 hours] loss of consciousness | 1 |
| 803.84 | ICD9CM | Other open skull fracture with other and unspecified intracranial hemorrhage, with prolonged [more than 24 hours] loss of consciousness and return to pre-existing conscious level | 1 |
| 803.85 | ICD9CM | Other open skull fracture with other and unspecified intracranial hemorrhage, with prolonged [more than 24 hours] loss of consciousness, without return to pre-existing conscious level | 1 |
| 803.86 | ICD9CM | Other open skull fracture with other and unspecified intracranial hemorrhage, with loss of consciousness of unspecified duration | 1 |
| 803.89 | ICD9CM | Other open skull fracture with other and unspecified intracranial hemorrhage, with concussion, unspecified | 1 |
| 804.20 | ICD9CM | Closed fractures involving skull or face with other bones with subarachnoid, subdural, and extradural hemorrhage, unspecified state of consciousness | 1 |
| 804.21 | ICD9CM | Closed fractures involving skull or face with other bones with subarachnoid, subdural, and extradural hemorrhage, with no loss of consciousness | 1 |
| 804.22 | ICD9CM | Closed fractures involving skull or face with other bones with subarachnoid, subdural, and extradural hemorrhage, with brief [less than one hour] loss of consciousness | 1 |
| 804.23 | ICD9CM | Closed fractures involving skull or face with other bones with subarachnoid, subdural, and extradural hemorrhage, with moderate [1-24 hours] loss of consciousness | 1 |
| 804.24 | ICD9CM | Closed fractures involving skull or face with other bones with subarachnoid, subdural, and extradural hemorrhage, with prolonged [more than 24 hours] loss of consciousness and return to pre-existing conscious level | 1 |
| 804.25 | ICD9CM | Closed fractures involving skull or face with other bones with subarachnoid, subdural, and extradural hemorrhage, with prolonged [more than 24 hours] loss of consciousness, without return to pre-existing conscious level | 1 |
| 804.26 | ICD9CM | Closed fractures involving skull or face with other bones with subarachnoid, subdural, and extradural hemorrhage, with loss of consciousness of unspecified duration | 1 |
| 804.29 | ICD9CM | Closed fractures involving skull or face with other bones with subarachnoid, subdural, and extradural hemorrhage, with concussion, unspecified | 1 |
| 804.30 | ICD9CM | Closed fractures involving skull or face with other bones, with other and unspecified intracranial hemorrhage, unspecified state of consciousness | 1 |
| 804.31 | ICD9CM | Closed fractures involving skull or face with other bones, with other and unspecified intracranial hemorrhage, with no loss of consciousness | 1 |
| 804.32 | ICD9CM | Closed fractures involving skull or face with other bones, with other and unspecified intracranial hemorrhage, with brief [less than one hour] loss of consciousness | 1 |
| 804.33 | ICD9CM | Closed fractures involving skull or face with other bones, with other and unspecified intracranial hemorrhage, with moderate [1-24 hours] loss of consciousness | 1 |
| 804.34 | ICD9CM | Closed fractures involving skull or face with other bones, with other and unspecified intracranial hemorrhage, with prolonged [more than 24 hours] loss of consciousness and return to pre- existing conscious level | 1 |
| 804.35 | ICD9CM | Closed fractures involving skull or face with other bones, with other and unspecified intracranial hemorrhage, with prolonged [more than 24 hours] loss of consciousness, without return to pre-existing conscious level | 1 |
| 804.36 | ICD9CM | Closed fractures involving skull or face with other bones, with other and unspecified intracranial hemorrhage, with loss of consciousness of unspecified duration | 1 |
| 804.39 | ICD9CM | Closed fractures involving skull or face with other bones, with other and unspecified intracranial hemorrhage, with concussion, unspecified | 1 |
| 804.70 | ICD9CM | Open fractures involving skull or face with other bones with subarachnoid, subdural, and extradural hemorrhage, unspecified state of consciousness | 1 |
| 804.71 | ICD9CM | Open fractures involving skull or face with other bones with subarachnoid, subdural, and extradural hemorrhage, with no loss of consciousness | 1 |
| 804.72 | ICD9CM | Open fractures involving skull or face with other bones with subarachnoid, subdural, and extradural hemorrhage, with brief [less than one hour] loss of consciousness | 1 |
| 804.73 | ICD9CM | Open fractures involving skull or face with other bones with subarachnoid, subdural, and extradural hemorrhage, with moderate [1-24 hours] loss of consciousness | 1 |
| 804.74 | ICD9CM | Open fractures involving skull or face with other bones with subarachnoid, subdural, and extradural hemorrhage, with prolonged [more than 24 hours] loss of consciousness and return to pre-existing conscious level | 1 |
| 804.75 | ICD9CM | Open fractures involving skull or face with other bones with subarachnoid, subdural, and extradural hemorrhage, with prolonged [more than 24 hours] loss of consciousness, without return to pre-existing conscious level | 1 |
| 804.76 | ICD9CM | Open fractures involving skull or face with other bones with subarachnoid, subdural, and extradural hemorrhage, with loss of consciousness of unspecified duration | 1 |
| 804.79 | ICD9CM | Open fractures involving skull or face with other bones with subarachnoid, subdural, and extradural hemorrhage, with concussion, unspecified | 1 |
| 804.80 | ICD9CM | Open fractures involving skull or face with other bones, with other and unspecified intracranial hemorrhage, unspecified state of consciousness | 1 |
| 804.81 | ICD9CM | Open fractures involving skull or face with other bones, with other and unspecified intracranial hemorrhage, with no loss of consciousness | 1 |
| 804.82 | ICD9CM | Open fractures involving skull or face with other bones, with other and unspecified intracranial hemorrhage, with brief [less than one hour] loss of consciousness | 1 |
| 804.83 | ICD9CM | Open fractures involving skull or face with other bones, with other and unspecified intracranial hemorrhage, with moderate [1-24 hours] loss of consciousness | 1 |
| 804.84 | ICD9CM | Open fractures involving skull or face with other bones, with other and unspecified intracranial hemorrhage, with prolonged [more than 24 hours] loss of consciousness and return to pre-existing conscious level | 1 |
| 804.85 | ICD9CM | Open fractures involving skull or face with other bones, with other and unspecified intracranial hemorrhage, with prolonged [more than 24 hours] loss consciousness, without return to pre-existing conscious level | 1 |
| 804.86 | ICD9CM | Open fractures involving skull or face with other bones, with other and unspecified intracranial hemorrhage, with loss of consciousness of unspecified duration | 1 |
| 804.89 | ICD9CM | Open fractures involving skull or face with other bones, with other and unspecified intracranial hemorrhage, with concussion, unspecified | 1 |
| 852.00 | ICD9CM | Subarachnoid hemorrhage following injury without mention of open intracranial wound, unspecified state of consciousness | 1 |
| 852.01 | ICD9CM | Subarachnoid hemorrhage following injury without mention of open intracranial wound, with no loss of consciousness | 1 |
| 852.02 | ICD9CM | Subarachnoid hemorrhage following injury without mention of open intracranial wound, with brief [less than one hour] loss of consciousness | 1 |
| 852.03 | ICD9CM | Subarachnoid hemorrhage following injury without mention of open intracranial wound, with moderate [1-24 hours] loss of consciousness | 1 |
| 852.04 | ICD9CM | Subarachnoid hemorrhage following injury without mention of open intracranial wound, with prolonged [more than 24 hours] loss of consciousness and return to pre-existing conscious level | 1 |
| 852.05 | ICD9CM | Subarachnoid hemorrhage following injury without mention of open intracranial wound, with prolonged [more than 24 hours] loss of consciousness without return to pre-existing conscious level | 1 |
| 852.06 | ICD9CM | Subarachnoid hemorrhage following injury without mention of open intracranial wound, with loss of consciousness of unspecified duration | 1 |
| 852.09 | ICD9CM | Subarachnoid hemorrhage following injury without mention of open intracranial wound, with concussion, unspecified | 1 |
| 852.10 | ICD9CM | Subarachnoid hemorrhage following injury with open intracranial wound, unspecified state of consciousness | 1 |
| 852.11 | ICD9CM | Subarachnoid hemorrhage following injury with open intracranial wound, with no loss of consciousness | 1 |
| 852.12 | ICD9CM | Subarachnoid hemorrhage following injury with open intracranial wound, with brief [less than one hour] loss of consciousness | 1 |
| 852.13 | ICD9CM | Subarachnoid hemorrhage following injury with open intracranial wound, with moderate [1-24 hours] loss of consciousness | 1 |
| 852.14 | ICD9CM | Subarachnoid hemorrhage following injury with open intracranial wound, with prolonged [more than 24 hours) loss of consciousness and return to pre-existing conscious level | 1 |
| 852.15 | ICD9CM | Subarachnoid hemorrhage following injury with open intracranial wound, with prolonged [more than 24 hours] loss of consciousness without return to pre-existing conscious level | 1 |
| 852.16 | ICD9CM | Subarachnoid hemorrhage following injury with open intracranial wound, with loss of consciousness of unspecified duration | 1 |
| 852.19 | ICD9CM | Subarachnoid hemorrhage following injury with open intracranial wound, with concussion, unspecified | 1 |
| 852.20 | ICD9CM | Subdural hemorrhage following injury without mention of open intracranial wound, unspecified state of consciousness | 1 |
| 852.21 | ICD9CM | Subdural hemorrhage following injury without mention of open intracranial wound, with no loss of consciousness | 1 |
| 852.22 | ICD9CM | Subdural hemorrhage following injury without mention of open intracranial wound, with brief [less than one hour] loss of consciousness | 1 |
| 852.23 | ICD9CM | Subdural hemorrhage following injury without mention of open intracranial wound, with moderate [1-24 hours] loss of consciousness | 1 |
| 852.24 | ICD9CM | Subdural hemorrhage following injury without mention of open intracranial wound, with prolonged [more than 24 hours] loss of consciousness and return to pre-existing conscious level | 1 |
| 852.25 | ICD9CM | Subdural hemorrhage following injury without mention of open intracranial wound, with prolonged [more than 24 hours] loss of consciousness without return to pre-existing conscious level | 1 |
| 852.26 | ICD9CM | Subdural hemorrhage following injury without mention of open intracranial wound, with loss of consciousness of unspecified duration | 1 |
| 852.29 | ICD9CM | Subdural hemorrhage following injury without mention of open intracranial wound, with concussion, unspecified | 1 |
| 852.30 | ICD9CM | Subdural hemorrhage following injury with open intracranial wound, unspecified state of consciousness | 1 |
| 852.31 | ICD9CM | Subdural hemorrhage following injury with open intracranial wound, with no loss of consciousness | 1 |
| 852.32 | ICD9CM | Subdural hemorrhage following injury with open intracranial wound, with brief [less than one hour] loss of consciousness | 1 |
| 852.33 | ICD9CM | Subdural hemorrhage following injury with open intracranial wound, with moderate [1-24 hours] loss of consciousness | 1 |
| 852.34 | ICD9CM | Subdural hemorrhage following injury with open intracranial wound, with prolonged [more than 24 hours] loss of consciousness and return to pre-existing conscious level | 1 |
| 852.35 | ICD9CM | Subdural hemorrhage following injury with open intracranial wound, with prolonged [more than 24 hours] loss of consciousness without return to pre-existing conscious level | 1 |
| 852.36 | ICD9CM | Subdural hemorrhage following injury with open intracranial wound, with loss of consciousness of unspecified duration | 1 |
| 852.39 | ICD9CM | Subdural hemorrhage following injury with open intracranial wound, with concussion, unspecified | 1 |
| 852.40 | ICD9CM | Extradural hemorrhage following injury without mention of open intracranial wound, unspecified state of consciousness | 1 |
| 852.41 | ICD9CM | Extradural hemorrhage following injury without mention of open intracranial wound, with no loss of consciousness | 1 |
| 852.42 | ICD9CM | Extradural hemorrhage following injury without mention of open intracranial wound, with brief [less than 1 hour] loss of consciousness | 1 |
| 852.43 | ICD9CM | Extradural hemorrhage following injury without mention of open intracranial wound, with moderate [1-24 hours] loss of consciousness | 1 |
| 852.44 | ICD9CM | Extradural hemorrhage following injury without mention of open intracranial wound, with prolonged [more than 24 hours] loss of consciousness and return to pre-existing conscious level | 1 |
| 852.45 | ICD9CM | Extradural hemorrhage following injury without mention of open intracranial wound, with prolonged [more than 24 hours] loss of consciousness without return to pre-existing conscious level | 1 |
| 852.46 | ICD9CM | Extradural hemorrhage following injury without mention of open intracranial wound, with loss of consciousness of unspecified duration | 1 |
| 852.49 | ICD9CM | Extradural hemorrhage following injury without mention of open intracranial wound, with concussion, unspecified | 1 |
| 852.50 | ICD9CM | Extradural hemorrhage following injury with open intracranial wound, unspecified state of consciousness | 1 |
| 852.51 | ICD9CM | Extradural hemorrhage following injury with open intracranial wound, with no loss of consciousness | 1 |
| 852.52 | ICD9CM | Extradural hemorrhage following injury with open intracranial wound, with brief [less than one hour] loss of consciousness | 1 |
| 852.53 | ICD9CM | Extradural hemorrhage following injury with open intracranial wound, with moderate [1-24 hours] loss of consciousness | 1 |
| 852.54 | ICD9CM | Extradural hemorrhage following injury with open intracranial wound, with prolonged [more than 24 hours] loss of consciousness and return to pre-existing conscious level | 1 |
| 852.55 | ICD9CM | Extradural hemorrhage following injury with open intracranial wound, with prolonged [more than 24 hours] loss of consciousness without return to pre-existing conscious level | 1 |
| 852.56 | ICD9CM | Extradural hemorrhage following injury with open intracranial wound, with loss of consciousness of unspecified duration | 1 |
| 852.59 | ICD9CM | Extradural hemorrhage following injury with open intracranial wound, with concussion, unspecified | 1 |
| 853.00 | ICD9CM | Other and unspecified intracranial hemorrhage following injury without mention of open intracranial wound, unspecified state of consciousness | 1 |
| 853.01 | ICD9CM | Other and unspecified intracranial hemorrhage following injury without mention of open intracranial wound, with no loss of consciousness | 1 |
| 853.02 | ICD9CM | Other and unspecified intracranial hemorrhage following injury without mention of open intracranial wound, with brief [less than one hour] loss of consciousness | 1 |
| 853.03 | ICD9CM | Other and unspecified intracranial hemorrhage following injury without mention of open intracranial wound, with moderate [1-24 hours] loss of consciousness | 1 |
| 853.04 | ICD9CM | Other and unspecified intracranial hemorrhage following injury without mention of open intracranial wound, with prolonged [more than 24 hours] loss of consciousness and return to pre- existing conscious level1 | 1 |
| 853.05 | ICD9CM | Other and unspecified intracranial hemorrhage following injury without mention of open intracranial wound, with prolonged [more than 24 hours] loss of consciousness without return to pre-existing conscious level | 1 |
| 853.06 | ICD9CM | Other and unspecified intracranial hemorrhage following injury without mention of open intracranial wound, with loss of consciousness of unspecified duration | 1 |
| 853.09 | ICD9CM | Other and unspecified intracranial hemorrhage following injury without mention of open intracranial wound, with concussion, unspecified | 1 |
| 853.10 | ICD9CM | Other and unspecified intracranial hemorrhage following injury with open intracranial wound, unspecified state of consciousness | 1 |
| 853.11 | ICD9CM | Other and unspecified intracranial hemorrhage following injury with open intracranial wound, with no loss of consciousness | 1 |
| 853.12 | ICD9CM | Other and unspecified intracranial hemorrhage following injury with open intracranial wound, with brief [less than one hour] loss of consciousness | 1 |
| 853.13 | ICD9CM | Other and unspecified intracranial hemorrhage following injury with open intracranial wound, with moderate [1-24 hours] loss of consciousness | 1 |
| 853.14 | ICD9CM | Other and unspecified intracranial hemorrhage following injury with open intracranial wound, with prolonged [more than 24 hours] loss of consciousness and return to pre-existing conscious level | 1 |
| 853.15 | ICD9CM | Other and unspecified intracranial hemorrhage following injury with open intracranial wound, with prolonged [more than 24 hours] loss of consciousness without return to pre-existing conscious level | 1 |
| 853.16 | ICD9CM | Other and unspecified intracranial hemorrhage following injury with open intracranial wound, with loss of consciousness of unspecified duration | 1 |
| 853.19 | ICD9CM | Other and unspecified intracranial hemorrhage following injury with open intracranial wound, with concussion, unspecified | 1 |
| 958.90 | ICD9CM | Compartment syndrome, unspecified | 1 |
| 958.91 | ICD9CM | Traumatic compartment syndrome of upper extremity | 1 |
| 958.92 | ICD9CM | Traumatic compartment syndrome of lower extremity | 1 |
| 958.93 | ICD9CM | Traumatic compartment syndrome of abdomen | 1 |
| 958.99 | ICD9CM | Traumatic compartment syndrome of other sites | 1 |
| S06.34 | ICD10CM | Traumatic hemorrhage of right cerebrum | 1 |
| S06.340 | ICD10CM | Traumatic hemorrhage of right cerebrum without loss of consciousness | 1 |
| S06.340A | ICD10CM | Traumatic hemorrhage of right cerebrum without loss of consciousness, initial encounter | 1 |
| S06.341 | ICD10CM | Traumatic hemorrhage of right cerebrum with loss of consciousness of 30 minutes or less | 1 |
| S06.341A | ICD10CM | Traumatic hemorrhage of right cerebrum with loss of consciousness of 30 minutes or less, initial encounter | 1 |
| S06.342 | ICD10CM | Traumatic hemorrhage of right cerebrum with loss of consciousness of 31 minutes to 59 minutes | 1 |
| S06.342A | ICD10CM | Traumatic hemorrhage of right cerebrum with loss of consciousness of 31 minutes to 59 minutes, initial encounter | 1 |
| S06.343 | ICD10CM | Traumatic hemorrhage of right cerebrum with loss of consciousness of 1 hours to 5 hours 59 minutes | 1 |
| S06.343A | ICD10CM | Traumatic hemorrhage of right cerebrum with loss of consciousness of 1 hours to 5 hours 59 minutes, initial encounter | 1 |
| S06.344 | ICD10CM | Traumatic hemorrhage of right cerebrum with loss of consciousness of 6 hours to 24 hours | 1 |
| S06.344A | ICD10CM | Traumatic hemorrhage of right cerebrum with loss of consciousness of 6 hours to 24 hours, initial encounter | 1 |
| S06.345 | ICD10CM | Traumatic hemorrhage of right cerebrum with loss of consciousness greater than 24 hours with return to pre-existing conscious level | 1 |
| S06.345A | ICD10CM | Traumatic hemorrhage of right cerebrum with loss of consciousness greater than 24 hours with return to pre-existing conscious level, initial encounter | 1 |
| S06.346 | ICD10CM | Traumatic hemorrhage of right cerebrum with loss of consciousness greater than 24 hours without return to pre-existing conscious level with patient surviving | 1 |
| S06.346A | ICD10CM | Traumatic hemorrhage of right cerebrum with loss of consciousness greater than 24 hours without return to pre-existing conscious level with patient surviving, initial encounter | 1 |
| S06.347 | ICD10CM | Traumatic hemorrhage of right cerebrum with loss of consciousness of any duration with death due to brain injury prior to regaining consciousness | 1 |
| S06.347A | ICD10CM | Traumatic hemorrhage of right cerebrum with loss of consciousness of any duration with death due to brain injury prior to regaining consciousness, initial encounter | 1 |
| S06.348 | ICD10CM | Traumatic hemorrhage of right cerebrum with loss of consciousness of any duration with death due to other cause prior to regaining consciousness | 1 |
| S06.348A | ICD10CM | Traumatic hemorrhage of right cerebrum with loss of consciousness of any duration with death due to other cause prior to regaining consciousness, initial encounter | 1 |
| S06.349 | ICD10CM | Traumatic hemorrhage of right cerebrum with loss of consciousness of unspecified duration | 1 |
| S06.349A | ICD10CM | Traumatic hemorrhage of right cerebrum with loss of consciousness of unspecified duration, initial encounter | 1 |
| S06.35 | ICD10CM | Traumatic hemorrhage of left cerebrum | 1 |
| S06.350 | ICD10CM | Traumatic hemorrhage of left cerebrum without loss of consciousness | 1 |
| S06.350A | ICD10CM | Traumatic hemorrhage of left cerebrum without loss of consciousness, initial encounter | 1 |
| S06.351 | ICD10CM | Traumatic hemorrhage of left cerebrum with loss of consciousness of 30 minutes or less | 1 |
| S06.351A | ICD10CM | Traumatic hemorrhage of left cerebrum with loss of consciousness of 30 minutes or less, initial encounter | 1 |
| S06.352 | ICD10CM | Traumatic hemorrhage of left cerebrum with loss of consciousness of 31 minutes to 59 minutes | 1 |
| S06.352A | ICD10CM | Traumatic hemorrhage of left cerebrum with loss of consciousness of 31 minutes to 59 minutes, initial encounter | 1 |
| S06.353 | ICD10CM | Traumatic hemorrhage of left cerebrum with loss of consciousness of 1 hours to 5 hours 59 minutes | 1 |
| S06.353A | ICD10CM | Traumatic hemorrhage of left cerebrum with loss of consciousness of 1 hours to 5 hours 59 minutes, initial encounter | 1 |
| S06.354 | ICD10CM | Traumatic hemorrhage of left cerebrum with loss of consciousness of 6 hours to 24 hours | 1 |
| S06.354A | ICD10CM | Traumatic hemorrhage of left cerebrum with loss of consciousness of 6 hours to 24 hours, initial encounter | 1 |
| S06.355 | ICD10CM | Traumatic hemorrhage of left cerebrum with loss of consciousness greater than 24 hours with return to pre-existing conscious level | 1 |
| S06.355A | ICD10CM | Traumatic hemorrhage of left cerebrum with loss of consciousness greater than 24 hours with return to pre-existing conscious level, initial encounter | 1 |
| S06.356 | ICD10CM | Traumatic hemorrhage of left cerebrum with loss of consciousness greater than 24 hours without return to pre-existing conscious level with patient surviving | 1 |
| S06.356A | ICD10CM | Traumatic hemorrhage of left cerebrum with loss of consciousness greater than 24 hours without return to pre-existing conscious level with patient surviving, initial encounter | 1 |
| S06.357 | ICD10CM | Traumatic hemorrhage of left cerebrum with loss of consciousness of any duration with death due to brain injury prior to regaining consciousness | 1 |
| S06.357A | ICD10CM | Traumatic hemorrhage of left cerebrum with loss of consciousness of any duration with death due to brain injury prior to regaining consciousness, initial encounter | 1 |
| S06.358 | ICD10CM | Traumatic hemorrhage of left cerebrum with loss of consciousness of any duration with death due to other cause prior to regaining consciousness | 1 |
| S06.358A | ICD10CM | Traumatic hemorrhage of left cerebrum with loss of consciousness of any duration with death due to other cause prior to regaining consciousness, initial encounter | 1 |
| S06.359 | ICD10CM | Traumatic hemorrhage of left cerebrum with loss of consciousness of unspecified duration | 1 |
| S06.359A | ICD10CM | Traumatic hemorrhage of left cerebrum with loss of consciousness of unspecified duration, initial encounter | 1 |
| S06.36 | ICD10CM | Traumatic hemorrhage of cerebrum, unspecified | 1 |
| S06.360 | ICD10CM | Traumatic hemorrhage of cerebrum, unspecified, without loss of consciousness | 1 |
| S06.360A | ICD10CM | Traumatic hemorrhage of cerebrum, unspecified, without loss of consciousness, initial encounter | 1 |
| S06.361 | ICD10CM | Traumatic hemorrhage of cerebrum, unspecified, with loss of consciousness of 30 minutes or less | 1 |
| S06.361A | ICD10CM | Traumatic hemorrhage of cerebrum, unspecified, with loss of consciousness of 30 minutes or less, initial encounter | 1 |
| S06.362 | ICD10CM | Traumatic hemorrhage of cerebrum, unspecified, with loss of consciousness of 31 minutes to 59 minutes | 1 |
| S06.362A | ICD10CM | Traumatic hemorrhage of cerebrum, unspecified, with loss of consciousness of 31 minutes to 59 minutes, initial encounter | 1 |
| S06.363 | ICD10CM | Traumatic hemorrhage of cerebrum, unspecified, with loss of consciousness of 1 hours to 5 hours 59 minutes | 1 |
| S06.363A | ICD10CM | Traumatic hemorrhage of cerebrum, unspecified, with loss of consciousness of 1 hours to 5 hours 59 minutes, initial encounter | 1 |
| S06.364 | ICD10CM | Traumatic hemorrhage of cerebrum, unspecified, with loss of consciousness of 6 hours to 24 hours | 1 |
| S06.364A | ICD10CM | Traumatic hemorrhage of cerebrum, unspecified, with loss of consciousness of 6 hours to 24 hours, initial encounter | 1 |
| S06.365 | ICD10CM | Traumatic hemorrhage of cerebrum, unspecified, with loss of consciousness greater than 24 hours with return to pre-existing conscious level | 1 |
| S06.365A | ICD10CM | Traumatic hemorrhage of cerebrum, unspecified, with loss of consciousness greater than 24 hours with return to pre-existing conscious level, initial encounter | 1 |
| S06.366 | ICD10CM | Traumatic hemorrhage of cerebrum, unspecified, with loss of consciousness greater than 24 hours without return to pre-existing conscious level with patient surviving | 1 |
| S06.366A | ICD10CM | Traumatic hemorrhage of cerebrum, unspecified, with loss of consciousness greater than 24 hours without return to pre-existing conscious level with patient surviving, initial encounter | 1 |
| S06.367 | ICD10CM | Traumatic hemorrhage of cerebrum, unspecified, with loss of consciousness of any duration with death due to brain injury prior to regaining consciousness | 1 |
| S06.367A | ICD10CM | Traumatic hemorrhage of cerebrum, unspecified, with loss of consciousness of any duration with death due to brain injury prior to regaining consciousness, initial encounter | 1 |
| S06.368 | ICD10CM | Traumatic hemorrhage of cerebrum, unspecified, with loss of consciousness of any duration with death due to other cause prior to regaining consciousness | 1 |
| S06.368A | ICD10CM | Traumatic hemorrhage of cerebrum, unspecified, with loss of consciousness of any duration with death due to other cause prior to regaining consciousness, initial encounter | 1 |
| S06.369 | ICD10CM | Traumatic hemorrhage of cerebrum, unspecified, with loss of consciousness of unspecified duration | 1 |
| S06.369A | ICD10CM | Traumatic hemorrhage of cerebrum, unspecified, with loss of consciousness of unspecified duration, initial encounter | 1 |
| S06.4 | ICD10CM | Epidural hemorrhage | 1 |
| S06.4X | ICD10CM | Epidural hemorrhage | 1 |
| S06.4X0 | ICD10CM | Epidural hemorrhage without loss of consciousness | 1 |
| S06.4X0A | ICD10CM | Epidural hemorrhage without loss of consciousness, initial encounter | 1 |
| S06.4X1 | ICD10CM | Epidural hemorrhage with loss of consciousness of 30 minutes or less | 1 |
| S06.4X1A | ICD10CM | Epidural hemorrhage with loss of consciousness of 30 minutes or less, initial encounter | 1 |
| S06.4X2 | ICD10CM | Epidural hemorrhage with loss of consciousness of 31 minutes to 59 minutes | 1 |
| S06.4X2A | ICD10CM | Epidural hemorrhage with loss of consciousness of 31 minutes to 59 minutes, initial encounter | 1 |
| S06.4X3 | ICD10CM | Epidural hemorrhage with loss of consciousness of 1 hour to 5 hours 59 minutes | 1 |
| S06.4X3A | ICD10CM | Epidural hemorrhage with loss of consciousness of 1 hour to 5 hours 59 minutes, initial encounter | 1 |
| S06.4X4 | ICD10CM | Epidural hemorrhage with loss of consciousness of 6 hours to 24 hours | 1 |
| S06.4X4A | ICD10CM | Epidural hemorrhage with loss of consciousness of 6 hours to 24 hours, initial encounter | 1 |
| S06.4X5 | ICD10CM | Epidural hemorrhage with loss of consciousness greater than 24 hours with return to pre-existing conscious level | 1 |
| S06.4X5A | ICD10CM | Epidural hemorrhage with loss of consciousness greater than 24 hours with return to pre-existing conscious level, initial encounter | 1 |
| S06.4X6 | ICD10CM | Epidural hemorrhage with loss of consciousness greater than 24 hours without return to pre-existing conscious level with patient surviving | 1 |
| S06.4X6A | ICD10CM | Epidural hemorrhage with loss of consciousness greater than 24 hours without return to pre-existing conscious level with patient surviving, initial encounter | 1 |
| S06.4X7 | ICD10CM | Epidural hemorrhage with loss of consciousness of any duration with death due to brain injury prior to regaining consciousness | 1 |
| S06.4X7A | ICD10CM | Epidural hemorrhage with loss of consciousness of any duration with death due to brain injury prior to regaining consciousness, initial encounter | 1 |
| S06.4X8 | ICD10CM | Epidural hemorrhage with loss of consciousness of any duration with death due to other causes prior to regaining consciousness | 1 |
| S06.4X8A | ICD10CM | Epidural hemorrhage with loss of consciousness of any duration with death due to other causes prior to regaining consciousness, initial encounter | 1 |
| S06.4X9 | ICD10CM | Epidural hemorrhage with loss of consciousness of unspecified duration | 1 |
| S06.4X9A | ICD10CM | Epidural hemorrhage with loss of consciousness of unspecified duration, initial encounter | 1 |
| S06.5 | ICD10CM | Traumatic subdural hemorrhage | 1 |
| S06.5X | ICD10CM | Traumatic subdural hemorrhage | 1 |
| S06.5X0 | ICD10CM | Traumatic subdural hemorrhage without loss of consciousness | 1 |
| S06.5X0A | ICD10CM | Traumatic subdural hemorrhage without loss of consciousness, initial encounter | 1 |
| S06.5X1 | ICD10CM | Traumatic subdural hemorrhage with loss of consciousness of 30 minutes or less | 1 |
| S06.5X1A | ICD10CM | Traumatic subdural hemorrhage with loss of consciousness of 30 minutes or less, initial encounter | 1 |
| S06.5X2 | ICD10CM | Traumatic subdural hemorrhage with loss of consciousness of 31 minutes to 59 minutes | 1 |
| S06.5X2A | ICD10CM | Traumatic subdural hemorrhage with loss of consciousness of 31 minutes to 59 minutes, initial encounter | 1 |
| S06.5X3 | ICD10CM | Traumatic subdural hemorrhage with loss of consciousness of 1 hour to 5 hours 59 minutes | 1 |
| S06.5X3A | ICD10CM | Traumatic subdural hemorrhage with loss of consciousness of 1 hour to 5 hours 59 minutes, initial encounter | 1 |
| S06.5X4 | ICD10CM | Traumatic subdural hemorrhage with loss of consciousness of 6 hours to 24 hours | 1 |
| S06.5X4A | ICD10CM | Traumatic subdural hemorrhage with loss of consciousness of 6 hours to 24 hours, initial encounter | 1 |
| S06.5X5 | ICD10CM | Traumatic subdural hemorrhage with loss of consciousness greater than 24 hours with return to pre-existing conscious level | 1 |
| S06.5X5A | ICD10CM | Traumatic subdural hemorrhage with loss of consciousness greater than 24 hours with return to pre-existing conscious level, initial encounter | 1 |
| S06.5X6 | ICD10CM | Traumatic subdural hemorrhage with loss of consciousness greater than 24 hours without return to pre-existing conscious level with patient surviving | 1 |
| S06.5X6A | ICD10CM | Traumatic subdural hemorrhage with loss of consciousness greater than 24 hours without return to pre-existing conscious level with patient surviving, initial encounter | 1 |
| S06.5X7 | ICD10CM | Traumatic subdural hemorrhage with loss of consciousness of any duration with death due to brain injury before regaining consciousness | 1 |
| S06.5X7A | ICD10CM | Traumatic subdural hemorrhage with loss of consciousness of any duration with death due to brain injury before regaining consciousness, initial encounter | 1 |
| S06.5X8 | ICD10CM | Traumatic subdural hemorrhage with loss of consciousness of any duration with death due to other cause before regaining consciousness | 1 |
| S06.5X8A | ICD10CM | Traumatic subdural hemorrhage with loss of consciousness of any duration with death due to other cause before regaining consciousness, initial encounter | 1 |
| S06.5X9 | ICD10CM | Traumatic subdural hemorrhage with loss of consciousness of unspecified duration | 1 |
| S06.5X9A | ICD10CM | Traumatic subdural hemorrhage with loss of consciousness of unspecified duration, initial encounter | 1 |
| S06.6 | ICD10CM | Traumatic subarachnoid hemorrhage | 1 |
| S06.6X | ICD10CM | Traumatic subarachnoid hemorrhage | 1 |
| S06.6X0 | ICD10CM | Traumatic subarachnoid hemorrhage without loss of consciousness | 1 |
| S06.6X0A | ICD10CM | Traumatic subarachnoid hemorrhage without loss of consciousness, initial encounter | 1 |
| S06.6X1 | ICD10CM | Traumatic subarachnoid hemorrhage with loss of consciousness of 30 minutes or less | 1 |
| S06.6X1A | ICD10CM | Traumatic subarachnoid hemorrhage with loss of consciousness of 30 minutes or less, initial encounter | 1 |
| S06.6X2 | ICD10CM | Traumatic subarachnoid hemorrhage with loss of consciousness of 31 minutes to 59 minutes | 1 |
| S06.6X2A | ICD10CM | Traumatic subarachnoid hemorrhage with loss of consciousness of 31 minutes to 59 minutes, initial encounter | 1 |
| S06.6X3 | ICD10CM | Traumatic subarachnoid hemorrhage with loss of consciousness of 1 hour to 5 hours 59 minutes | 1 |
| S06.6X3A | ICD10CM | Traumatic subarachnoid hemorrhage with loss of consciousness of 1 hour to 5 hours 59 minutes, initial encounter | 1 |
| S06.6X4 | ICD10CM | Traumatic subarachnoid hemorrhage with loss of consciousness of 6 hours to 24 hours | 1 |
| S06.6X4A | ICD10CM | Traumatic subarachnoid hemorrhage with loss of consciousness of 6 hours to 24 hours, initial encounter | 1 |
| S06.6X5 | ICD10CM | Traumatic subarachnoid hemorrhage with loss of consciousness greater than 24 hours with return to pre-existing conscious level | 1 |
| S06.6X5A | ICD10CM | Traumatic subarachnoid hemorrhage with loss of consciousness greater than 24 hours with return to pre-existing conscious level, initial encounter | 1 |
| S06.6X6 | ICD10CM | Traumatic subarachnoid hemorrhage with loss of consciousness greater than 24 hours without return to pre-existing conscious level with patient surviving | 1 |
| S06.6X6A | ICD10CM | Traumatic subarachnoid hemorrhage with loss of consciousness greater than 24 hours without return to pre-existing conscious level with patient surviving, initial encounter | 1 |
| S06.6X7 | ICD10CM | Traumatic subarachnoid hemorrhage with loss of consciousness of any duration with death due to brain injury prior to regaining consciousness | 1 |
| S06.6X7A | ICD10CM | Traumatic subarachnoid hemorrhage with loss of consciousness of any duration with death due to brain injury prior to regaining consciousness, initial encounter | 1 |
| S06.6X8 | ICD10CM | Traumatic subarachnoid hemorrhage with loss of consciousness of any duration with death due to other cause prior to regaining consciousness | 1 |
| S06.6X8A | ICD10CM | Traumatic subarachnoid hemorrhage with loss of consciousness of any duration with death due to other cause prior to regaining consciousness, initial encounter | 1 |
| S06.6X9 | ICD10CM | Traumatic subarachnoid hemorrhage with loss of consciousness of unspecified duration | 1 |
| S06.6X9A | ICD10CM | Traumatic subarachnoid hemorrhage with loss of consciousness of unspecified duration, initial encounter | 1 |
| S26.00XA | ICD10CM | Unspecified injury of heart with hemopericardium, initial encounter | 1 |
| S26.01XA | ICD10CM | Contusion of heart with hemopericardium, initial encounter | 1 |
| S26.020A | ICD10CM | Mild laceration of heart with hemopericardium, initial encounter | 1 |
| S26.09XA | ICD10CM | Other injury of heart with hemopericardium, initial encounter | 1 |
| T79.A | ICD10CM | Traumatic compartment syndrome | 1 |
| T79.A0 | ICD10CM | Compartment syndrome, unspecified | 1 |
| T79.A0XA | ICD10CM | Compartment syndrome, unspecified, initial encounter | 1 |
| T79.A1 | ICD10CM | Traumatic compartment syndrome of upper extremity | 1 |
| T79.A11 | ICD10CM | Traumatic compartment syndrome of right upper extremity | 1 |
| T79.A11A | ICD10CM | Traumatic compartment syndrome of right upper extremity, initial encounter | 1 |
| T79.A12 | ICD10CM | Traumatic compartment syndrome of left upper extremity | 1 |
| T79.A12A | ICD10CM | Traumatic compartment syndrome of left upper extremity, initial encounter | 1 |
| T79.A19 | ICD10CM | Traumatic compartment syndrome of unspecified upper extremity | 1 |
| T79.A19A | ICD10CM | Traumatic compartment syndrome of unspecified upper extremity, initial encounter | 1 |
| T79.A2 | ICD10CM | Traumatic compartment syndrome of lower extremity | 1 |
| T79.A21 | ICD10CM | Traumatic compartment syndrome of right lower extremity | 1 |
| T79.A21A | ICD10CM | Traumatic compartment syndrome of right lower extremity, initial encounter | 1 |
| T79.A22 | ICD10CM | Traumatic compartment syndrome of left lower extremity | 1 |
| T79.A22A | ICD10CM | Traumatic compartment syndrome of left lower extremity, initial encounter | 1 |
| T79.A29 | ICD10CM | Traumatic compartment syndrome of unspecified lower extremity | 1 |
| T79.A29A | ICD10CM | Traumatic compartment syndrome of unspecified lower extremity, initial encounter | 1 |
| T79.A3 | ICD10CM | Traumatic compartment syndrome of abdomen | 1 |
| T79.A3XA | ICD10CM | Traumatic compartment syndrome of abdomen, initial encounter | 1 |
| T79.A9 | ICD10CM | Traumatic compartment syndrome of other sites | 1 |
| T79.A9XA | ICD10CM | Traumatic compartment syndrome of other sites, initial encounter | 1 |
| 360.43 | ICD9CM | Hemophthalmos, except current injury |  |
| 362.43 | ICD9CM | Hemorrhagic detachment of retinal pigment epithelium |  |
| 362.81 | ICD9CM | Retinal hemorrhage |  |
| 363.6 | ICD9CM | Choroidal hemorrhage and rupture |  |
| 363.61 | ICD9CM | Choroidal hemorrhage, unspecified |  |
| 363.62 | ICD9CM | Expulsive choroidal hemorrhage |  |
| 363.63 | ICD9CM | Choroidal rupture |  |
| 363.72 | ICD9CM | Hemorrhagic choroidal detachment |  |
| 364.41 | ICD9CM | Hyphema of iris and ciliary body |  |
| 376.32 | ICD9CM | Orbital hemorrhage |  |
| 379.23 | ICD9CM | Vitreous hemorrhage |  |
| 423.0 | ICD9CM | Hemopericardium |  |
| 423.3 | ICD9CM | Cardiac tamponade |  |
| 430 | ICD9CM | Subarachnoid hemorrhage |  |
| 431 | ICD9CM | Intracerebral hemorrhage |  |
| 432 | ICD9CM | Other and unspecified intracranial hemorrhage |  |
| 432.0 | ICD9CM | Nontraumatic extradural hemorrhage |  |
| 432.1 | ICD9CM | Subdural hemorrhage |  |
| 432.9 | ICD9CM | Unspecified intracranial hemorrhage |  |
| 441.1 | ICD9CM | Thoracic aneurysm, ruptured |  |
| 441.3 | ICD9CM | Abdominal aneurysm, ruptured |  |
| 441.5 | ICD9CM | Aortic aneurysm of unspecified site, ruptured |  |
| 441.6 | ICD9CM | Thoracoabdominal aneurysm, ruptured |  |
| 719.10 | ICD9CM | Hemarthrosis, site unspecified |  |
| 719.11 | ICD9CM | Hemarthrosis, shoulder region |  |
| 719.12 | ICD9CM | Hemarthrosis, upper arm |  |
| 719.13 | ICD9CM | Hemarthrosis, forearm |  |
| 719.14 | ICD9CM | Hemarthrosis, hand |  |
| 719.15 | ICD9CM | Hemarthrosis, pelvic region and thigh |  |
| 719.16 | ICD9CM | Hemarthrosis, lower leg |  |
| 719.17 | ICD9CM | Hemarthrosis, ankle and foot |  |
| 719.18 | ICD9CM | Hemarthrosis, other specified sites |  |
| 719.19 | ICD9CM | Hemarthrosis, multiple sites |  |
| 729.71 | ICD9CM | Nontraumatic compartment syndrome of upper extremity |  |
| 729.72 | ICD9CM | Nontraumatic compartment syndrome of lower extremity |  |
| 729.73 | ICD9CM | Nontraumatic compartment syndrome of abdomen |  |
| 729.79 | ICD9CM | Nontraumatic compartment syndrome of other sites |  |
| 998.11 | ICD9CM | Hemorrhage complicating a procedure |  |
| H05.23 | ICD10CM | Hemorrhage of orbit |  |
| H05.231 | ICD10CM | Hemorrhage of right orbit |  |
| H05.232 | ICD10CM | Hemorrhage of left orbit |  |
| H05.233 | ICD10CM | Hemorrhage of bilateral orbit |  |
| H05.239 | ICD10CM | Hemorrhage of unspecified orbit |  |
| H21.0 | ICD10CM | Hyphema |  |
| H21.00 | ICD10CM | Hyphema, unspecified eye |  |
| H21.01 | ICD10CM | Hyphema, right eye |  |
| H21.02 | ICD10CM | Hyphema, left eye |  |
| H21.03 | ICD10CM | Hyphema, bilateral |  |
| H31.30 | ICD10CM | Unspecified choroidal hemorrhage |  |
| H31.301 | ICD10CM | Unspecified choroidal hemorrhage, right eye |  |
| H31.302 | ICD10CM | Unspecified choroidal hemorrhage, left eye |  |
| H31.303 | ICD10CM | Unspecified choroidal hemorrhage, bilateral |  |
| H31.309 | ICD10CM | Unspecified choroidal hemorrhage, unspecified eye |  |
| H31.31 | ICD10CM | Expulsive choroidal hemorrhage |  |
| H31.311 | ICD10CM | Expulsive choroidal hemorrhage, right eye |  |
| H31.312 | ICD10CM | Expulsive choroidal hemorrhage, left eye |  |
| H31.313 | ICD10CM | Expulsive choroidal hemorrhage, bilateral |  |
| H31.319 | ICD10CM | Expulsive choroidal hemorrhage, unspecified eye |  |
| H31.41 | ICD10CM | Hemorrhagic choroidal detachment |  |
| H31.411 | ICD10CM | Hemorrhagic choroidal detachment, right eye |  |
| H31.412 | ICD10CM | Hemorrhagic choroidal detachment, left eye |  |
| H31.413 | ICD10CM | Hemorrhagic choroidal detachment, bilateral |  |
| H31.419 | ICD10CM | Hemorrhagic choroidal detachment, unspecified eye |  |
| H35.6 | ICD10CM | Retinal hemorrhage |  |
| H35.60 | ICD10CM | Retinal hemorrhage, unspecified eye |  |
| H35.61 | ICD10CM | Retinal hemorrhage, right eye |  |
| H35.62 | ICD10CM | Retinal hemorrhage, left eye |  |
| H35.63 | ICD10CM | Retinal hemorrhage, bilateral |  |
| H35.73 | ICD10CM | Hemorrhagic detachment of retinal pigment epithelium |  |
| H35.731 | ICD10CM | Hemorrhagic detachment of retinal pigment epithelium, right eye |  |
| H35.732 | ICD10CM | Hemorrhagic detachment of retinal pigment epithelium, left eye |  |
| H35.733 | ICD10CM | Hemorrhagic detachment of retinal pigment epithelium, bilateral |  |
| H35.739 | ICD10CM | Hemorrhagic detachment of retinal pigment epithelium, unspecified eye |  |
| H43.1 | ICD10CM | Vitreous hemorrhage |  |
| H43.10 | ICD10CM | Vitreous hemorrhage, unspecified eye |  |
| H43.11 | ICD10CM | Vitreous hemorrhage, right eye |  |
| H43.12 | ICD10CM | Vitreous hemorrhage, left eye |  |
| H43.13 | ICD10CM | Vitreous hemorrhage, bilateral |  |
| H44.81 | ICD10CM | Hemophthalmos |  |
| H44.811 | ICD10CM | Hemophthalmos, right eye |  |
| H44.812 | ICD10CM | Hemophthalmos, left eye |  |
| H44.813 | ICD10CM | Hemophthalmos, bilateral |  |
| H44.819 | ICD10CM | Hemophthalmos, unspecified eye |  |
| I23.0 | ICD10CM | Hemopericardium as current complication following acute myocardial infarction |  |
| I31.2 | ICD10CM | Hemopericardium, not elsewhere classified |  |
| I31.4 | ICD10CM | Cardiac tamponade |  |
| I60 | ICD10CM | Nontraumatic subarachnoid hemorrhage |  |
| I60.0 | ICD10CM | Nontraumatic subarachnoid hemorrhage from carotid siphon and bifurcation |  |
| I60.00 | ICD10CM | Nontraumatic subarachnoid hemorrhage from unspecified carotid siphon and bifurcation |  |
| I60.01 | ICD10CM | Nontraumatic subarachnoid hemorrhage from right carotid siphon and bifurcation |  |
| I60.02 | ICD10CM | Nontraumatic subarachnoid hemorrhage from left carotid siphon and bifurcation |  |
| I60.1 | ICD10CM | Nontraumatic subarachnoid hemorrhage from middle cerebral artery |  |
| I60.10 | ICD10CM | Nontraumatic subarachnoid hemorrhage from unspecified middle cerebral artery |  |
| I60.11 | ICD10CM | Nontraumatic subarachnoid hemorrhage from right middle cerebral artery |  |
| I60.12 | ICD10CM | Nontraumatic subarachnoid hemorrhage from left middle cerebral artery |  |
| I60.2 | ICD10CM | Nontraumatic subarachnoid hemorrhage from anterior communicating artery |  |
| I60.3 | ICD10CM | Nontraumatic subarachnoid hemorrhage from posterior communicating artery |  |
| I60.30 | ICD10CM | Nontraumatic subarachnoid hemorrhage from unspecified posterior communicating artery |  |
| I60.31 | ICD10CM | Nontraumatic subarachnoid hemorrhage from right posterior communicating artery |  |
| I60.32 | ICD10CM | Nontraumatic subarachnoid hemorrhage from left posterior communicating artery |  |
| I60.4 | ICD10CM | Nontraumatic subarachnoid hemorrhage from basilar artery |  |
| I60.5 | ICD10CM | Nontraumatic subarachnoid hemorrhage from vertebral artery |  |
| I60.50 | ICD10CM | Nontraumatic subarachnoid hemorrhage from unspecified vertebral artery |  |
| I60.51 | ICD10CM | Nontraumatic subarachnoid hemorrhage from right vertebral artery |  |
| I60.52 | ICD10CM | Nontraumatic subarachnoid hemorrhage from left vertebral artery |  |
| I60.6 | ICD10CM | Nontraumatic subarachnoid hemorrhage from other intracranial arteries |  |
| I60.7 | ICD10CM | Nontraumatic subarachnoid hemorrhage from unspecified intracranial artery |  |
| I60.8 | ICD10CM | Other nontraumatic subarachnoid hemorrhage |  |
| I60.9 | ICD10CM | Nontraumatic subarachnoid hemorrhage, unspecified |  |
| I61 | ICD10CM | Nontraumatic intracerebral hemorrhage |  |
| I61.0 | ICD10CM | Nontraumatic intracerebral hemorrhage in hemisphere, subcortical |  |
| I61.1 | ICD10CM | Nontraumatic intracerebral hemorrhage in hemisphere, cortical |  |
| I61.2 | ICD10CM | Nontraumatic intracerebral hemorrhage in hemisphere, unspecified |  |
| I61.3 | ICD10CM | Nontraumatic intracerebral hemorrhage in brain stem |  |
| I61.4 | ICD10CM | Nontraumatic intracerebral hemorrhage in cerebellum |  |
| I61.5 | ICD10CM | Nontraumatic intracerebral hemorrhage, intraventricular |  |
| I61.6 | ICD10CM | Nontraumatic intracerebral hemorrhage, multiple localized |  |
| I61.8 | ICD10CM | Other nontraumatic intracerebral hemorrhage |  |
| I61.9 | ICD10CM | Nontraumatic intracerebral hemorrhage, unspecified |  |
| I62 | ICD10CM | Other and unspecified nontraumatic intracranial hemorrhage |  |
| I62.0 | ICD10CM | Nontraumatic subdural hemorrhage |  |
| I62.00 | ICD10CM | Nontraumatic subdural hemorrhage, unspecified |  |
| I62.01 | ICD10CM | Nontraumatic acute subdural hemorrhage |  |
| I62.02 | ICD10CM | Nontraumatic subacute subdural hemorrhage |  |
| I62.03 | ICD10CM | Nontraumatic chronic subdural hemorrhage |  |
| I62.1 | ICD10CM | Nontraumatic extradural hemorrhage |  |
| I62.9 | ICD10CM | Nontraumatic intracranial hemorrhage, unspecified |  |
| I71.1 | ICD10CM | Thoracic aortic aneurysm, ruptured |  |
| I71.3 | ICD10CM | Abdominal aortic aneurysm, ruptured |  |
| I71.5 | ICD10CM | Thoracoabdominal aortic aneurysm, ruptured |  |
| I71.8 | ICD10CM | Aortic aneurysm of unspecified site, ruptured |  |
| M25.0 | ICD10CM | Hemarthrosis |  |
| M25.00 | ICD10CM | Hemarthrosis, unspecified joint |  |
| M25.01 | ICD10CM | Hemarthrosis, shoulder |  |
| M25.011 | ICD10CM | Hemarthrosis, right shoulder |  |
| M25.012 | ICD10CM | Hemarthrosis, left shoulder |  |
| M25.019 | ICD10CM | Hemarthrosis, unspecified shoulder |  |
| M25.02 | ICD10CM | Hemarthrosis, elbow |  |
| M25.021 | ICD10CM | Hemarthrosis, right elbow |  |
| M25.022 | ICD10CM | Hemarthrosis, left elbow |  |
| M25.029 | ICD10CM | Hemarthrosis, unspecified elbow |  |
| M25.03 | ICD10CM | Hemarthrosis, wrist |  |
| M25.031 | ICD10CM | Hemarthrosis, right wrist |  |
| M25.032 | ICD10CM | Hemarthrosis, left wrist |  |
| M25.039 | ICD10CM | Hemarthrosis, unspecified wrist |  |
| M25.04 | ICD10CM | Hemarthrosis, hand |  |
| M25.041 | ICD10CM | Hemarthrosis, right hand |  |
| M25.042 | ICD10CM | Hemarthrosis, left hand |  |
| M25.049 | ICD10CM | Hemarthrosis, unspecified hand |  |
| M25.05 | ICD10CM | Hemarthrosis, hip |  |
| M25.051 | ICD10CM | Hemarthrosis, right hip |  |
| M25.052 | ICD10CM | Hemarthrosis, left hip |  |
| M25.059 | ICD10CM | Hemarthrosis, unspecified hip |  |
| M25.06 | ICD10CM | Hemarthrosis, knee |  |
| M25.061 | ICD10CM | Hemarthrosis, right knee |  |
| M25.062 | ICD10CM | Hemarthrosis, left knee |  |
| M25.069 | ICD10CM | Hemarthrosis, unspecified knee |  |
| M25.07 | ICD10CM | Hemarthrosis, ankle and foot |  |
| M25.071 | ICD10CM | Hemarthrosis, right ankle |  |
| M25.072 | ICD10CM | Hemarthrosis, left ankle |  |
| M25.073 | ICD10CM | Hemarthrosis, unspecified ankle |  |
| M25.074 | ICD10CM | Hemarthrosis, right foot |  |
| M25.075 | ICD10CM | Hemarthrosis, left foot |  |
| M25.076 | ICD10CM | Hemarthrosis, unspecified foot |  |
| M25.08 | ICD10CM | Hemarthrosis, other specified site |  |
| M79.A | ICD10CM | Nontraumatic compartment syndrome |  |
| M79.A1 | ICD10CM | Nontraumatic compartment syndrome of upper extremity |  |
| M79.A11 | ICD10CM | Nontraumatic compartment syndrome of right upper extremity |  |
| M79.A12 | ICD10CM | Nontraumatic compartment syndrome of left upper extremity |  |
| M79.A19 | ICD10CM | Nontraumatic compartment syndrome of unspecified upper extremity |  |
| M79.A2 | ICD10CM | Nontraumatic compartment syndrome of lower extremity |  |
| M79.A21 | ICD10CM | Nontraumatic compartment syndrome of right lower extremity |  |
| M79.A22 | ICD10CM | Nontraumatic compartment syndrome of left lower extremity |  |
| M79.A29 | ICD10CM | Nontraumatic compartment syndrome of unspecified lower extremity |  |
| M79.A3 | ICD10CM | Nontraumatic compartment syndrome of abdomen |  |
| M79.A9 | ICD10CM | Nontraumatic compartment syndrome of other sites |  |

# **Supplementary Table S1b: Overt bleed disease codes, ICD-9-CM and ICD-10-CM**

| **Code** | **Code Type** | **Description** | **Trauma Code Flag** |
| --- | --- | --- | --- |
| 800.2 | ICD9CM | Closed fracture of vault of skull with subarachnoid, subdural, and extradural hemorrhage | 1 |
| 800.3 | ICD9CM | Closed fracture of vault of skull with other and unspecified intracranial hemorrhage | 1 |
| 800.7 | ICD9CM | Open fracture of vault of skull with subarachnoid, subdural, and extradural hemorrhage | 1 |
| 800.8 | ICD9CM | Open fracture of vault of skull with other and unspecified intracranial hemorrhage | 1 |
| 801.2 | ICD9CM | Closed fracture of base of skull with subarachnoid, subdural, and extradural hemorrhage | 1 |
| 801.3 | ICD9CM | Closed fracture of base of skull with other and unspecified intracranial hemorrhage | 1 |
| 801.7 | ICD9CM | Open fracture of base of skull with subarachnoid, subdural, and extradural hemorrhage | 1 |
| 801.8 | ICD9CM | Open fracture of base of skull with other and unspecified intracranial hemorrhage | 1 |
| 803.2 | ICD9CM | Other closed skull fracture with subarachnoid, subdural, and extradural hemorrhage | 1 |
| 803.3 | ICD9CM | Closed skull fracture with other and unspecified intracranial hemorrhage | 1 |
| 803.7 | ICD9CM | Other open skull fracture with subarachnoid, subdural, and extradural hemorrhage | 1 |
| 803.8 | ICD9CM | Other open skull fracture with other and unspecified intracranial hemorrhage | 1 |
| 804.2 | ICD9CM | Closed fractures involving skull or face with other bones with subarachnoid, subdural, and extradural hemorrhage | 1 |
| 804.3 | ICD9CM | Closed fractures involving skull or face with other bones, with other and unspecified intracranial hemorrhage | 1 |
| 804.7 | ICD9CM | Open fractures involving skull or face with other bones with subarachnoid, subdural, and extradural hemorrhage | 1 |
| 804.8 | ICD9CM | Open fractures involving skull or face with other bones, with other and unspecified intracranial hemorrhage | 1 |
| 851.0 | ICD9CM | Cortex (cerebral) contusion without mention of open intracranial wound | 1 |
| 851.00 | ICD9CM | Cortex (cerebral) contusion without mention of open intracranial wound, unspecified state of consciousness | 1 |
| 851.01 | ICD9CM | Cortex (cerebral) contusion without mention of open intracranial wound, with no loss of consciousness | 1 |
| 851.02 | ICD9CM | Cortex (cerebral) contusion without mention of open intracranial wound, with brief [less than one hour] loss of consciousness | 1 |
| 851.03 | ICD9CM | Cortex (cerebral) contusion without mention of open intracranial wound, with moderate [1-24 hours] loss of consciousness | 1 |
| 851.04 | ICD9CM | Cortex (cerebral) contusion without mention of open intracranial wound, with prolonged [more than 24 hours] loss of consciousness and return to pre-existing conscious level | 1 |
| 851.05 | ICD9CM | Cortex (cerebral) contusion without mention of open intracranial wound, with prolonged [more than 24 hours] loss of consciousness without return to pre-existing conscious level | 1 |
| 851.06 | ICD9CM | Cortex (cerebral) contusion without mention of open intracranial wound, with loss of consciousness of unspecified duration | 1 |
| 851.09 | ICD9CM | Cortex (cerebral) contusion without mention of open intracranial wound, with concussion, unspecified | 1 |
| 851.1 | ICD9CM | Cortex (cerebral) contusion with open intracranial wound | 1 |
| 851.10 | ICD9CM | Cortex (cerebral) contusion with open intracranial wound, unspecified state of consciousness | 1 |
| 851.11 | ICD9CM | Cortex (cerebral) contusion with open intracranial wound, with no loss of consciousness | 1 |
| 851.12 | ICD9CM | Cortex (cerebral) contusion with open intracranial wound, with brief [less than one hour] loss of consciousness | 1 |
| 851.13 | ICD9CM | Cortex (cerebral) contusion with open intracranial wound, with moderate [1-24 hours] loss of consciousness | 1 |
| 851.14 | ICD9CM | Cortex (cerebral) contusion with open intracranial wound, with prolonged [more than 24 hours] loss of consciousness and return to pre-existing conscious level | 1 |
| 851.15 | ICD9CM | Cortex (cerebral) contusion with open intracranial wound, with prolonged [more than 24 hours] loss of consciousness without return to pre-existing conscious level | 1 |
| 851.16 | ICD9CM | Cortex (cerebral) contusion with open intracranial wound, with loss of consciousness of unspecified duration | 1 |
| 851.19 | ICD9CM | Cortex (cerebral) contusion with open intracranial wound, with concussion, unspecified | 1 |
| 851.4 | ICD9CM | Cerebellar or brain stem contusion without mention of open intracranial wound | 1 |
| 851.40 | ICD9CM | Cerebellar or brain stem contusion without mention of open intracranial wound, unspecified state of consciousness | 1 |
| 851.41 | ICD9CM | Cerebellar or brain stem contusion without mention of open intracranial wound, with no loss of consciousness | 1 |
| 851.42 | ICD9CM | Cerebellar or brain stem contusion without mention of open intracranial wound, with brief [less than one hour] loss of consciousness | 1 |
| 851.43 | ICD9CM | Cerebellar or brain stem contusion without mention of open intracranial wound, with moderate [1-24 hours] loss of consciousness | 1 |
| 851.44 | ICD9CM | Cerebellar or brain stem contusion without mention of open intracranial wound, with prolonged [more than 24 hours] loss consciousness and return to pre-existing conscious level | 1 |
| 851.45 | ICD9CM | Cerebellar or brain stem contusion without mention of open intracranial wound, with prolonged [more than 24 hours] loss of consciousness without return to pre-existing conscious level | 1 |
| 851.46 | ICD9CM | Cerebellar or brain stem contusion without mention of open intracranial wound, with loss of consciousness of unspecified duration | 1 |
| 851.49 | ICD9CM | Cerebellar or brain stem contusion without mention of open intracranial wound, with concussion, unspecified | 1 |
| 851.5 | ICD9CM | Cerebellar or brain stem contusion with open intracranial wound | 1 |
| 851.50 | ICD9CM | Cerebellar or brain stem contusion with open intracranial wound, unspecified state of consciousness | 1 |
| 851.51 | ICD9CM | Cerebellar or brain stem contusion with open intracranial wound, with no loss of consciousness | 1 |
| 851.52 | ICD9CM | Cerebellar or brain stem contusion with open intracranial wound, with brief [less than one hour] loss of consciousness | 1 |
| 851.53 | ICD9CM | Cerebellar or brain stem contusion with open intracranial wound, with moderate [1-24 hours] loss of consciousness | 1 |
| 851.54 | ICD9CM | Cerebellar or brain stem contusion with open intracranial wound, with prolonged [more than 24 hours] loss of consciousness and return to pre-existing conscious level | 1 |
| 851.55 | ICD9CM | Cerebellar or brain stem contusion with open intracranial wound, with prolonged [more than 24 hours] loss of consciousness without return to pre-existing conscious level | 1 |
| 851.56 | ICD9CM | Cerebellar or brain stem contusion with open intracranial wound, with loss of consciousness of unspecified duration | 1 |
| 851.59 | ICD9CM | Cerebellar or brain stem contusion with open intracranial wound, with concussion, unspecified | 1 |
| 851.9 | ICD9CM | Other and unspecified cerebral laceration and contusion, with open intracranial wound | 1 |
| 851.90 | ICD9CM | Other and unspecified cerebral laceration and contusion, with open intracranial wound, unspecified state of consciousness | 1 |
| 851.91 | ICD9CM | Other and unspecified cerebral laceration and contusion, with open intracranial wound, with no loss of consciousness | 1 |
| 851.92 | ICD9CM | Other and unspecified cerebral laceration and contusion, with open intracranial wound, with brief [less than one hour] loss of consciousness | 1 |
| 851.93 | ICD9CM | Other and unspecified cerebral laceration and contusion, with open intracranial wound, with moderate [1-24 hours] loss of consciousness | 1 |
| 851.94 | ICD9CM | Other and unspecified cerebral laceration and contusion, with open intracranial wound, with prolonged [more than 24 hours] loss of consciousness and return to pre-existing conscious level | 1 |
| 851.95 | ICD9CM | Other and unspecified cerebral laceration and contusion, with open intracranial wound, with prolonged [more than 24 hours] loss of consciousness without return to pre-existing conscious level | 1 |
| 851.96 | ICD9CM | Other and unspecified cerebral laceration and contusion, with open intracranial wound, with loss of consciousness of unspecified duration | 1 |
| 851.99 | ICD9CM | Other and unspecified cerebral laceration and contusion, with open intracranial wound, with concussion, unspecified | 1 |
| 852.0 | ICD9CM | Subarachnoid hemorrhage following injury without mention of open intracranial wound | 1 |
| 852.1 | ICD9CM | Subarachnoid hemorrhage following injury with open intracranial wound | 1 |
| 852.2 | ICD9CM | Subdural hemorrhage following injury without mention of open intracranial wound | 1 |
| 852.3 | ICD9CM | Subdural hemorrhage following injury, with open intracranial wound | 1 |
| 852.4 | ICD9CM | Extradural hemorrhage following injury without mention of open intracranial wound | 1 |
| 852.5 | ICD9CM | Extradural hemorrhage following injury with open intracranial wound | 1 |
| 860.2 | ICD9CM | Traumatic hemothorax without mention of open wound into thorax | 1 |
| 860.3 | ICD9CM | Traumatic hemothorax with open wound into thorax | 1 |
| 860.4 | ICD9CM | Traumatic pneumohemothorax without mention of open wound into thorax | 1 |
| 860.5 | ICD9CM | Traumatic pneumohemothorax with open wound into thorax | 1 |
| 864.11 | ICD9CM | Injury to liver with open wound into cavity, hematoma and contusion | 1 |
| 865.11 | ICD9CM | Injury to spleen with open wound into cavity, hematoma without rupture of capsule | 1 |
| 866.11 | ICD9CM | Injury to kidney with open wound into cavity, hematoma without rupture of capsule | 1 |
| 900 | ICD9CM | Injury to blood vessels of head and neck | 1 |
| 900.0 | ICD9CM | Injury to carotid artery | 1 |
| 900.00 | ICD9CM | Injury to carotid artery, unspecified | 1 |
| 900.01 | ICD9CM | Injury to common carotid artery | 1 |
| 900.02 | ICD9CM | Injury to external carotid artery | 1 |
| 900.03 | ICD9CM | Injury to internal carotid artery | 1 |
| 900.1 | ICD9CM | Injury to internal jugular vein | 1 |
| 900.8 | ICD9CM | Injury to other specified blood vessels of head and neck | 1 |
| 900.81 | ICD9CM | Injury to external jugular vein | 1 |
| 900.82 | ICD9CM | Injury to multiple blood vessels of head and neck | 1 |
| 900.89 | ICD9CM | Injury to other specified blood vessels of head and neck | 1 |
| 900.9 | ICD9CM | Injury to unspecified blood vessel of head and neck | 1 |
| 901 | ICD9CM | Injury to blood vessels of thorax | 1 |
| 901.0 | ICD9CM | Injury to thoracic aorta | 1 |
| 901.1 | ICD9CM | Injury to innominate and subclavian arteries | 1 |
| 901.2 | ICD9CM | Injury to superior vena cava | 1 |
| 901.3 | ICD9CM | Injury to innominate and subclavian veins | 1 |
| 901.4 | ICD9CM | Injury to pulmonary blood vessels | 1 |
| 901.40 | ICD9CM | Injury to pulmonary vessel(s), unspecified | 1 |
| 901.41 | ICD9CM | Injury to pulmonary artery | 1 |
| 901.42 | ICD9CM | Injury to pulmonary vein | 1 |
| 901.8 | ICD9CM | Injury to other specified blood vessels of thorax | 1 |
| 901.81 | ICD9CM | Injury to intercostal artery or vein | 1 |
| 901.82 | ICD9CM | Injury to internal mammary artery or vein | 1 |
| 901.83 | ICD9CM | Injury to multiple blood vessels of thorax | 1 |
| 901.89 | ICD9CM | Injury to other specified blood vessels of thorax | 1 |
| 901.9 | ICD9CM | Injury to unspecified blood vessel of thorax | 1 |
| 902 | ICD9CM | Injury to blood vessels of abdomen and pelvis | 1 |
| 902.0 | ICD9CM | Injury to abdominal aorta | 1 |
| 902.1 | ICD9CM | Injury to inferior vena cava | 1 |
| 902.10 | ICD9CM | Injury to inferior vena cava, unspecified | 1 |
| 902.11 | ICD9CM | Injury to hepatic veins | 1 |
| 902.19 | ICD9CM | Injury to inferior vena cava, other | 1 |
| 902.2 | ICD9CM | Injury to celiac and mesenteric arteries | 1 |
| 902.20 | ICD9CM | Injury to celiac and mesenteric arteries, unspecified | 1 |
| 902.21 | ICD9CM | Injury to gastric artery | 1 |
| 902.22 | ICD9CM | Injury to hepatic artery | 1 |
| 902.23 | ICD9CM | Injury to splenic artery | 1 |
| 902.24 | ICD9CM | Injury to other specified branches of celiac axis | 1 |
| 902.25 | ICD9CM | Injury to superior mesenteric artery (trunk) | 1 |
| 902.26 | ICD9CM | Injury to primary branches of superior mesenteric artery | 1 |
| 902.27 | ICD9CM | Injury to inferior mesenteric artery | 1 |
| 902.29 | ICD9CM | Injury to celiac and mesenteric arteries, other | 1 |
| 902.3 | ICD9CM | Injury to portal and splenic veins | 1 |
| 902.31 | ICD9CM | Injury to superior mesenteric vein and primary subdivisions | 1 |
| 902.32 | ICD9CM | Injury to inferior mesenteric vein | 1 |
| 902.33 | ICD9CM | Injury to portal vein | 1 |
| 902.34 | ICD9CM | Injury to splenic vein | 1 |
| 902.39 | ICD9CM | Injury to portal and splenic veins, other | 1 |
| 902.4 | ICD9CM | Injury to renal blood vessels | 1 |
| 902.40 | ICD9CM | Injury to renal vessel(s), unspecified | 1 |
| 902.41 | ICD9CM | Injury to renal artery | 1 |
| 902.42 | ICD9CM | Injury to renal vein | 1 |
| 902.49 | ICD9CM | Injury to renal blood vessels, other | 1 |
| 902.5 | ICD9CM | Injury to iliac blood vessels | 1 |
| 902.50 | ICD9CM | Injury to iliac vessel(s), unspecified | 1 |
| 902.51 | ICD9CM | Injury to hypogastric artery | 1 |
| 902.52 | ICD9CM | Injury to hypogastric vein | 1 |
| 902.53 | ICD9CM | Injury to iliac artery | 1 |
| 902.54 | ICD9CM | Injury to iliac vein | 1 |
| 902.55 | ICD9CM | Injury to uterine artery | 1 |
| 902.56 | ICD9CM | Injury to uterine vein | 1 |
| 902.59 | ICD9CM | Injury to iliac blood vessels, other | 1 |
| 902.8 | ICD9CM | Injury to other specified blood vessels of abdomen and pelvis | 1 |
| 902.81 | ICD9CM | Injury to ovarian artery | 1 |
| 902.82 | ICD9CM | Injury to ovarian vein | 1 |
| 902.87 | ICD9CM | Injury to multiple blood vessels of abdomen and pelvis | 1 |
| 902.89 | ICD9CM | Injury to other specified blood vessels of abdomen and pelvis | 1 |
| 902.9 | ICD9CM | Injury to unspecified blood vessel of abdomen and pelvis | 1 |
| 903 | ICD9CM | Injury to blood vessels of upper extremity | 1 |
| 903.0 | ICD9CM | Injury to axillary blood vessels | 1 |
| 903.00 | ICD9CM | Injury to axillary vessel(s), unspecified | 1 |
| 903.01 | ICD9CM | Injury to axillary artery | 1 |
| 903.02 | ICD9CM | Injury to axillary vein | 1 |
| 903.1 | ICD9CM | Injury to brachial blood vessels | 1 |
| 903.2 | ICD9CM | Injury to radial blood vessels | 1 |
| 903.3 | ICD9CM | Injury to ulnar blood vessels | 1 |
| 903.4 | ICD9CM | Injury to palmar artery | 1 |
| 903.5 | ICD9CM | Injury to digital blood vessels | 1 |
| 903.8 | ICD9CM | Injury to other specified blood vessels of upper extremity | 1 |
| 903.9 | ICD9CM | Injury to unspecified blood vessel of upper extremity | 1 |
| 904 | ICD9CM | Injury to blood vessels of lower extremity and unspecified sites | 1 |
| 904.0 | ICD9CM | Injury to common femoral artery | 1 |
| 904.1 | ICD9CM | Injury to superficial femoral artery | 1 |
| 904.2 | ICD9CM | Injury to femoral veins | 1 |
| 904.3 | ICD9CM | Injury to saphenous veins | 1 |
| 904.4 | ICD9CM | Injury to popliteal blood vessels | 1 |
| 904.40 | ICD9CM | Injury to popliteal vessel(s), unspecified | 1 |
| 904.41 | ICD9CM | Injury to popliteal artery | 1 |
| 904.42 | ICD9CM | Injury to popliteal vein | 1 |
| 904.5 | ICD9CM | Injury to tibial blood vessels | 1 |
| 904.50 | ICD9CM | Injury to tibial vessel(s), unspecified | 1 |
| 904.51 | ICD9CM | Injury to anterior tibial artery | 1 |
| 904.52 | ICD9CM | Injury to anterior tibial vein | 1 |
| 904.53 | ICD9CM | Injury to posterior tibial artery | 1 |
| 904.54 | ICD9CM | Injury to posterior tibial vein | 1 |
| 904.6 | ICD9CM | Injury to deep plantar blood vessels | 1 |
| 904.7 | ICD9CM | Injury to other specified blood vessels of lower extremity | 1 |
| 904.8 | ICD9CM | Injury to unspecified blood vessel of lower extremity | 1 |
| 904.9 | ICD9CM | Injury to blood vessels of unspecified site | 1 |
| 920 | ICD9CM | Contusion of face, scalp, and neck except eye(s) | 1 |
| 920-924.99 | ICD9CM | Contusion with intact skin surface | 1 |
| 921 | ICD9CM | Contusion of eye and adnexa | 1 |
| 921.0 | ICD9CM | Black eye, not otherwise specified | 1 |
| 921.1 | ICD9CM | Contusion of eyelids and periocular area | 1 |
| 921.2 | ICD9CM | Contusion of orbital tissues | 1 |
| 921.3 | ICD9CM | Contusion of eyeball | 1 |
| 921.9 | ICD9CM | Unspecified contusion of eye | 1 |
| 922 | ICD9CM | Contusion of trunk | 1 |
| 922.0 | ICD9CM | Contusion of breast | 1 |
| 922.1 | ICD9CM | Contusion of chest wall | 1 |
| 922.2 | ICD9CM | Contusion of abdominal wall | 1 |
| 922.3 | ICD9CM | Contusion of back | 1 |
| 922.31 | ICD9CM | Contusion of back | 1 |
| 922.32 | ICD9CM | Contusion of buttock | 1 |
| 922.33 | ICD9CM | Contusion of interscapular region | 1 |
| 922.4 | ICD9CM | Contusion of genital organs | 1 |
| 922.8 | ICD9CM | Contusion of multiple sites of trunk | 1 |
| 922.9 | ICD9CM | Contusion of unspecified part of trunk | 1 |
| 923 | ICD9CM | Contusion of upper limb | 1 |
| 923.0 | ICD9CM | Contusion of shoulder and upper arm | 1 |
| 923.00 | ICD9CM | Contusion of shoulder region | 1 |
| 923.01 | ICD9CM | Contusion of scapular region | 1 |
| 923.02 | ICD9CM | Contusion of axillary region | 1 |
| 923.03 | ICD9CM | Contusion of upper arm | 1 |
| 923.09 | ICD9CM | Contusion of multiple sites of shoulder and upper arm | 1 |
| 923.1 | ICD9CM | Contusion of elbow and forearm | 1 |
| 923.10 | ICD9CM | Contusion of forearm | 1 |
| 923.11 | ICD9CM | Contusion of elbow | 1 |
| 923.2 | ICD9CM | Contusion of wrist and hand(s), except finger(s) alone | 1 |
| 923.20 | ICD9CM | Contusion of hand(s) | 1 |
| 923.21 | ICD9CM | Contusion of wrist | 1 |
| 923.3 | ICD9CM | Contusion of finger | 1 |
| 923.8 | ICD9CM | Contusion of multiple sites of upper limb | 1 |
| 923.9 | ICD9CM | Contusion of unspecified part of upper limb | 1 |
| 924 | ICD9CM | Contusion of lower limb and of other and unspecified sites | 1 |
| 924.0 | ICD9CM | Contusion of hip and thigh | 1 |
| 924.00 | ICD9CM | Contusion of thigh | 1 |
| 924.01 | ICD9CM | Contusion of hip | 1 |
| 924.1 | ICD9CM | Contusion of knee and lower leg | 1 |
| 924.10 | ICD9CM | Contusion of lower leg | 1 |
| 924.11 | ICD9CM | Contusion of knee | 1 |
| 924.2 | ICD9CM | Contusion of ankle and foot, excluding toe(s) | 1 |
| 924.20 | ICD9CM | Contusion of foot | 1 |
| 924.21 | ICD9CM | Contusion of ankle | 1 |
| 924.3 | ICD9CM | Contusion of toe | 1 |
| 924.4 | ICD9CM | Contusion of multiple sites of lower limb | 1 |
| 924.5 | ICD9CM | Contusion of unspecified part of lower limb | 1 |
| 924.8 | ICD9CM | Contusion of multiple sites, not elsewhere classified | 1 |
| 924.9 | ICD9CM | Contusion of unspecified site | 1 |
| 958.2 | ICD9CM | Secondary and recurrent hemorrhage | 1 |
| 998.1 | ICD9CM | Hemorrhage or hematoma complicating a procedure | 1 |
| 998.12 | ICD9CM | Hematoma complicating a procedure | 1 |
| D78.0 | ICD10CM | Intraoperative hemorrhage and hematoma of the spleen complicating a procedure | 1 |
| D78.01 | ICD10CM | Intraoperative hemorrhage and hematoma of the spleen complicating a procedure on the spleen | 1 |
| D78.02 | ICD10CM | Intraoperative hemorrhage and hematoma of the spleen complicating other procedure | 1 |
| D78.2 | ICD10CM | Postprocedural hemorrhage of the spleen following a procedure | 1 |
| D78.21 | ICD10CM | Postprocedural hemorrhage of the spleen following a procedure on the spleen | 1 |
| D78.22 | ICD10CM | Postprocedural hemorrhage of the spleen following other procedure | 1 |
| D78.31 | ICD10CM | Postprocedural hematoma of the spleen following a procedure on the spleen | 1 |
| D78.32 | ICD10CM | Postprocedural hematoma of the spleen following other procedure | 1 |
| E36.0 | ICD10CM | Intraoperative hemorrhage and hematoma of an endocrine system organ or structure complicating a procedure | 1 |
| E36.01 | ICD10CM | Intraoperative hemorrhage and hematoma of an endocrine system organ or structure complicating an endocrine system procedure | 1 |
| E36.02 | ICD10CM | Intraoperative hemorrhage and hematoma of an endocrine system organ or structure complicating other procedure | 1 |
| E89.81 | ICD10CM | Postprocedural hemorrhage of an endocrine system organ or structure following a procedure | 1 |
| E89.810 | ICD10CM | Postprocedural hemorrhage of an endocrine system organ or structure following an endocrine system procedure | 1 |
| E89.811 | ICD10CM | Postprocedural hemorrhage of an endocrine system organ or structure following other procedure | 1 |
| E89.820 | ICD10CM | Postprocedural hematoma of an endocrine system organ or structure following an endocrine system procedure | 1 |
| E89.821 | ICD10CM | Postprocedural hematoma of an endocrine system organ or structure following other procedure | 1 |
| G97.3 | ICD10CM | Intraoperative hemorrhage and hematoma of a nervous system organ or structure complicating a procedure | 1 |
| G97.31 | ICD10CM | Intraoperative hemorrhage and hematoma of a nervous system organ or structure complicating a nervous system procedure | 1 |
| G97.32 | ICD10CM | Intraoperative hemorrhage and hematoma of a nervous system organ or structure complicating other procedure | 1 |
| G97.5 | ICD10CM | Postprocedural hemorrhage of a nervous system organ or structure following a procedure | 1 |
| G97.51 | ICD10CM | Postprocedural hemorrhage of a nervous system organ or structure following a nervous system procedure | 1 |
| G97.52 | ICD10CM | Postprocedural hemorrhage of a nervous system organ or structure following other procedure | 1 |
| G97.61 | ICD10CM | Postprocedural hematoma of a nervous system organ or structure following a nervous system procedure | 1 |
| G97.62 | ICD10CM | Postprocedural hematoma of a nervous system organ or structure following other procedure | 1 |
| H59.1 | ICD10CM | Intraoperative hemorrhage and hematoma of eye and adnexa complicating a procedure | 1 |
| H59.11 | ICD10CM | Intraoperative hemorrhage and hematoma of eye and adnexa complicating an ophthalmic procedure | 1 |
| H59.111 | ICD10CM | Intraoperative hemorrhage and hematoma of right eye and adnexa complicating an ophthalmic procedure | 1 |
| H59.112 | ICD10CM | Intraoperative hemorrhage and hematoma of left eye and adnexa complicating an ophthalmic procedure | 1 |
| H59.113 | ICD10CM | Intraoperative hemorrhage and hematoma of eye and adnexa complicating an ophthalmic procedure, bilateral | 1 |
| H59.119 | ICD10CM | Intraoperative hemorrhage and hematoma of unspecified eye and adnexa complicating an ophthalmic procedure | 1 |
| H59.12 | ICD10CM | Intraoperative hemorrhage and hematoma of eye and adnexa complicating other procedure | 1 |
| H59.121 | ICD10CM | Intraoperative hemorrhage and hematoma of right eye and adnexa complicating other procedure | 1 |
| H59.122 | ICD10CM | Intraoperative hemorrhage and hematoma of left eye and adnexa complicating other procedure | 1 |
| H59.123 | ICD10CM | Intraoperative hemorrhage and hematoma of eye and adnexa complicating other procedure, bilateral | 1 |
| H59.129 | ICD10CM | Intraoperative hemorrhage and hematoma of unspecified eye and adnexa complicating other procedure | 1 |
| H59.31 | ICD10CM | Postprocedural hemorrhage of eye and adnexa following an ophthalmic procedure | 1 |
| H59.311 | ICD10CM | Postprocedural hemorrhage of right eye and adnexa following an ophthalmic procedure | 1 |
| H59.312 | ICD10CM | Postprocedural hemorrhage of left eye and adnexa following an ophthalmic procedure | 1 |
| H59.313 | ICD10CM | Postprocedural hemorrhage of eye and adnexa following an ophthalmic procedure, bilateral | 1 |
| H59.319 | ICD10CM | Postprocedural hemorrhage of unspecified eye and adnexa following an ophthalmic procedure | 1 |
| H59.32 | ICD10CM | Postprocedural hemorrhage of eye and adnexa following other procedure | 1 |
| H59.321 | ICD10CM | Postprocedural hemorrhage of right eye and adnexa following other procedure | 1 |
| H59.322 | ICD10CM | Postprocedural hemorrhage of left eye and adnexa following other procedure | 1 |
| H59.323 | ICD10CM | Postprocedural hemorrhage of eye and adnexa following other procedure, bilateral | 1 |
| H59.329 | ICD10CM | Postprocedural hemorrhage of unspecified eye and adnexa following other procedure | 1 |
| H59.33 | ICD10CM | Postprocedural hematoma of eye and adnexa following an ophthalmic procedure | 1 |
| H59.331 | ICD10CM | Postprocedural hematoma of right eye and adnexa following an ophthalmic procedure | 1 |
| H59.332 | ICD10CM | Postprocedural hematoma of left eye and adnexa following an ophthalmic procedure | 1 |
| H59.333 | ICD10CM | Postprocedural hematoma of eye and adnexa following an ophthalmic procedure, bilateral | 1 |
| H59.339 | ICD10CM | Postprocedural hematoma of unspecified eye and adnexa following an ophthalmic procedure | 1 |
| H59.34 | ICD10CM | Postprocedural hematoma of eye and adnexa following other procedure | 1 |
| H59.341 | ICD10CM | Postprocedural hematoma of right eye and adnexa following other procedure | 1 |
| H59.342 | ICD10CM | Postprocedural hematoma of left eye and adnexa following other procedure | 1 |
| H59.343 | ICD10CM | Postprocedural hematoma of eye and adnexa following other procedure, bilateral | 1 |
| H59.349 | ICD10CM | Postprocedural hematoma of unspecified eye and adnexa following other procedure | 1 |
| H95.2 | ICD10CM | Intraoperative hemorrhage and hematoma of ear and mastoid process complicating a procedure | 1 |
| H95.21 | ICD10CM | Intraoperative hemorrhage and hematoma of ear and mastoid process complicating a procedure on the ear and mastoid process | 1 |
| H95.22 | ICD10CM | Intraoperative hemorrhage and hematoma of ear and mastoid process complicating other procedure | 1 |
| H95.4 | ICD10CM | Postprocedural hemorrhage of ear and mastoid process following a procedure | 1 |
| H95.41 | ICD10CM | Postprocedural hemorrhage of ear and mastoid process following a procedure on the ear and mastoid process | 1 |
| H95.42 | ICD10CM | Postprocedural hemorrhage of ear and mastoid process following other procedure | 1 |
| I97.4 | ICD10CM | Intraoperative hemorrhage and hematoma of a circulatory system organ or structure complicating a procedure | 1 |
| I97.41 | ICD10CM | Intraoperative hemorrhage and hematoma of a circulatory system organ or structure complicating a circulatory system procedure | 1 |
| I97.410 | ICD10CM | Intraoperative hemorrhage and hematoma of a circulatory system organ or structure complicating a cardiac catheterization | 1 |
| I97.411 | ICD10CM | Intraoperative hemorrhage and hematoma of a circulatory system organ or structure complicating a cardiac bypass | 1 |
| I97.418 | ICD10CM | Intraoperative hemorrhage and hematoma of a circulatory system organ or structure complicating other circulatory system procedure | 1 |
| I97.42 | ICD10CM | Intraoperative hemorrhage and hematoma of a circulatory system organ or structure complicating other procedure | 1 |
| I97.61 | ICD10CM | Postprocedural hemorrhage of a circulatory system organ or structure following a circulatory system procedure | 1 |
| I97.610 | ICD10CM | Postprocedural hemorrhage of a circulatory system organ or structure following a cardiac catheterization | 1 |
| I97.611 | ICD10CM | Postprocedural hemorrhage of a circulatory system organ or structure following cardiac bypass | 1 |
| I97.618 | ICD10CM | Postprocedural hemorrhage of a circulatory system organ or structure following other circulatory system procedure | 1 |
| I97.620 | ICD10CM | Postprocedural hemorrhage of a circulatory system organ or structure following other procedure | 1 |
| I97.621 | ICD10CM | Postprocedural hematoma of a circulatory system organ or structure following other procedure | 1 |
| I97.63 | ICD10CM | Postprocedural hematoma of a circulatory system organ or structure following a circulatory system procedure | 1 |
| I97.630 | ICD10CM | Postprocedural hematoma of a circulatory system organ or structure following a cardiac catheterization | 1 |
| I97.631 | ICD10CM | Postprocedural hematoma of a circulatory system organ or structure following cardiac bypass | 1 |
| I97.638 | ICD10CM | Postprocedural hematoma of a circulatory system organ or structure following other circulatory system procedure | 1 |
| J95.6 | ICD10CM | Intraoperative hemorrhage and hematoma of a respiratory system organ or structure complicating a procedure | 1 |
| J95.61 | ICD10CM | Intraoperative hemorrhage and hematoma of a respiratory system organ or structure complicating a respiratory system procedure | 1 |
| J95.62 | ICD10CM | Intraoperative hemorrhage and hematoma of a respiratory system organ or structure complicating other procedure | 1 |
| J95.83 | ICD10CM | Postprocedural hemorrhage of a respiratory system organ or structure following a procedure | 1 |
| J95.830 | ICD10CM | Postprocedural hemorrhage of a respiratory system organ or structure following a respiratory system procedure | 1 |
| J95.831 | ICD10CM | Postprocedural hemorrhage of a respiratory system organ or structure following other procedure | 1 |
| J95.861 | ICD10CM | Postprocedural hematoma of a respiratory system organ or structure following other procedure | 1 |
| K91.6 | ICD10CM | Intraoperative hemorrhage and hematoma of a digestive system organ or structure complicating a procedure | 1 |
| K91.61 | ICD10CM | Intraoperative hemorrhage and hematoma of a digestive system organ or structure complicating a digestive system procedure | 1 |
| K91.62 | ICD10CM | Intraoperative hemorrhage and hematoma of a digestive system organ or structure complicating other procedure | 1 |
| K91.84 | ICD10CM | Postprocedural hemorrhage of a digestive system organ or structure following a procedure | 1 |
| K91.840 | ICD10CM | Postprocedural hemorrhage of a digestive system organ or structure following a digestive system procedure | 1 |
| K91.841 | ICD10CM | Postprocedural hemorrhage of a digestive system organ or structure following other procedure | 1 |
| K91.870 | ICD10CM | Postprocedural hematoma of a digestive system organ or structure following a digestive system procedure | 1 |
| K91.871 | ICD10CM | Postprocedural hematoma of a digestive system organ or structure following other procedure | 1 |
| L76.0 | ICD10CM | Intraoperative hemorrhage and hematoma of skin and subcutaneous tissue complicating a procedure | 1 |
| L76.01 | ICD10CM | Intraoperative hemorrhage and hematoma of skin and subcutaneous tissue complicating a dermatologic procedure | 1 |
| L76.02 | ICD10CM | Intraoperative hemorrhage and hematoma of skin and subcutaneous tissue complicating other procedure | 1 |
| L76.2 | ICD10CM | Postprocedural hemorrhage of skin and subcutaneous tissue following a procedure | 1 |
| L76.21 | ICD10CM | Postprocedural hemorrhage of skin and subcutaneous tissue following a dermatologic procedure | 1 |
| L76.22 | ICD10CM | Postprocedural hemorrhage of skin and subcutaneous tissue following other procedure | 1 |
| L76.32 | ICD10CM | Postprocedural hematoma of skin and subcutaneous tissue following other procedure | 1 |
| M96.81 | ICD10CM | Intraoperative hemorrhage and hematoma of a musculoskeletal structure complicating a procedure | 1 |
| M96.810 | ICD10CM | Intraoperative hemorrhage and hematoma of a musculoskeletal structure complicating a musculoskeletal system procedure | 1 |
| M96.811 | ICD10CM | Intraoperative hemorrhage and hematoma of a musculoskeletal structure complicating other procedure | 1 |
| M96.83 | ICD10CM | Postprocedural hemorrhage of a musculoskeletal structure following a procedure | 1 |
| M96.830 | ICD10CM | Postprocedural hemorrhage of a musculoskeletal structure following a musculoskeletal system procedure | 1 |
| M96.831 | ICD10CM | Postprocedural hemorrhage of a musculoskeletal structure following other procedure | 1 |
| M96.840 | ICD10CM | Postprocedural hematoma of a musculoskeletal structure following a musculoskeletal system procedure | 1 |
| M96.841 | ICD10CM | Postprocedural hematoma of a musculoskeletal structure following other procedure | 1 |
| N99.510 | ICD10CM | Cystostomy hemorrhage | 1 |
| N99.520 | ICD10CM | Hemorrhage of incontinent external stoma of urinary tract | 1 |
| N99.530 | ICD10CM | Hemorrhage of continent stoma of urinary tract | 1 |
| N99.61 | ICD10CM | Intraoperative hemorrhage and hematoma of a genitourinary system organ or structure complicating a genitourinary system procedure | 1 |
| N99.62 | ICD10CM | Intraoperative hemorrhage and hematoma of a genitourinary system organ or structure complicating other procedure | 1 |
| N99.820 | ICD10CM | Postprocedural hemorrhage of a genitourinary system organ or structure following a genitourinary system procedure | 1 |
| N99.821 | ICD10CM | Postprocedural hemorrhage of a genitourinary system organ or structure following other procedure | 1 |
| N99.840 | ICD10CM | Postprocedural hematoma of a genitourinary system organ or structure following a genitourinary system procedure | 1 |
| N99.841 | ICD10CM | Postprocedural hematoma of a genitourinary system organ or structure following other procedure | 1 |
| S00.03XA | ICD10CM | Contusion of scalp, initial encounter | 1 |
| S00.10XA | ICD10CM | Contusion of unspecified eyelid and periocular area, initial encounter | 1 |
| S00.11XA | ICD10CM | Contusion of right eyelid and periocular area, initial encounter | 1 |
| S00.12XA | ICD10CM | Contusion of left eyelid and periocular area, initial encounter | 1 |
| S00.33XA | ICD10CM | Contusion of nose, initial encounter | 1 |
| S00.431A | ICD10CM | Contusion of right ear, initial encounter | 1 |
| S00.432A | ICD10CM | Contusion of left ear, initial encounter | 1 |
| S00.439A | ICD10CM | Contusion of unspecified ear, initial encounter | 1 |
| S00.531A | ICD10CM | Contusion of lip, initial encounter | 1 |
| S00.532A | ICD10CM | Contusion of oral cavity, initial encounter | 1 |
| S00.83XA | ICD10CM | Contusion of other part of head, initial encounter | 1 |
| S00.93XA | ICD10CM | Contusion of unspecified part of head, initial encounter | 1 |
| S05.10XA | ICD10CM | Contusion of eyeball and orbital tissues, unspecified eye, initial encounter | 1 |
| S05.11XA | ICD10CM | Contusion of eyeball and orbital tissues, right eye, initial encounter | 1 |
| S05.12XA | ICD10CM | Contusion of eyeball and orbital tissues, left eye, initial encounter | 1 |
| S06.31 | ICD10CM | Contusion and laceration of right cerebrum | 1 |
| S06.310 | ICD10CM | Contusion and laceration of right cerebrum without loss of consciousness | 1 |
| S06.310A | ICD10CM | Contusion and laceration of right cerebrum without loss of consciousness, initial encounter | 1 |
| S06.311 | ICD10CM | Contusion and laceration of right cerebrum with loss of consciousness of 30 minutes or less | 1 |
| S06.311A | ICD10CM | Contusion and laceration of right cerebrum with loss of consciousness of 30 minutes or less, initial encounter | 1 |
| S06.312 | ICD10CM | Contusion and laceration of right cerebrum with loss of consciousness of 31 minutes to 59 minutes | 1 |
| S06.312A | ICD10CM | Contusion and laceration of right cerebrum with loss of consciousness of 31 minutes to 59 minutes, initial encounter | 1 |
| S06.313 | ICD10CM | Contusion and laceration of right cerebrum with loss of consciousness of 1 hour to 5 hours 59 minutes | 1 |
| S06.313A | ICD10CM | Contusion and laceration of right cerebrum with loss of consciousness of 1 hour to 5 hours 59 minutes, initial encounter | 1 |
| S06.314 | ICD10CM | Contusion and laceration of right cerebrum with loss of consciousness of 6 hours to 24 hours | 1 |
| S06.314A | ICD10CM | Contusion and laceration of right cerebrum with loss of consciousness of 6 hours to 24 hours, initial encounter | 1 |
| S06.315 | ICD10CM | Contusion and laceration of right cerebrum with loss of consciousness greater than 24 hours with return to pre-existing conscious level | 1 |
| S06.315A | ICD10CM | Contusion and laceration of right cerebrum with loss of consciousness greater than 24 hours with return to pre-existing conscious level, initial encounter | 1 |
| S06.316 | ICD10CM | Contusion and laceration of right cerebrum with loss of consciousness greater than 24 hours without return to pre-existing conscious level with patient surviving | 1 |
| S06.316A | ICD10CM | Contusion and laceration of right cerebrum with loss of consciousness greater than 24 hours without return to pre-existing conscious level with patient surviving, initial encounter | 1 |
| S06.317 | ICD10CM | Contusion and laceration of right cerebrum with loss of consciousness of any duration with death due to brain injury prior to regaining consciousness | 1 |
| S06.317A | ICD10CM | Contusion and laceration of right cerebrum with loss of consciousness of any duration with death due to brain injury prior to regaining consciousness, initial encounter | 1 |
| S06.318 | ICD10CM | Contusion and laceration of right cerebrum with loss of consciousness of any duration with death due to other cause prior to regaining consciousness | 1 |
| S06.318A | ICD10CM | Contusion and laceration of right cerebrum with loss of consciousness of any duration with death due to other cause prior to regaining consciousness, initial encounter | 1 |
| S06.319 | ICD10CM | Contusion and laceration of right cerebrum with loss of consciousness of unspecified duration | 1 |
| S06.319A | ICD10CM | Contusion and laceration of right cerebrum with loss of consciousness of unspecified duration, initial encounter | 1 |
| S06.32 | ICD10CM | Contusion and laceration of left cerebrum | 1 |
| S06.320 | ICD10CM | Contusion and laceration of left cerebrum without loss of consciousness | 1 |
| S06.320A | ICD10CM | Contusion and laceration of left cerebrum without loss of consciousness, initial encounter | 1 |
| S06.321 | ICD10CM | Contusion and laceration of left cerebrum with loss of consciousness of 30 minutes or less | 1 |
| S06.321A | ICD10CM | Contusion and laceration of left cerebrum with loss of consciousness of 30 minutes or less, initial encounter | 1 |
| S06.322 | ICD10CM | Contusion and laceration of left cerebrum with loss of consciousness of 31 minutes to 59 minutes | 1 |
| S06.322A | ICD10CM | Contusion and laceration of left cerebrum with loss of consciousness of 31 minutes to 59 minutes, initial encounter | 1 |
| S06.323 | ICD10CM | Contusion and laceration of left cerebrum with loss of consciousness of 1 hour to 5 hours 59 minutes | 1 |
| S06.323A | ICD10CM | Contusion and laceration of left cerebrum with loss of consciousness of 1 hour to 5 hours 59 minutes, initial encounter | 1 |
| S06.324 | ICD10CM | Contusion and laceration of left cerebrum with loss of consciousness of 6 hours to 24 hours | 1 |
| S06.324A | ICD10CM | Contusion and laceration of left cerebrum with loss of consciousness of 6 hours to 24 hours, initial encounter | 1 |
| S06.325 | ICD10CM | Contusion and laceration of left cerebrum with loss of consciousness greater than 24 hours with return to pre-existing conscious level | 1 |
| S06.325A | ICD10CM | Contusion and laceration of left cerebrum with loss of consciousness greater than 24 hours with return to pre-existing conscious level, initial encounter | 1 |
| S06.326 | ICD10CM | Contusion and laceration of left cerebrum with loss of consciousness greater than 24 hours without return to pre-existing conscious level with patient surviving | 1 |
| S06.326A | ICD10CM | Contusion and laceration of left cerebrum with loss of consciousness greater than 24 hours without return to pre-existing conscious level with patient surviving, initial encounter | 1 |
| S06.327 | ICD10CM | Contusion and laceration of left cerebrum with loss of consciousness of any duration with death due to brain injury prior to regaining consciousness | 1 |
| S06.327A | ICD10CM | Contusion and laceration of left cerebrum with loss of consciousness of any duration with death due to brain injury prior to regaining consciousness, initial encounter | 1 |
| S06.328 | ICD10CM | Contusion and laceration of left cerebrum with loss of consciousness of any duration with death due to other cause prior to regaining consciousness | 1 |
| S06.328A | ICD10CM | Contusion and laceration of left cerebrum with loss of consciousness of any duration with death due to other cause prior to regaining consciousness, initial encounter | 1 |
| S06.329 | ICD10CM | Contusion and laceration of left cerebrum with loss of consciousness of unspecified duration | 1 |
| S06.329A | ICD10CM | Contusion and laceration of left cerebrum with loss of consciousness of unspecified duration, initial encounter | 1 |
| S06.33 | ICD10CM | Contusion and laceration of cerebrum, unspecified | 1 |
| S06.330 | ICD10CM | Contusion and laceration of cerebrum, unspecified, without loss of consciousness | 1 |
| S06.330A | ICD10CM | Contusion and laceration of cerebrum, unspecified, without loss of consciousness, initial encounter | 1 |
| S06.331 | ICD10CM | Contusion and laceration of cerebrum, unspecified, with loss of consciousness of 30 minutes or less | 1 |
| S06.331A | ICD10CM | Contusion and laceration of cerebrum, unspecified, with loss of consciousness of 30 minutes or less, initial encounter | 1 |
| S06.332 | ICD10CM | Contusion and laceration of cerebrum, unspecified, with loss of consciousness of 31 minutes to 59 minutes | 1 |
| S06.332A | ICD10CM | Contusion and laceration of cerebrum, unspecified, with loss of consciousness of 31 minutes to 59 minutes, initial encounter | 1 |
| S06.333 | ICD10CM | Contusion and laceration of cerebrum, unspecified, with loss of consciousness of 1 hour to 5 hours 59 minutes | 1 |
| S06.333A | ICD10CM | Contusion and laceration of cerebrum, unspecified, with loss of consciousness of 1 hour to 5 hours 59 minutes, initial encounter | 1 |
| S06.334 | ICD10CM | Contusion and laceration of cerebrum, unspecified, with loss of consciousness of 6 hours to 24 hours | 1 |
| S06.334A | ICD10CM | Contusion and laceration of cerebrum, unspecified, with loss of consciousness of 6 hours to 24 hours, initial encounter | 1 |
| S06.335 | ICD10CM | Contusion and laceration of cerebrum, unspecified, with loss of consciousness greater than 24 hours with return to pre-existing conscious level | 1 |
| S06.335A | ICD10CM | Contusion and laceration of cerebrum, unspecified, with loss of consciousness greater than 24 hours with return to pre-existing conscious level, initial encounter | 1 |
| S06.336 | ICD10CM | Contusion and laceration of cerebrum, unspecified, with loss of consciousness greater than 24 hours without return to pre-existing conscious level with patient surviving | 1 |
| S06.336A | ICD10CM | Contusion and laceration of cerebrum, unspecified, with loss of consciousness greater than 24 hours without return to pre-existing conscious level with patient surviving, initial encounter | 1 |
| S06.337 | ICD10CM | Contusion and laceration of cerebrum, unspecified, with loss of consciousness of any duration with death due to brain injury prior to regaining consciousness | 1 |
| S06.337A | ICD10CM | Contusion and laceration of cerebrum, unspecified, with loss of consciousness of any duration with death due to brain injury prior to regaining consciousness, initial encounter | 1 |
| S06.338 | ICD10CM | Contusion and laceration of cerebrum, unspecified, with loss of consciousness of any duration with death due to other cause prior to regaining consciousness | 1 |
| S06.338A | ICD10CM | Contusion and laceration of cerebrum, unspecified, with loss of consciousness of any duration with death due to other cause prior to regaining consciousness, initial encounter | 1 |
| S06.339 | ICD10CM | Contusion and laceration of cerebrum, unspecified, with loss of consciousness of unspecified duration | 1 |
| S06.339A | ICD10CM | Contusion and laceration of cerebrum, unspecified, with loss of consciousness of unspecified duration, initial encounter | 1 |
| S06.37 | ICD10CM | Contusion, laceration, and hemorrhage of cerebellum | 1 |
| S06.370 | ICD10CM | Contusion, laceration, and hemorrhage of cerebellum without loss of consciousness | 1 |
| S06.370A | ICD10CM | Contusion, laceration, and hemorrhage of cerebellum without loss of consciousness, initial encounter | 1 |
| S06.371 | ICD10CM | Contusion, laceration, and hemorrhage of cerebellum with loss of consciousness of 30 minutes or less | 1 |
| S06.371A | ICD10CM | Contusion, laceration, and hemorrhage of cerebellum with loss of consciousness of 30 minutes or less, initial encounter | 1 |
| S06.372 | ICD10CM | Contusion, laceration, and hemorrhage of cerebellum with loss of consciousness of 31 minutes to 59 minutes | 1 |
| S06.372A | ICD10CM | Contusion, laceration, and hemorrhage of cerebellum with loss of consciousness of 31 minutes to 59 minutes, initial encounter | 1 |
| S06.373 | ICD10CM | Contusion, laceration, and hemorrhage of cerebellum with loss of consciousness of 1 hour to 5 hours 59 minutes | 1 |
| S06.373A | ICD10CM | Contusion, laceration, and hemorrhage of cerebellum with loss of consciousness of 1 hour to 5 hours 59 minutes, initial encounter | 1 |
| S06.374 | ICD10CM | Contusion, laceration, and hemorrhage of cerebellum with loss of consciousness of 6 hours to 24 hours | 1 |
| S06.374A | ICD10CM | Contusion, laceration, and hemorrhage of cerebellum with loss of consciousness of 6 hours to 24 hours, initial encounter | 1 |
| S06.375 | ICD10CM | Contusion, laceration, and hemorrhage of cerebellum with loss of consciousness greater than 24 hours with return to pre-existing conscious level | 1 |
| S06.375A | ICD10CM | Contusion, laceration, and hemorrhage of cerebellum with loss of consciousness greater than 24 hours with return to pre-existing conscious level, initial encounter | 1 |
| S06.376 | ICD10CM | Contusion, laceration, and hemorrhage of cerebellum with loss of consciousness greater than 24 hours without return to pre-existing conscious level with patient surviving | 1 |
| S06.376A | ICD10CM | Contusion, laceration, and hemorrhage of cerebellum with loss of consciousness greater than 24 hours without return to pre-existing conscious level with patient surviving, initial encounter | 1 |
| S06.377 | ICD10CM | Contusion, laceration, and hemorrhage of cerebellum with loss of consciousness of any duration with death due to brain injury prior to regaining consciousness | 1 |
| S06.377A | ICD10CM | Contusion, laceration, and hemorrhage of cerebellum with loss of consciousness of any duration with death due to brain injury prior to regaining consciousness, initial encounter | 1 |
| S06.378 | ICD10CM | Contusion, laceration, and hemorrhage of cerebellum with loss of consciousness of any duration with death due to other cause prior to regaining consciousness | 1 |
| S06.378A | ICD10CM | Contusion, laceration, and hemorrhage of cerebellum with loss of consciousness of any duration with death due to other cause prior to regaining consciousness, initial encounter | 1 |
| S06.379 | ICD10CM | Contusion, laceration, and hemorrhage of cerebellum with loss of consciousness of unspecified duration | 1 |
| S06.379A | ICD10CM | Contusion, laceration, and hemorrhage of cerebellum with loss of consciousness of unspecified duration, initial encounter | 1 |
| S06.38 | ICD10CM | Contusion, laceration, and hemorrhage of brainstem | 1 |
| S06.380 | ICD10CM | Contusion, laceration, and hemorrhage of brainstem without loss of consciousness | 1 |
| S06.380A | ICD10CM | Contusion, laceration, and hemorrhage of brainstem without loss of consciousness, initial encounter | 1 |
| S06.381 | ICD10CM | Contusion, laceration, and hemorrhage of brainstem with loss of consciousness of 30 minutes or less | 1 |
| S06.381A | ICD10CM | Contusion, laceration, and hemorrhage of brainstem with loss of consciousness of 30 minutes or less, initial encounter | 1 |
| S06.382 | ICD10CM | Contusion, laceration, and hemorrhage of brainstem with loss of consciousness of 31 minutes to 59 minutes | 1 |
| S06.382A | ICD10CM | Contusion, laceration, and hemorrhage of brainstem with loss of consciousness of 31 minutes to 59 minutes, initial encounter | 1 |
| S06.383 | ICD10CM | Contusion, laceration, and hemorrhage of brainstem with loss of consciousness of 1 hour to 5 hours 59 minutes | 1 |
| S06.383A | ICD10CM | Contusion, laceration, and hemorrhage of brainstem with loss of consciousness of 1 hour to 5 hours 59 minutes, initial encounter | 1 |
| S06.384 | ICD10CM | Contusion, laceration, and hemorrhage of brainstem with loss of consciousness of 6 hours to 24 hours | 1 |
| S06.384A | ICD10CM | Contusion, laceration, and hemorrhage of brainstem with loss of consciousness of 6 hours to 24 hours, initial encounter | 1 |
| S06.385 | ICD10CM | Contusion, laceration, and hemorrhage of brainstem with loss of consciousness greater than 24 hours with return to pre-existing conscious level | 1 |
| S06.385A | ICD10CM | Contusion, laceration, and hemorrhage of brainstem with loss of consciousness greater than 24 hours with return to pre-existing conscious level, initial encounter | 1 |
| S06.386 | ICD10CM | Contusion, laceration, and hemorrhage of brainstem with loss of consciousness greater than 24 hours without return to pre-existing conscious level with patient surviving | 1 |
| S06.386A | ICD10CM | Contusion, laceration, and hemorrhage of brainstem with loss of consciousness greater than 24 hours without return to pre-existing conscious level with patient surviving, initial encounter | 1 |
| S06.387 | ICD10CM | Contusion, laceration, and hemorrhage of brainstem with loss of consciousness of any duration with death due to brain injury prior to regaining consciousness | 1 |
| S06.387A | ICD10CM | Contusion, laceration, and hemorrhage of brainstem with loss of consciousness of any duration with death due to brain injury prior to regaining consciousness, initial encounter | 1 |
| S06.388 | ICD10CM | Contusion, laceration, and hemorrhage of brainstem with loss of consciousness of any duration with death due to other cause prior to regaining consciousness | 1 |
| S06.388A | ICD10CM | Contusion, laceration, and hemorrhage of brainstem with loss of consciousness of any duration with death due to other cause prior to regaining consciousness, initial encounter | 1 |
| S06.389 | ICD10CM | Contusion, laceration, and hemorrhage of brainstem with loss of consciousness of unspecified duration | 1 |
| S06.389A | ICD10CM | Contusion, laceration, and hemorrhage of brainstem with loss of consciousness of unspecified duration, initial encounter | 1 |
| S09.0XXA | ICD10CM | Injury of blood vessels of head, not elsewhere classified, initial encounter | 1 |
| S10.0XXA | ICD10CM | Contusion of throat, initial encounter | 1 |
| S10.83XA | ICD10CM | Contusion of other specified part of neck, initial encounter | 1 |
| S10.93XA | ICD10CM | Contusion of unspecified part of neck, initial encounter | 1 |
| S15.011A | ICD10CM | Minor laceration of right carotid artery, initial encounter | 1 |
| S15.012A | ICD10CM | Minor laceration of left carotid artery, initial encounter | 1 |
| S15.019A | ICD10CM | Minor laceration of unspecified carotid artery, initial encounter | 1 |
| S15.021A | ICD10CM | Major laceration of right carotid artery, initial encounter | 1 |
| S15.022A | ICD10CM | Major laceration of left carotid artery, initial encounter | 1 |
| S15.029A | ICD10CM | Major laceration of unspecified carotid artery, initial encounter | 1 |
| S15.111A | ICD10CM | Minor laceration of right vertebral artery, initial encounter | 1 |
| S15.112A | ICD10CM | Minor laceration of left vertebral artery, initial encounter | 1 |
| S15.119A | ICD10CM | Minor laceration of unspecified vertebral artery, initial encounter | 1 |
| S15.121A | ICD10CM | Major laceration of right vertebral artery, initial encounter | 1 |
| S15.122A | ICD10CM | Major laceration of left vertebral artery, initial encounter | 1 |
| S15.129A | ICD10CM | Major laceration of unspecified vertebral artery, initial encounter | 1 |
| S15.211A | ICD10CM | Minor laceration of right external jugular vein, initial encounter | 1 |
| S15.212A | ICD10CM | Minor laceration of left external jugular vein, initial encounter | 1 |
| S15.219A | ICD10CM | Minor laceration of unspecified external jugular vein, initial encounter | 1 |
| S15.221A | ICD10CM | Major laceration of right external jugular vein, initial encounter | 1 |
| S15.222A | ICD10CM | Major laceration of left external jugular vein, initial encounter | 1 |
| S15.229A | ICD10CM | Major laceration of unspecified external jugular vein, initial encounter | 1 |
| S15.311A | ICD10CM | Minor laceration of right internal jugular vein, initial encounter | 1 |
| S15.312A | ICD10CM | Minor laceration of left internal jugular vein, initial encounter | 1 |
| S15.319A | ICD10CM | Minor laceration of unspecified internal jugular vein, initial encounter | 1 |
| S15.321A | ICD10CM | Major laceration of right internal jugular vein, initial encounter | 1 |
| S15.322A | ICD10CM | Major laceration of left internal jugular vein, initial encounter | 1 |
| S15.329A | ICD10CM | Major laceration of unspecified internal jugular vein, initial encounter | 1 |
| S15.8XXA | ICD10CM | Injury of other specified blood vessels at neck level, initial encounter | 1 |
| S15.9XXA | ICD10CM | Injury of unspecified blood vessel at neck level, initial encounter | 1 |
| S20.00XA | ICD10CM | Contusion of breast, unspecified breast, initial encounter | 1 |
| S20.01XA | ICD10CM | Contusion of right breast, initial encounter | 1 |
| S20.02XA | ICD10CM | Contusion of left breast, initial encounter | 1 |
| S20.20XA | ICD10CM | Contusion of thorax, unspecified, initial encounter | 1 |
| S20.211A | ICD10CM | Contusion of right front wall of thorax, initial encounter | 1 |
| S20.212A | ICD10CM | Contusion of left front wall of thorax, initial encounter | 1 |
| S20.219A | ICD10CM | Contusion of unspecified front wall of thorax, initial encounter | 1 |
| S20.221A | ICD10CM | Contusion of right back wall of thorax, initial encounter | 1 |
| S20.222A | ICD10CM | Contusion of left back wall of thorax, initial encounter | 1 |
| S20.229A | ICD10CM | Contusion of unspecified back wall of thorax, initial encounter | 1 |
| S25.01XA | ICD10CM | Minor laceration of thoracic aorta, initial encounter | 1 |
| S25.02XA | ICD10CM | Major laceration of thoracic aorta, initial encounter | 1 |
| S25.111A | ICD10CM | Minor laceration of right innominate or subclavian artery, initial encounter | 1 |
| S25.112A | ICD10CM | Minor laceration of left innominate or subclavian artery, initial encounter | 1 |
| S25.119A | ICD10CM | Minor laceration of unspecified innominate or subclavian artery, initial encounter | 1 |
| S25.121A | ICD10CM | Major laceration of right innominate or subclavian artery, initial encounter | 1 |
| S25.122A | ICD10CM | Major laceration of left innominate or subclavian artery, initial encounter | 1 |
| S25.129A | ICD10CM | Major laceration of unspecified innominate or subclavian artery, initial encounter | 1 |
| S25.21XA | ICD10CM | Minor laceration of superior vena cava, initial encounter | 1 |
| S25.22XA | ICD10CM | Major laceration of superior vena cava, initial encounter | 1 |
| S25.311A | ICD10CM | Minor laceration of right innominate or subclavian vein, initial encounter | 1 |
| S25.312A | ICD10CM | Minor laceration of left innominate or subclavian vein, initial encounter | 1 |
| S25.319A | ICD10CM | Minor laceration of unspecified innominate or subclavian vein, initial encounter | 1 |
| S25.321A | ICD10CM | Major laceration of right innominate or subclavian vein, initial encounter | 1 |
| S25.322A | ICD10CM | Major laceration of left innominate or subclavian vein, initial encounter | 1 |
| S25.329A | ICD10CM | Major laceration of unspecified innominate or subclavian vein, initial encounter | 1 |
| S25.411A | ICD10CM | Minor laceration of right pulmonary blood vessels, initial encounter | 1 |
| S25.412A | ICD10CM | Minor laceration of left pulmonary blood vessels, initial encounter | 1 |
| S25.419A | ICD10CM | Minor laceration of unspecified pulmonary blood vessels, initial encounter | 1 |
| S25.421A | ICD10CM | Major laceration of right pulmonary blood vessels, initial encounter | 1 |
| S25.422A | ICD10CM | Major laceration of left pulmonary blood vessels, initial encounter | 1 |
| S25.429A | ICD10CM | Major laceration of unspecified pulmonary blood vessels, initial encounter | 1 |
| S25.511A | ICD10CM | Laceration of intercostal blood vessels, right side, initial encounter | 1 |
| S25.512A | ICD10CM | Laceration of intercostal blood vessels, left side, initial encounter | 1 |
| S25.519A | ICD10CM | Laceration of intercostal blood vessels, unspecified side, initial encounter | 1 |
| S25.811A | ICD10CM | Laceration of other blood vessels of thorax, right side, initial encounter | 1 |
| S25.812A | ICD10CM | Laceration of other blood vessels of thorax, left side, initial encounter | 1 |
| S25.819A | ICD10CM | Laceration of other blood vessels of thorax, unspecified side, initial encounter | 1 |
| S25.91XA | ICD10CM | Laceration of unspecified blood vessel of thorax, initial encounter | 1 |
| S27.1 | ICD10CM | Traumatic hemothorax | 1 |
| S27.1XXA | ICD10CM | Traumatic hemothorax, initial encounter | 1 |
| S27.2 | ICD10CM | Traumatic hemopneumothorax | 1 |
| S27.2XXA | ICD10CM | Traumatic hemopneumothorax, initial encounter | 1 |
| S30.0XXA | ICD10CM | Contusion of lower back and pelvis, initial encounter | 1 |
| S30.1XXA | ICD10CM | Contusion of abdominal wall, initial encounter | 1 |
| S30.201A | ICD10CM | Contusion of unspecified external genital organ, male, initial encounter | 1 |
| S30.202A | ICD10CM | Contusion of unspecified external genital organ, female, initial encounter | 1 |
| S30.21XA | ICD10CM | Contusion of penis, initial encounter | 1 |
| S30.22XA | ICD10CM | Contusion of scrotum and testes, initial encounter | 1 |
| S30.23XA | ICD10CM | Contusion of vagina and vulva, initial encounter | 1 |
| S30.3XXA | ICD10CM | Contusion of anus, initial encounter | 1 |
| S35.01XA | ICD10CM | Minor laceration of abdominal aorta, initial encounter | 1 |
| S35.02XA | ICD10CM | Major laceration of abdominal aorta, initial encounter | 1 |
| S35.09XA | ICD10CM | Other injury of abdominal aorta, initial encounter | 1 |
| S35.11XA | ICD10CM | Minor laceration of inferior vena cava, initial encounter | 1 |
| S35.12XA | ICD10CM | Major laceration of inferior vena cava, initial encounter | 1 |
| S35.19XA | ICD10CM | Other injury of inferior vena cava, initial encounter | 1 |
| S35.211A | ICD10CM | Minor laceration of celiac artery, initial encounter | 1 |
| S35.212A | ICD10CM | Major laceration of celiac artery, initial encounter | 1 |
| S35.218A | ICD10CM | Other injury of celiac artery, initial encounter | 1 |
| S35.221A | ICD10CM | Minor laceration of superior mesenteric artery, initial encounter | 1 |
| S35.222A | ICD10CM | Major laceration of superior mesenteric artery, initial encounter | 1 |
| S35.228A | ICD10CM | Other injury of superior mesenteric artery, initial encounter | 1 |
| S35.231A | ICD10CM | Minor laceration of inferior mesenteric artery, initial encounter | 1 |
| S35.232A | ICD10CM | Major laceration of inferior mesenteric artery, initial encounter | 1 |
| S35.238A | ICD10CM | Other injury of inferior mesenteric artery, initial encounter | 1 |
| S35.291A | ICD10CM | Minor laceration of branches of celiac and mesenteric artery, initial encounter | 1 |
| S35.292A | ICD10CM | Major laceration of branches of celiac and mesenteric artery, initial encounter | 1 |
| S35.298A | ICD10CM | Other injury of branches of celiac and mesenteric artery, initial encounter | 1 |
| S35.311A | ICD10CM | Laceration of portal vein, initial encounter | 1 |
| S35.321A | ICD10CM | Laceration of splenic vein, initial encounter | 1 |
| S35.331A | ICD10CM | Laceration of superior mesenteric vein, initial encounter | 1 |
| S35.341A | ICD10CM | Laceration of inferior mesenteric vein, initial encounter | 1 |
| S35.411A | ICD10CM | Laceration of right renal artery, initial encounter | 1 |
| S35.412A | ICD10CM | Laceration of left renal artery, initial encounter | 1 |
| S35.413A | ICD10CM | Laceration of unspecified renal artery, initial encounter | 1 |
| S35.414A | ICD10CM | Laceration of right renal vein, initial encounter | 1 |
| S35.415A | ICD10CM | Laceration of left renal vein, initial encounter | 1 |
| S35.416A | ICD10CM | Laceration of unspecified renal vein, initial encounter | 1 |
| S35.50XA | ICD10CM | Injury of unspecified iliac blood vessel(s), initial encounter | 1 |
| S35.511A | ICD10CM | Injury of right iliac artery, initial encounter | 1 |
| S35.512A | ICD10CM | Injury of left iliac artery, initial encounter | 1 |
| S35.513A | ICD10CM | Injury of unspecified iliac artery, initial encounter | 1 |
| S35.514A | ICD10CM | Injury of right iliac vein, initial encounter | 1 |
| S35.515A | ICD10CM | Injury of left iliac vein, initial encounter | 1 |
| S35.516A | ICD10CM | Injury of unspecified iliac vein, initial encounter | 1 |
| S35.531A | ICD10CM | Injury of right uterine artery, initial encounter | 1 |
| S35.532A | ICD10CM | Injury of left uterine artery, initial encounter | 1 |
| S35.533A | ICD10CM | Injury of unspecified uterine artery, initial encounter | 1 |
| S35.534A | ICD10CM | Injury of right uterine vein, initial encounter | 1 |
| S35.535A | ICD10CM | Injury of left uterine vein, initial encounter | 1 |
| S35.536A | ICD10CM | Injury of unspecified uterine vein, initial encounter | 1 |
| S35.59XA | ICD10CM | Injury of other iliac blood vessels, initial encounter | 1 |
| S35.8X1A | ICD10CM | Laceration of other blood vessels at abdomen, lower back and pelvis level, initial encounter | 1 |
| S35.91XA | ICD10CM | Laceration of unspecified blood vessel at abdomen, lower back and pelvis level, initial encounter | 1 |
| S36.02 | ICD10CM | Contusion of spleen | 1 |
| S36.020 | ICD10CM | Minor contusion of spleen | 1 |
| S36.020A | ICD10CM | Minor contusion of spleen, initial encounter | 1 |
| S36.021 | ICD10CM | Major contusion of spleen | 1 |
| S36.021A | ICD10CM | Major contusion of spleen, initial encounter | 1 |
| S36.029 | ICD10CM | Unspecified contusion of spleen | 1 |
| S36.029A | ICD10CM | Unspecified contusion of spleen, initial encounter | 1 |
| S36.112 | ICD10CM | Contusion of liver | 1 |
| S36.112A | ICD10CM | Contusion of liver, initial encounter | 1 |
| S36.122 | ICD10CM | Contusion of gallbladder | 1 |
| S36.122A | ICD10CM | Contusion of gallbladder, initial encounter | 1 |
| S36.22 | ICD10CM | Contusion of pancreas | 1 |
| S36.220 | ICD10CM | Contusion of head of pancreas | 1 |
| S36.220A | ICD10CM | Contusion of head of pancreas, initial encounter | 1 |
| S36.221 | ICD10CM | Contusion of body of pancreas | 1 |
| S36.221A | ICD10CM | Contusion of body of pancreas, initial encounter | 1 |
| S36.222 | ICD10CM | Contusion of tail of pancreas | 1 |
| S36.222A | ICD10CM | Contusion of tail of pancreas, initial encounter | 1 |
| S36.229 | ICD10CM | Contusion of unspecified part of pancreas | 1 |
| S36.229A | ICD10CM | Contusion of unspecified part of pancreas, initial encounter | 1 |
| S36.32XA | ICD10CM | Contusion of stomach, initial encounter | 1 |
| S36.42 | ICD10CM | Contusion of small intestine | 1 |
| S36.420 | ICD10CM | Contusion of duodenum | 1 |
| S36.420A | ICD10CM | Contusion of duodenum, initial encounter | 1 |
| S36.428 | ICD10CM | Contusion of other part of small intestine | 1 |
| S36.428A | ICD10CM | Contusion of other part of small intestine, initial encounter | 1 |
| S36.429 | ICD10CM | Contusion of unspecified part of small intestine | 1 |
| S36.429A | ICD10CM | Contusion of unspecified part of small intestine, initial encounter | 1 |
| S36.52 | ICD10CM | Contusion of colon | 1 |
| S36.520 | ICD10CM | Contusion of ascending [right] colon | 1 |
| S36.520A | ICD10CM | Contusion of ascending [right] colon, initial encounter | 1 |
| S36.521 | ICD10CM | Contusion of transverse colon | 1 |
| S36.521A | ICD10CM | Contusion of transverse colon, initial encounter | 1 |
| S36.522 | ICD10CM | Contusion of descending [left] colon | 1 |
| S36.522A | ICD10CM | Contusion of descending [left] colon, initial encounter | 1 |
| S36.523 | ICD10CM | Contusion of sigmoid colon | 1 |
| S36.523A | ICD10CM | Contusion of sigmoid colon, initial encounter | 1 |
| S36.528 | ICD10CM | Contusion of other part of colon | 1 |
| S36.528A | ICD10CM | Contusion of other part of colon, initial encounter | 1 |
| S36.529 | ICD10CM | Contusion of unspecified part of colon | 1 |
| S36.529A | ICD10CM | Contusion of unspecified part of colon, initial encounter | 1 |
| S36.62 | ICD10CM | Contusion of rectum | 1 |
| S36.62XA | ICD10CM | Contusion of rectum, initial encounter | 1 |
| S36.892 | ICD10CM | Contusion of other intra-abdominal organs | 1 |
| S36.892A | ICD10CM | Contusion of other intra-abdominal organs, initial encounter | 1 |
| S36.92 | ICD10CM | Contusion of unspecified intra-abdominal organ | 1 |
| S36.92XA | ICD10CM | Contusion of unspecified intra-abdominal organ, initial encounter | 1 |
| S37.01 | ICD10CM | Minor contusion of kidney | 1 |
| S37.011 | ICD10CM | Minor contusion of right kidney | 1 |
| S37.011A | ICD10CM | Minor contusion of right kidney, initial encounter | 1 |
| S37.012 | ICD10CM | Minor contusion of left kidney | 1 |
| S37.012A | ICD10CM | Minor contusion of left kidney, initial encounter | 1 |
| S37.019 | ICD10CM | Minor contusion of unspecified kidney | 1 |
| S37.019A | ICD10CM | Minor contusion of unspecified kidney, initial encounter | 1 |
| S37.02 | ICD10CM | Major contusion of kidney | 1 |
| S37.021 | ICD10CM | Major contusion of right kidney | 1 |
| S37.021A | ICD10CM | Major contusion of right kidney, initial encounter | 1 |
| S37.022 | ICD10CM | Major contusion of left kidney | 1 |
| S37.022A | ICD10CM | Major contusion of left kidney, initial encounter | 1 |
| S37.029 | ICD10CM | Major contusion of unspecified kidney | 1 |
| S37.029A | ICD10CM | Major contusion of unspecified kidney, initial encounter | 1 |
| S37.12 | ICD10CM | Contusion of ureter | 1 |
| S37.12XA | ICD10CM | Contusion of ureter, initial encounter | 1 |
| S37.22 | ICD10CM | Contusion of bladder | 1 |
| S37.22XA | ICD10CM | Contusion of bladder, initial encounter | 1 |
| S37.32 | ICD10CM | Contusion of urethra | 1 |
| S37.32XA | ICD10CM | Contusion of urethra, initial encounter | 1 |
| S37.42 | ICD10CM | Contusion of ovary | 1 |
| S37.421 | ICD10CM | Contusion of ovary, unilateral | 1 |
| S37.421A | ICD10CM | Contusion of ovary, unilateral, initial encounter | 1 |
| S37.422 | ICD10CM | Contusion of ovary, bilateral | 1 |
| S37.422A | ICD10CM | Contusion of ovary, bilateral, initial encounter | 1 |
| S37.429 | ICD10CM | Contusion of ovary, unspecified | 1 |
| S37.429A | ICD10CM | Contusion of ovary, unspecified, initial encounter | 1 |
| S37.52 | ICD10CM | Contusion of fallopian tube | 1 |
| S37.521 | ICD10CM | Contusion of fallopian tube, unilateral | 1 |
| S37.521A | ICD10CM | Contusion of fallopian tube, unilateral, initial encounter | 1 |
| S37.522 | ICD10CM | Contusion of fallopian tube, bilateral | 1 |
| S37.522A | ICD10CM | Contusion of fallopian tube, bilateral, initial encounter | 1 |
| S37.529 | ICD10CM | Contusion of fallopian tube, unspecified | 1 |
| S37.529A | ICD10CM | Contusion of fallopian tube, unspecified, initial encounter | 1 |
| S37.62 | ICD10CM | Contusion of uterus | 1 |
| S37.62XA | ICD10CM | Contusion of uterus, initial encounter | 1 |
| S37.812 | ICD10CM | Contusion of adrenal gland | 1 |
| S37.812A | ICD10CM | Contusion of adrenal gland, initial encounter | 1 |
| S37.822 | ICD10CM | Contusion of prostate | 1 |
| S37.822A | ICD10CM | Contusion of prostate, initial encounter | 1 |
| S37.892 | ICD10CM | Contusion of other urinary and pelvic organ | 1 |
| S37.892A | ICD10CM | Contusion of other urinary and pelvic organ, initial encounter | 1 |
| S37.92 | ICD10CM | Contusion of unspecified urinary and pelvic organ | 1 |
| S37.92XA | ICD10CM | Contusion of unspecified urinary and pelvic organ, initial encounter | 1 |
| S40.011A | ICD10CM | Contusion of right shoulder, initial encounter | 1 |
| S40.012A | ICD10CM | Contusion of left shoulder, initial encounter | 1 |
| S40.019A | ICD10CM | Contusion of unspecified shoulder, initial encounter | 1 |
| S40.021A | ICD10CM | Contusion of right upper arm, initial encounter | 1 |
| S40.022A | ICD10CM | Contusion of left upper arm, initial encounter | 1 |
| S40.029A | ICD10CM | Contusion of unspecified upper arm, initial encounter | 1 |
| S45.011A | ICD10CM | Laceration of axillary artery, right side, initial encounter | 1 |
| S45.012A | ICD10CM | Laceration of axillary artery, left side, initial encounter | 1 |
| S45.019A | ICD10CM | Laceration of axillary artery, unspecified side, initial encounter | 1 |
| S45.111A | ICD10CM | Laceration of brachial artery, right side, initial encounter | 1 |
| S45.112A | ICD10CM | Laceration of brachial artery, left side, initial encounter | 1 |
| S45.119A | ICD10CM | Laceration of brachial artery, unspecified side, initial encounter | 1 |
| S45.211A | ICD10CM | Laceration of axillary or brachial vein, right side, initial encounter | 1 |
| S45.212A | ICD10CM | Laceration of axillary or brachial vein, left side, initial encounter | 1 |
| S45.219A | ICD10CM | Laceration of axillary or brachial vein, unspecified side, initial encounter | 1 |
| S45.311A | ICD10CM | Laceration of superficial vein at shoulder and upper arm level, right arm, initial encounter | 1 |
| S45.312A | ICD10CM | Laceration of superficial vein at shoulder and upper arm level, left arm, initial encounter | 1 |
| S45.319A | ICD10CM | Laceration of superficial vein at shoulder and upper arm level, unspecified arm, initial encounter | 1 |
| S45.811A | ICD10CM | Laceration of other specified blood vessels at shoulder and upper arm level, right arm, initial encounter | 1 |
| S45.812A | ICD10CM | Laceration of other specified blood vessels at shoulder and upper arm level, left arm, initial encounter | 1 |
| S45.819A | ICD10CM | Laceration of other specified blood vessels at shoulder and upper arm level, unspecified arm, initial encounter | 1 |
| S45.911A | ICD10CM | Laceration of unspecified blood vessel at shoulder and upper arm level, right arm, initial encounter | 1 |
| S45.912A | ICD10CM | Laceration of unspecified blood vessel at shoulder and upper arm level, left arm, initial encounter | 1 |
| S45.919A | ICD10CM | Laceration of unspecified blood vessel at shoulder and upper arm level, unspecified arm, initial encounter | 1 |
| S50.00XA | ICD10CM | Contusion of unspecified elbow, initial encounter | 1 |
| S50.01XA | ICD10CM | Contusion of right elbow, initial encounter | 1 |
| S50.02XA | ICD10CM | Contusion of left elbow, initial encounter | 1 |
| S50.10XA | ICD10CM | Contusion of unspecified forearm, initial encounter | 1 |
| S50.11XA | ICD10CM | Contusion of right forearm, initial encounter | 1 |
| S50.12XA | ICD10CM | Contusion of left forearm, initial encounter | 1 |
| S55.011A | ICD10CM | Laceration of ulnar artery at forearm level, right arm, initial encounter | 1 |
| S55.012A | ICD10CM | Laceration of ulnar artery at forearm level, left arm, initial encounter | 1 |
| S55.019A | ICD10CM | Laceration of ulnar artery at forearm level, unspecified arm, initial encounter | 1 |
| S55.111A | ICD10CM | Laceration of radial artery at forearm level, right arm, initial encounter | 1 |
| S55.112A | ICD10CM | Laceration of radial artery at forearm level, left arm, initial encounter | 1 |
| S55.119A | ICD10CM | Laceration of radial artery at forearm level, unspecified arm, initial encounter | 1 |
| S55.211A | ICD10CM | Laceration of vein at forearm level, right arm, initial encounter | 1 |
| S55.212A | ICD10CM | Laceration of vein at forearm level, left arm, initial encounter | 1 |
| S55.219A | ICD10CM | Laceration of vein at forearm level, unspecified arm, initial encounter | 1 |
| S55.811A | ICD10CM | Laceration of other blood vessels at forearm level, right arm, initial encounter | 1 |
| S55.812A | ICD10CM | Laceration of other blood vessels at forearm level, left arm, initial encounter | 1 |
| S55.819A | ICD10CM | Laceration of other blood vessels at forearm level, unspecified arm, initial encounter | 1 |
| S55.911A | ICD10CM | Laceration of unspecified blood vessel at forearm level, right arm, initial encounter | 1 |
| S55.912A | ICD10CM | Laceration of unspecified blood vessel at forearm level, left arm, initial encounter | 1 |
| S55.919A | ICD10CM | Laceration of unspecified blood vessel at forearm level, unspecified arm, initial encounter | 1 |
| S60.00XA | ICD10CM | Contusion of unspecified finger without damage to nail, initial encounter | 1 |
| S60.011A | ICD10CM | Contusion of right thumb without damage to nail, initial encounter | 1 |
| S60.012A | ICD10CM | Contusion of left thumb without damage to nail, initial encounter | 1 |
| S60.019A | ICD10CM | Contusion of unspecified thumb without damage to nail, initial encounter | 1 |
| S60.021A | ICD10CM | Contusion of right index finger without damage to nail, initial encounter | 1 |
| S60.022A | ICD10CM | Contusion of left index finger without damage to nail, initial encounter | 1 |
| S60.029A | ICD10CM | Contusion of unspecified index finger without damage to nail, initial encounter | 1 |
| S60.031A | ICD10CM | Contusion of right middle finger without damage to nail, initial encounter | 1 |
| S60.032A | ICD10CM | Contusion of left middle finger without damage to nail, initial encounter | 1 |
| S60.039A | ICD10CM | Contusion of unspecified middle finger without damage to nail, initial encounter | 1 |
| S60.041A | ICD10CM | Contusion of right ring finger without damage to nail, initial encounter | 1 |
| S60.042A | ICD10CM | Contusion of left ring finger without damage to nail, initial encounter | 1 |
| S60.049A | ICD10CM | Contusion of unspecified ring finger without damage to nail, initial encounter | 1 |
| S60.051A | ICD10CM | Contusion of right little finger without damage to nail, initial encounter | 1 |
| S60.052A | ICD10CM | Contusion of left little finger without damage to nail, initial encounter | 1 |
| S60.059A | ICD10CM | Contusion of unspecified little finger without damage to nail, initial encounter | 1 |
| S60.10XA | ICD10CM | Contusion of unspecified finger with damage to nail, initial encounter | 1 |
| S60.111A | ICD10CM | Contusion of right thumb with damage to nail, initial encounter | 1 |
| S60.112A | ICD10CM | Contusion of left thumb with damage to nail, initial encounter | 1 |
| S60.119A | ICD10CM | Contusion of unspecified thumb with damage to nail, initial encounter | 1 |
| S60.121A | ICD10CM | Contusion of right index finger with damage to nail, initial encounter | 1 |
| S60.122A | ICD10CM | Contusion of left index finger with damage to nail, initial encounter | 1 |
| S60.129A | ICD10CM | Contusion of unspecified index finger with damage to nail, initial encounter | 1 |
| S60.131A | ICD10CM | Contusion of right middle finger with damage to nail, initial encounter | 1 |
| S60.132A | ICD10CM | Contusion of left middle finger with damage to nail, initial encounter | 1 |
| S60.139A | ICD10CM | Contusion of unspecified middle finger with damage to nail, initial encounter | 1 |
| S60.141A | ICD10CM | Contusion of right ring finger with damage to nail, initial encounter | 1 |
| S60.142A | ICD10CM | Contusion of left ring finger with damage to nail, initial encounter | 1 |
| S60.149A | ICD10CM | Contusion of unspecified ring finger with damage to nail, initial encounter | 1 |
| S60.151A | ICD10CM | Contusion of right little finger with damage to nail, initial encounter | 1 |
| S60.152A | ICD10CM | Contusion of left little finger with damage to nail, initial encounter | 1 |
| S60.159A | ICD10CM | Contusion of unspecified little finger with damage to nail, initial encounter | 1 |
| S60.211A | ICD10CM | Contusion of right wrist, initial encounter | 1 |
| S60.212A | ICD10CM | Contusion of left wrist, initial encounter | 1 |
| S60.219A | ICD10CM | Contusion of unspecified wrist, initial encounter | 1 |
| S60.221A | ICD10CM | Contusion of right hand, initial encounter | 1 |
| S60.222A | ICD10CM | Contusion of left hand, initial encounter | 1 |
| S60.229A | ICD10CM | Contusion of unspecified hand, initial encounter | 1 |
| S65.011A | ICD10CM | Laceration of ulnar artery at wrist and hand level of right arm, initial encounter | 1 |
| S65.012A | ICD10CM | Laceration of ulnar artery at wrist and hand level of left arm, initial encounter | 1 |
| S65.019A | ICD10CM | Laceration of ulnar artery at wrist and hand level of unspecified arm, initial encounter | 1 |
| S65.111A | ICD10CM | Laceration of radial artery at wrist and hand level of right arm, initial encounter | 1 |
| S65.112A | ICD10CM | Laceration of radial artery at wrist and hand level of left arm, initial encounter | 1 |
| S65.119A | ICD10CM | Laceration of radial artery at wrist and hand level of unspecified arm, initial encounter | 1 |
| S65.211A | ICD10CM | Laceration of superficial palmar arch of right hand, initial encounter | 1 |
| S65.212A | ICD10CM | Laceration of superficial palmar arch of left hand, initial encounter |  |
| S65.219A | ICD10CM | Laceration of superficial palmar arch of unspecified hand, initial encounter | 1 |
| S65.311A | ICD10CM | Laceration of deep palmar arch of right hand, initial encounter | 1 |
| S65.312A | ICD10CM | Laceration of deep palmar arch of left hand, initial encounter | 1 |
| S65.319A | ICD10CM | Laceration of deep palmar arch of unspecified hand, initial encounter | 1 |
| S65.411A | ICD10CM | Laceration of blood vessel of right thumb, initial encounter | 1 |
| S65.412A | ICD10CM | Laceration of blood vessel of left thumb, initial encounter | 1 |
| S65.419A | ICD10CM | Laceration of blood vessel of unspecified thumb, initial encounter | 1 |
| S65.510A | ICD10CM | Laceration of blood vessel of right index finger, initial encounter | 1 |
| S65.511A | ICD10CM | Laceration of blood vessel of left index finger, initial encounter | 1 |
| S65.512A | ICD10CM | Laceration of blood vessel of right middle finger, initial encounter | 1 |
| S65.513A | ICD10CM | Laceration of blood vessel of left middle finger, initial encounter | 1 |
| S65.514A | ICD10CM | Laceration of blood vessel of right ring finger, initial encounter | 1 |
| S65.515A | ICD10CM | Laceration of blood vessel of left ring finger, initial encounter | 1 |
| S65.516A | ICD10CM | Laceration of blood vessel of right little finger, initial encounter | 1 |
| S65.517A | ICD10CM | Laceration of blood vessel of left little finger, initial encounter | 1 |
| S65.518A | ICD10CM | Laceration of blood vessel of other finger, initial encounter | 1 |
| S65.519A | ICD10CM | Laceration of blood vessel of unspecified finger, initial encounter | 1 |
| S65.811A | ICD10CM | Laceration of other blood vessels at wrist and hand level of right arm, initial encounter | 1 |
| S65.812A | ICD10CM | Laceration of other blood vessels at wrist and hand level of left arm, initial encounter | 1 |
| S65.819A | ICD10CM | Laceration of other blood vessels at wrist and hand level of unspecified arm, initial encounter | 1 |
| S65.911A | ICD10CM | Laceration of unspecified blood vessel at wrist and hand level of right arm, initial encounter | 1 |
| S65.912A | ICD10CM | Laceration of unspecified blood vessel at wrist and hand level of left arm, initial encounter | 1 |
| S65.919A | ICD10CM | Laceration of unspecified blood vessel at wrist and hand level of unspecified arm, initial encounter | 1 |
| S70.00XA | ICD10CM | Contusion of unspecified hip, initial encounter | 1 |
| S70.01XA | ICD10CM | Contusion of right hip, initial encounter | 1 |
| S70.02XA | ICD10CM | Contusion of left hip, initial encounter | 1 |
| S70.10XA | ICD10CM | Contusion of unspecified thigh, initial encounter | 1 |
| S70.11XA | ICD10CM | Contusion of right thigh, initial encounter | 1 |
| S70.12XA | ICD10CM | Contusion of left thigh, initial encounter | 1 |
| S75.011A | ICD10CM | Minor laceration of femoral artery, right leg, initial encounter | 1 |
| S75.012A | ICD10CM | Minor laceration of femoral artery, left leg, initial encounter | 1 |
| S75.019A | ICD10CM | Minor laceration of femoral artery, unspecified leg, initial encounter | 1 |
| S75.021A | ICD10CM | Major laceration of femoral artery, right leg, initial encounter | 1 |
| S75.022A | ICD10CM | Major laceration of femoral artery, left leg, initial encounter | 1 |
| S75.029A | ICD10CM | Major laceration of femoral artery, unspecified leg, initial encounter | 1 |
| S75.111A | ICD10CM | Minor laceration of femoral vein at hip and thigh level, right leg, initial encounter | 1 |
| S75.112A | ICD10CM | Minor laceration of femoral vein at hip and thigh level, left leg, initial encounter | 1 |
| S75.119A | ICD10CM | Minor laceration of femoral vein at hip and thigh level, unspecified leg, initial encounter | 1 |
| S75.121A | ICD10CM | Major laceration of femoral vein at hip and thigh level, right leg, initial encounter | 1 |
| S75.122A | ICD10CM | Major laceration of femoral vein at hip and thigh level, left leg, initial encounter | 1 |
| S75.129A | ICD10CM | Major laceration of femoral vein at hip and thigh level, unspecified leg, initial encounter | 1 |
| S75.211A | ICD10CM | Minor laceration of greater saphenous vein at hip and thigh level, right leg, initial encounter | 1 |
| S75.212A | ICD10CM | Minor laceration of greater saphenous vein at hip and thigh level, left leg, initial encounter | 1 |
| S75.219A | ICD10CM | Minor laceration of greater saphenous vein at hip and thigh level, unspecified leg, initial encounter | 1 |
| S75.221A | ICD10CM | Major laceration of greater saphenous vein at hip and thigh level, right leg, initial encounter | 1 |
| S75.222A | ICD10CM | Major laceration of greater saphenous vein at hip and thigh level, left leg, initial encounter | 1 |
| S75.229A | ICD10CM | Major laceration of greater saphenous vein at hip and thigh level, unspecified leg, initial encounter | 1 |
| S75.811A | ICD10CM | Laceration of other blood vessels at hip and thigh level, right leg, initial encounter | 1 |
| S75.812A | ICD10CM | Laceration of other blood vessels at hip and thigh level, left leg, initial encounter | 1 |
| S75.819A | ICD10CM | Laceration of other blood vessels at hip and thigh level, unspecified leg, initial encounter | 1 |
| S75.911A | ICD10CM | Laceration of unspecified blood vessel at hip and thigh level, right leg, initial encounter | 1 |
| S75.912A | ICD10CM | Laceration of unspecified blood vessel at hip and thigh level, left leg, initial encounter | 1 |
| S75.919A | ICD10CM | Laceration of unspecified blood vessel at hip and thigh level, unspecified leg, initial encounter | 1 |
| S80.00XA | ICD10CM | Contusion of unspecified knee, initial encounter | 1 |
| S80.01XA | ICD10CM | Contusion of right knee, initial encounter | 1 |
| S80.02XA | ICD10CM | Contusion of left knee, initial encounter | 1 |
| S80.10XA | ICD10CM | Contusion of unspecified lower leg, initial encounter | 1 |
| S80.11XA | ICD10CM | Contusion of right lower leg, initial encounter | 1 |
| S80.12XA | ICD10CM | Contusion of left lower leg, initial encounter | 1 |
| S85.011A | ICD10CM | Laceration of popliteal artery, right leg, initial encounter | 1 |
| S85.012A | ICD10CM | Laceration of popliteal artery, left leg, initial encounter | 1 |
| S85.019A | ICD10CM | Laceration of popliteal artery, unspecified leg, initial encounter | 1 |
| S85.111A | ICD10CM | Laceration of unspecified tibial artery, right leg, initial encounter | 1 |
| S85.112A | ICD10CM | Laceration of unspecified tibial artery, left leg, initial encounter | 1 |
| S85.119A | ICD10CM | Laceration of unspecified tibial artery, unspecified leg, initial encounter | 1 |
| S85.141A | ICD10CM | Laceration of anterior tibial artery, right leg, initial encounter |  |
| S85.142A | ICD10CM | Laceration of anterior tibial artery, left leg, initial encounter | 1 |
| S85.149A | ICD10CM | Laceration of anterior tibial artery, unspecified leg, initial encounter | 1 |
| S85.171A | ICD10CM | Laceration of posterior tibial artery, right leg, initial encounter | 1 |
| S85.172A | ICD10CM | Laceration of posterior tibial artery, left leg, initial encounter | 1 |
| S85.179A | ICD10CM | Laceration of posterior tibial artery, unspecified leg, initial encounter | 1 |
| S85.211A | ICD10CM | Laceration of peroneal artery, right leg, initial encounter | 1 |
| S85.212A | ICD10CM | Laceration of peroneal artery, left leg, initial encounter | 1 |
| S85.219A | ICD10CM | Laceration of peroneal artery, unspecified leg, initial encounter | 1 |
| S85.311A | ICD10CM | Laceration of greater saphenous vein at lower leg level, right leg, initial encounter | 1 |
| S85.312A | ICD10CM | Laceration of greater saphenous vein at lower leg level, left leg, initial encounter | 1 |
| S85.319A | ICD10CM | Laceration of greater saphenous vein at lower leg level, unspecified leg, initial encounter | 1 |
| S85.411A | ICD10CM | Laceration of lesser saphenous vein at lower leg level, right leg, initial encounter | 1 |
| S85.412A | ICD10CM | Laceration of lesser saphenous vein at lower leg level, left leg, initial encounter | 1 |
| S85.419A | ICD10CM | Laceration of lesser saphenous vein at lower leg level, unspecified leg, initial encounter | 1 |
| S85.511A | ICD10CM | Laceration of popliteal vein, right leg, initial encounter | 1 |
| S85.512A | ICD10CM | Laceration of popliteal vein, left leg, initial encounter | 1 |
| S85.519A | ICD10CM | Laceration of popliteal vein, unspecified leg, initial encounter | 1 |
| S85.811A | ICD10CM | Laceration of other blood vessels at lower leg level, right leg, initial encounter | 1 |
| S85.812A | ICD10CM | Laceration of other blood vessels at lower leg level, left leg, initial encounter | 1 |
| S85.819A | ICD10CM | Laceration of other blood vessels at lower leg level, unspecified leg, initial encounter | 1 |
| S85.911A | ICD10CM | Laceration of unspecified blood vessel at lower leg level, right leg, initial encounter | 1 |
| S85.912A | ICD10CM | Laceration of unspecified blood vessel at lower leg level, left leg, initial encounter | 1 |
| S85.919A | ICD10CM | Laceration of unspecified blood vessel at lower leg level, unspecified leg, initial encounter | 1 |
| S90.00XA | ICD10CM | Contusion of unspecified ankle, initial encounter | 1 |
| S90.01XA | ICD10CM | Contusion of right ankle, initial encounter | 1 |
| S90.02XA | ICD10CM | Contusion of left ankle, initial encounter | 1 |
| S90.111A | ICD10CM | Contusion of right great toe without damage to nail, initial encounter | 1 |
| S90.112A | ICD10CM | Contusion of left great toe without damage to nail, initial encounter | 1 |
| S90.119A | ICD10CM | Contusion of unspecified great toe without damage to nail, initial encounter | 1 |
| S90.121A | ICD10CM | Contusion of right lesser toe(s) without damage to nail, initial encounter | 1 |
| S90.122A | ICD10CM | Contusion of left lesser toe(s) without damage to nail, initial encounter | 1 |
| S90.129A | ICD10CM | Contusion of unspecified lesser toe(s) without damage to nail, initial encounter | 1 |
| S90.211A | ICD10CM | Contusion of right great toe with damage to nail, initial encounter | 1 |
| S90.212A | ICD10CM | Contusion of left great toe with damage to nail, initial encounter | 1 |
| S90.219A | ICD10CM | Contusion of unspecified great toe with damage to nail, initial encounter | 1 |
| S90.221A | ICD10CM | Contusion of right lesser toe(s) with damage to nail, initial encounter | 1 |
| S90.222A | ICD10CM | Contusion of left lesser toe(s) with damage to nail, initial encounter | 1 |
| S90.229A | ICD10CM | Contusion of unspecified lesser toe(s) with damage to nail, initial encounter | 1 |
| S90.30XA | ICD10CM | Contusion of unspecified foot, initial encounter | 1 |
| S90.31XA | ICD10CM | Contusion of right foot, initial encounter | 1 |
| S90.32XA | ICD10CM | Contusion of left foot, initial encounter | 1 |
| S95.011A | ICD10CM | Laceration of dorsal artery of right foot, initial encounter | 1 |
| S95.012A | ICD10CM | Laceration of dorsal artery of left foot, initial encounter | 1 |
| S95.019A | ICD10CM | Laceration of dorsal artery of unspecified foot, initial encounter | 1 |
| S95.111A | ICD10CM | Laceration of plantar artery of right foot, initial encounter | 1 |
| S95.112A | ICD10CM | Laceration of plantar artery of left foot, initial encounter | 1 |
| S95.119A | ICD10CM | Laceration of plantar artery of unspecified foot, initial encounter | 1 |
| S95.211A | ICD10CM | Laceration of dorsal vein of right foot, initial encounter | 1 |
| S95.212A | ICD10CM | Laceration of dorsal vein of left foot, initial encounter | 1 |
| S95.219A | ICD10CM | Laceration of dorsal vein of unspecified foot, initial encounter | 1 |
| S95.811A | ICD10CM | Laceration of other blood vessels at ankle and foot level, right leg, initial encounter | 1 |
| S95.812A | ICD10CM | Laceration of other blood vessels at ankle and foot level, left leg, initial encounter | 1 |
| S95.819A | ICD10CM | Laceration of other blood vessels at ankle and foot level, unspecified leg, initial encounter | 1 |
| S95.911A | ICD10CM | Laceration of unspecified blood vessel at ankle and foot level, right leg, initial encounter | 1 |
| S95.912A | ICD10CM | Laceration of unspecified blood vessel at ankle and foot level, left leg, initial encounter | 1 |
| S95.919A | ICD10CM | Laceration of unspecified blood vessel at ankle and foot level, unspecified leg, initial encounter | 1 |
| T79.2 | ICD10CM | Traumatic secondary and recurrent hemorrhage and seroma | 1 |
| T79.2XXA | ICD10CM | Traumatic secondary and recurrent hemorrhage and seroma, initial encounter | 1 |
| T82.83 | ICD10CM | Hemorrhage due to cardiac and vascular prosthetic devices, implants and grafts | 1 |
| T82.837 | ICD10CM | Hemorrhage due to cardiac prosthetic devices, implants and grafts | 1 |
| T82.837A | ICD10CM | Hemorrhage due to cardiac prosthetic devices, implants and grafts, initial encounter | 1 |
| T82.838 | ICD10CM | Hemorrhage due to vascular prosthetic devices, implants and grafts | 1 |
| T82.838A | ICD10CM | Hemorrhage due to vascular prosthetic devices, implants and grafts, initial encounter | 1 |
| T83.83 | ICD10CM | Hemorrhage due to genitourinary prosthetic devices, implants and grafts | 1 |
| T83.83XA | ICD10CM | Hemorrhage due to genitourinary prosthetic devices, implants and grafts, initial encounter | 1 |
| T84.83 | ICD10CM | Hemorrhage due to internal orthopedic prosthetic devices, implants and grafts | 1 |
| T84.83XA | ICD10CM | Hemorrhage due to internal orthopedic prosthetic devices, implants and grafts, initial encounter | 1 |
| T85.830 | ICD10CM | Hemorrhage due to nervous system prosthetic devices, implants and grafts | 1 |
| T85.830A | ICD10CM | Hemorrhage due to nervous system prosthetic devices, implants and grafts, initial encounter | 1 |
| T85.838 | ICD10CM | Hemorrhage due to other internal prosthetic devices, implants and grafts | 1 |
| T85.838A | ICD10CM | Hemorrhage due to other internal prosthetic devices, implants and grafts, initial encounter | 1 |
| 246.3 | ICD9CM | Hemorrhage and infarction of thyroid |  |
| 287.8 | ICD9CM | Other specified hemorrhagic conditions |  |
| 287.9 | ICD9CM | Unspecified hemorrhagic conditions |  |
| 372.72 | ICD9CM | Conjunctival hemorrhage |  |
| 374.81 | ICD9CM | Hemorrhage of eyelid |  |
| 377.42 | ICD9CM | Hemorrhage in optic nerve sheaths |  |
| 456.0 | ICD9CM | Esophageal varices with bleeding |  |
| 456.20 | ICD9CM | Esophageal varices in diseases classified elsewhere, with bleeding |  |
| 459.0 | ICD9CM | Hemorrhage, unspecified |  |
| 530.21 | ICD9CM | Ulcer of esophagus with bleeding |  |
| 530.7 | ICD9CM | Gastroesophageal laceration-hemorrhage syndrome |  |
| 530.82 | ICD9CM | Esophageal hemorrhage |  |
| 531.0 | ICD9CM | Acute gastric ulcer with hemorrhage |  |
| 531.00 | ICD9CM | Acute gastric ulcer with hemorrhage, without mention of obstruction |  |
| 531.01 | ICD9CM | Acute gastric ulcer with hemorrhage, with obstruction |  |
| 531.2 | ICD9CM | Acute gastric ulcer with hemorrhage and perforation |  |
| 531.20 | ICD9CM | Acute gastric ulcer with hemorrhage and perforation, without mention of obstruction |  |
| 531.21 | ICD9CM | Acute gastric ulcer with hemorrhage and perforation, with obstruction |  |
| 531.4 | ICD9CM | Chronic or unspecified gastric ulcer with hemorrhage |  |
| 531.40 | ICD9CM | Chronic or unspecified gastric ulcer with hemorrhage, without mention of obstruction |  |
| 531.41 | ICD9CM | Chronic or unspecified gastric ulcer with hemorrhage, with obstruction |  |
| 531.6 | ICD9CM | Chronic or unspecified gastric ulcer with hemorrhage and perforation |  |
| 531.60 | ICD9CM | Chronic or unspecified gastric ulcer with hemorrhage and perforation, without mention of obstruction |  |
| 531.61 | ICD9CM | Chronic or unspecified gastric ulcer with hemorrhage and perforation, with obstruction |  |
| 532.0 | ICD9CM | Acute duodenal ulcer with hemorrhage |  |
| 532.00 | ICD9CM | Acute duodenal ulcer with hemorrhage, without mention of obstruction |  |
| 532.01 | ICD9CM | Acute duodenal ulcer with hemorrhage, with obstruction |  |
| 532.2 | ICD9CM | Acute duodenal ulcer with hemorrhage and perforation |  |
| 532.20 | ICD9CM | Acute duodenal ulcer with hemorrhage and perforation, without mention of obstruction |  |
| 532.21 | ICD9CM | Acute duodenal ulcer with hemorrhage and perforation, with obstruction |  |
| 532.4 | ICD9CM | Chronic or unspecified duodenal ulcer with hemorrhage |  |
| 532.40 | ICD9CM | Chronic or unspecified duodenal ulcer with hemorrhage, without mention of obstruction |  |
| 532.41 | ICD9CM | Chronic or unspecified duodenal ulcer with hemorrhage, with obstruction |  |
| 532.6 | ICD9CM | Chronic or unspecified duodenal ulcer with hemorrhage and perforation |  |
| 532.60 | ICD9CM | Chronic or unspecified duodenal ulcer with hemorrhage and perforation, without mention of obstruction |  |
| 532.61 | ICD9CM | Chronic or unspecified duodenal ulcer with hemorrhage and perforation, with obstruction |  |
| 533.0 | ICD9CM | Acute peptic ulcer of unspecified site with hemorrhage |  |
| 533.00 | ICD9CM | Acute peptic ulcer of unspecified site with hemorrhage, without mention of obstruction |  |
| 533.01 | ICD9CM | Acute peptic ulcer of unspecified site with hemorrhage, with obstruction |  |
| 533.2 | ICD9CM | Acute peptic ulcer of unspecified site with hemorrhage and perforation |  |
| 533.20 | ICD9CM | Acute peptic ulcer of unspecified site with hemorrhage and perforation, without mention of obstruction |  |
| 533.21 | ICD9CM | Acute peptic ulcer of unspecified site with hemorrhage and perforation, with obstruction |  |
| 533.4 | ICD9CM | Chronic or unspecified peptic ulcer of unspecified site with hemorrhage |  |
| 533.40 | ICD9CM | Chronic or unspecified peptic ulcer of unspecified site with hemorrhage, without mention of obstruction |  |
| 533.41 | ICD9CM | Chronic or unspecified peptic ulcer of unspecified site with hemorrhage, with obstruction |  |
| 533.6 | ICD9CM | Chronic or unspecified peptic ulcer of unspecified site with hemorrhage and perforation |  |
| 533.60 | ICD9CM | Chronic or unspecified peptic ulcer of unspecified site with hemorrhage and perforation, without mention of obstruction |  |
| 533.61 | ICD9CM | Chronic or unspecified peptic ulcer of unspecified site with hemorrhage and perforation, with obstruction |  |
| 534.0 | ICD9CM | Acute gastrojejunal ulcer with hemorrhage |  |
| 534.00 | ICD9CM | Acute gastrojejunal ulcer with hemorrhage, without mention of obstruction |  |
| 534.01 | ICD9CM | Acute gastrojejunal ulcer, with hemorrhage, with obstruction |  |
| 534.2 | ICD9CM | Acute gastrojejunal ulcer with hemorrhage and perforation |  |
| 534.20 | ICD9CM | Acute gastrojejunal ulcer with hemorrhage and perforation, without mention of obstruction |  |
| 534.21 | ICD9CM | Acute gastrojejunal ulcer with hemorrhage and perforation, with obstruction |  |
| 534.4 | ICD9CM | Chronic or unspecified gastrojejunal ulcer with hemorrhage |  |
| 534.40 | ICD9CM | Chronic or unspecified gastrojejunal ulcer with hemorrhage, without mention of obstruction |  |
| 534.41 | ICD9CM | Chronic or unspecified gastrojejunal ulcer, with hemorrhage, with obstruction |  |
| 534.6 | ICD9CM | Chronic or unspecified gastrojejunal ulcer with hemorrhage and perforation |  |
| 534.60 | ICD9CM | Chronic or unspecified gastrojejunal ulcer with hemorrhage and perforation, without mention of obstruction |  |
| 534.61 | ICD9CM | Chronic or unspecified gastrojejunal ulcer with hemorrhage and perforation, with obstruction |  |
| 535.01 | ICD9CM | Acute gastritis, with hemorrhage |  |
| 535.11 | ICD9CM | Atrophic gastritis, with hemorrhage |  |
| 535.21 | ICD9CM | Gastric mucosal hypertrophy, with hemorrhage |  |
| 535.31 | ICD9CM | Alcoholic gastritis, with hemorrhage |  |
| 535.41 | ICD9CM | Other specified gastritis, with hemorrhage |  |
| 535.51 | ICD9CM | Unspecified gastritis and gastroduodenitis, with hemorrhage |  |
| 535.61 | ICD9CM | Duodenitis, with hemorrhage |  |
| 535.71 | ICD9CM | Eosinophilic gastritis, with hemorrhage |  |
| 537.83 | ICD9CM | Angiodysplasia of stomach and duodenum with hemorrhage |  |
| 537.84 | ICD9CM | Dieulafoy lesion (hemorrhagic) of stomach and duodenum |  |
| 562.02 | ICD9CM | Diverticulosis of small intestine with hemorrhage |  |
| 562.03 | ICD9CM | Diverticulitis of small intestine with hemorrhage |  |
| 562.12 | ICD9CM | Diverticulosis of colon with hemorrhage |  |
| 562.13 | ICD9CM | Diverticulitis of colon with hemorrhage |  |
| 568.81 | ICD9CM | Hemoperitoneum (nontraumatic) |  |
| 569.3 | ICD9CM | Hemorrhage of rectum and anus |  |
| 569.85 | ICD9CM | Angiodysplasia of intestine with hemorrhage |  |
| 569.86 | ICD9CM | Dieulafoy lesion (hemorrhagic) of intestine |  |
| 578 | ICD9CM | Gastrointestinal hemorrhage |  |
| 578.0 | ICD9CM | Hematemesis |  |
| 578.1 | ICD9CM | Blood in stool |  |
| 578.9 | ICD9CM | Hemorrhage of gastrointestinal tract, unspecified |  |
| 593.81 | ICD9CM | Vascular disorders of kidney |  |
| 596.7 | ICD9CM | Hemorrhage into bladder wall |  |
| 599.71 | ICD9CM | Gross hematuria |  |
| 602.1 | ICD9CM | Congestion or hemorrhage of prostate |  |
| 620.7 | ICD9CM | Hematoma of broad ligament |  |
| 621.4 | ICD9CM | Hematometra |  |
| 623.6 | ICD9CM | Vaginal hematoma |  |
| 624.5 | ICD9CM | Hematoma of vulva |  |
| 626.2 | ICD9CM | Excessive or frequent menstruation |  |
| 626.5 | ICD9CM | Ovulation bleeding |  |
| 626.6 | ICD9CM | Metrorrhagia |  |
| 626.7 | ICD9CM | Postcoital bleeding |  |
| 626.8 | ICD9CM | Other disorders of menstruation and other abnormal bleeding from female genital tract |  |
| 626.9 | ICD9CM | Unspecified disorders of menstruation and other abnormal bleeding from female genital tract |  |
| 627.1 | ICD9CM | Postmenopausal bleeding |  |
| 629.0 | ICD9CM | Hematocele, female, not elsewhere classified |  |
| 729.92 | ICD9CM | Nontraumatic hematoma of soft tissue |  |
| 782.7 | ICD9CM | Spontaneous ecchymoses |  |
| 784.7 | ICD9CM | Epistaxis |  |
| 784.8 | ICD9CM | Hemorrhage from throat |  |
| 786.3 | ICD9CM | Hemoptysis |  |
| 786.30 | ICD9CM | Hemoptysis, unspecified |  |
| 786.31 | ICD9CM | Acute idiopathic pulmonary hemorrhage in infants [aiphi] |  |
| 786.39 | ICD9CM | Other hemoptysis |  |
| 790.01 | ICD9CM | Precipitous drop in hematocrit |  |
| D68.32 | ICD10CM | Hemorrhagic disorder due to extrinsic circulating anticoagulants |  |
| H11.3 | ICD10CM | Conjunctival hemorrhage |  |
| H11.30 | ICD10CM | Conjunctival hemorrhage, unspecified eye |  |
| H11.31 | ICD10CM | Conjunctival hemorrhage, right eye |  |
| H11.32 | ICD10CM | Conjunctival hemorrhage, left eye |  |
| H11.33 | ICD10CM | Conjunctival hemorrhage, bilateral |  |
| H47.02 | ICD10CM | Hemorrhage in optic nerve sheath |  |
| H47.021 | ICD10CM | Hemorrhage in optic nerve sheath, right eye |  |
| H47.022 | ICD10CM | Hemorrhage in optic nerve sheath, left eye |  |
| H47.023 | ICD10CM | Hemorrhage in optic nerve sheath, bilateral |  |
| H47.029 | ICD10CM | Hemorrhage in optic nerve sheath, unspecified eye |  |
| H60.32 | ICD10CM | Hemorrhagic otitis externa |  |
| H60.321 | ICD10CM | Hemorrhagic otitis externa, right ear |  |
| H60.322 | ICD10CM | Hemorrhagic otitis externa, left ear |  |
| H60.323 | ICD10CM | Hemorrhagic otitis externa, bilateral |  |
| H60.329 | ICD10CM | Hemorrhagic otitis externa, unspecified ear |  |
| H92.2 | ICD10CM | Otorrhagia |  |
| H92.20 | ICD10CM | Otorrhagia, unspecified ear |  |
| H92.21 | ICD10CM | Otorrhagia, right ear |  |
| H92.22 | ICD10CM | Otorrhagia, left ear |  |
| H92.23 | ICD10CM | Otorrhagia, bilateral |  |
| I85.01 | ICD10CM | Esophageal varices with bleeding |  |
| I85.11 | ICD10CM | Secondary esophageal varices with bleeding |  |
| J94.2 | ICD10CM | Hemothorax |  |
| K20.81 | ICD10CM | Other esophagitis with bleeding |  |
| K20.91 | ICD10CM | Esophagitis, unspecified with bleeding |  |
| K22.11 | ICD10CM | Ulcer of esophagus with bleeding |  |
| K22.6 | ICD10CM | Gastro-esophageal laceration-hemorrhage syndrome |  |
| K25.0 | ICD10CM | Acute gastric ulcer with hemorrhage |  |
| K25.2 | ICD10CM | Acute gastric ulcer with both hemorrhage and perforation |  |
| K25.4 | ICD10CM | Chronic or unspecified gastric ulcer with hemorrhage |  |
| K25.6 | ICD10CM | Chronic or unspecified gastric ulcer with both hemorrhage and perforation |  |
| K26.0 | ICD10CM | Acute duodenal ulcer with hemorrhage |  |
| K26.2 | ICD10CM | Acute duodenal ulcer with both hemorrhage and perforation |  |
| K26.4 | ICD10CM | Chronic or unspecified duodenal ulcer with hemorrhage |  |
| K26.6 | ICD10CM | Chronic or unspecified duodenal ulcer with both hemorrhage and perforation |  |
| K27.0 | ICD10CM | Acute peptic ulcer, site unspecified, with hemorrhage |  |
| K27.2 | ICD10CM | Acute peptic ulcer, site unspecified, with both hemorrhage and perforation |  |
| K27.4 | ICD10CM | Chronic or unspecified peptic ulcer, site unspecified, with hemorrhage |  |
| K27.6 | ICD10CM | Chronic or unspecified peptic ulcer, site unspecified, with both hemorrhage and perforation |  |
| K28.0 | ICD10CM | Acute gastrojejunal ulcer with hemorrhage |  |
| K28.2 | ICD10CM | Acute gastrojejunal ulcer with both hemorrhage and perforation |  |
| K28.4 | ICD10CM | Chronic or unspecified gastrojejunal ulcer with hemorrhage |  |
| K28.6 | ICD10CM | Chronic or unspecified gastrojejunal ulcer with both hemorrhage and perforation |  |
| K29.01 | ICD10CM | Acute gastritis with bleeding |  |
| K29.21 | ICD10CM | Alcoholic gastritis with bleeding |  |
| K29.31 | ICD10CM | Chronic superficial gastritis with bleeding |  |
| K29.41 | ICD10CM | Chronic atrophic gastritis with bleeding |  |
| K29.51 | ICD10CM | Unspecified chronic gastritis with bleeding |  |
| K29.61 | ICD10CM | Other gastritis with bleeding |  |
| K29.71 | ICD10CM | Gastritis, unspecified, with bleeding |  |
| K29.81 | ICD10CM | Duodenitis with bleeding |  |
| K29.91 | ICD10CM | Gastroduodenitis, unspecified, with bleeding |  |
| K31.811 | ICD10CM | Angiodysplasia of stomach and duodenum with bleeding |  |
| K50.011 | ICD10CM | Crohn's disease of small intestine with rectal bleeding |  |
| K50.111 | ICD10CM | Crohn's disease of large intestine with rectal bleeding |  |
| K50.811 | ICD10CM | Crohn's disease of both small and large intestine with rectal bleeding |  |
| K50.911 | ICD10CM | Crohn's disease, unspecified, with rectal bleeding |  |
| K51.011 | ICD10CM | Ulcerative (chronic) pancolitis with rectal bleeding |  |
| K51.211 | ICD10CM | Ulcerative (chronic) proctitis with rectal bleeding |  |
| K51.311 | ICD10CM | Ulcerative (chronic) rectosigmoiditis with rectal bleeding |  |
| K51.411 | ICD10CM | Inflammatory polyps of colon with rectal bleeding |  |
| K51.511 | ICD10CM | Left sided colitis with rectal bleeding |  |
| K51.811 | ICD10CM | Other ulcerative colitis with rectal bleeding |  |
| K51.911 | ICD10CM | Ulcerative colitis, unspecified with rectal bleeding |  |
| K55.21 | ICD10CM | Angiodysplasia of colon with hemorrhage |  |
| K57.01 | ICD10CM | Diverticulitis of small intestine with perforation and abscess with bleeding |  |
| K57.11 | ICD10CM | Diverticulosis of small intestine without perforation or abscess with bleeding |  |
| K57.13 | ICD10CM | Diverticulitis of small intestine without perforation or abscess with bleeding |  |
| K57.21 | ICD10CM | Diverticulitis of large intestine with perforation and abscess with bleeding |  |
| K57.31 | ICD10CM | Diverticulosis of large intestine without perforation or abscess with bleeding |  |
| K57.33 | ICD10CM | Diverticulitis of large intestine without perforation or abscess with bleeding |  |
| K57.41 | ICD10CM | Diverticulitis of both small and large intestine with perforation and abscess with bleeding |  |
| K57.51 | ICD10CM | Diverticulosis of both small and large intestine without perforation or abscess with bleeding |  |
| K57.53 | ICD10CM | Diverticulitis of both small and large intestine without perforation or abscess with bleeding |  |
| K57.81 | ICD10CM | Diverticulitis of intestine, part unspecified, with perforation and abscess with bleeding |  |
| K57.91 | ICD10CM | Diverticulosis of intestine, part unspecified, without perforation or abscess with bleeding |  |
| K57.93 | ICD10CM | Diverticulitis of intestine, part unspecified, without perforation or abscess with bleeding |  |
| K62.5 | ICD10CM | Hemorrhage of anus and rectum |  |
| K63.81 | ICD10CM | Dieulafoy lesion of intestine |  |
| K66.1 | ICD10CM | Hemoperitoneum |  |
| K92.0 | ICD10CM | Hematemesis |  |
| K92.1 | ICD10CM | Melena |  |
| K92.2 | ICD10CM | Gastrointestinal hemorrhage, unspecified |  |
| K94.01 | ICD10CM | Colostomy hemorrhage |  |
| K94.11 | ICD10CM | Enterostomy hemorrhage |  |
| K94.21 | ICD10CM | Gastrostomy hemorrhage |  |
| K94.31 | ICD10CM | Esophagostomy hemorrhage |  |
| M79.81 | ICD10CM | Nontraumatic hematoma of soft tissue |  |
| N30.01 | ICD10CM | Acute cystitis with hematuria |  |
| N30.11 | ICD10CM | Interstitial cystitis (chronic) with hematuria |  |
| N30.21 | ICD10CM | Other chronic cystitis with hematuria |  |
| N30.31 | ICD10CM | Trigonitis with hematuria |  |
| N30.41 | ICD10CM | Irradiation cystitis with hematuria |  |
| N30.81 | ICD10CM | Other cystitis with hematuria |  |
| N30.91 | ICD10CM | Cystitis, unspecified with hematuria |  |
| N42.1 | ICD10CM | Congestion and hemorrhage of prostate |  |
| N83.7 | ICD10CM | Hematoma of broad ligament |  |
| N85.7 | ICD10CM | Hematometra |  |
| N92.0 | ICD10CM | Excessive and frequent menstruation with regular cycle |  |
| N92.1 | ICD10CM | Excessive and frequent menstruation with irregular cycle |  |
| N92.3 | ICD10CM | Ovulation bleeding |  |
| N92.4 | ICD10CM | Excessive bleeding in the premenopausal period |  |
| N93 | ICD10CM | Other abnormal uterine and vaginal bleeding |  |
| N93.0 | ICD10CM | Postcoital and contact bleeding |  |
| N93.1 | ICD10CM | Pre-pubertal vaginal bleeding |  |
| N93.8 | ICD10CM | Other specified abnormal uterine and vaginal bleeding |  |
| N93.9 | ICD10CM | Abnormal uterine and vaginal bleeding, unspecified |  |
| N95.0 | ICD10CM | Postmenopausal bleeding |  |
| R04.0 | ICD10CM | Epistaxis |  |
| R04.1 | ICD10CM | Hemorrhage from throat |  |
| R04.2 | ICD10CM | Hemoptysis |  |
| R04.81 | ICD10CM | Acute idiopathic pulmonary hemorrhage in infants |  |
| R04.89 | ICD10CM | Hemorrhage from other sites in respiratory passages |  |
| R04.9 | ICD10CM | Hemorrhage from respiratory passages, unspecified |  |
| R23.3 | ICD10CM | Spontaneous ecchymoses |  |
| R31.0 | ICD10CM | Gross hematuria |  |
| R58 | ICD10CM | Hemorrhage, not elsewhere classified |  |
| R71.0 | ICD10CM | Precipitous drop in hematocrit |  |

# **Supplementary Table S1c: Possible bleed codes, ICD-9-CM and ICD-10-CM**

| **Code** | **Code Type** | **Description** | **Trauma Flag Code** |
| --- | --- | --- | --- |
| 280.0 | ICD9CM | Iron deficiency anemia secondary to blood loss (chronic) |  |
| 285.1 | ICD9CM | Acute posthemorrhagic anemia |  |
| 285.9 | ICD9CM | Anemia, unspecified |  |
| 455.0 | ICD9CM | Internal hemorrhoids without mention of complication |  |
| 455.1 | ICD9CM | Internal thrombosed hemorrhoids |  |
| 455.2 | ICD9CM | Internal hemorrhoids with other complication |  |
| 455.3 | ICD9CM | External hemorrhoids without mention of complication |  |
| 455.4 | ICD9CM | External thrombosed hemorrhoids |  |
| 455.5 | ICD9CM | External hemorrhoids with other complication |  |
| 455.6 | ICD9CM | Unspecified hemorrhoids without mention of complication |  |
| 455.7 | ICD9CM | Unspecified thrombosed hemorrhoids |  |
| 455.8 | ICD9CM | Unspecified hemorrhoids with other complication |  |
| 455.9 | ICD9CM | Residual hemorrhoidal skin tags |  |
| 530.10 | ICD9CM | Esophagitis, unspecified |  |
| 530.11 | ICD9CM | Reflux esophagitis |  |
| 530.12 | ICD9CM | Acute esophagitis |  |
| 530.13 | ICD9CM | Eosinophilic esophagitis |  |
| 530.19 | ICD9CM | Other esophagitis |  |
| 531.10 | ICD9CM | Acute gastric ulcer with perforation, without mention of obstruction |  |
| 531.11 | ICD9CM | Acute gastric ulcer with perforation, with obstruction |  |
| 531.30 | ICD9CM | Acute gastric ulcer without mention of hemorrhage or perforation, without mention of obstruction |  |
| 531.31 | ICD9CM | Acute gastric ulcer without mention of hemorrhage or perforation, with obstruction |  |
| 531.50 | ICD9CM | Chronic or unspecified gastric ulcer with perforation, without mention of obstruction |  |
| 531.51 | ICD9CM | Chronic or unspecified gastric ulcer with perforation, with obstruction |  |
| 531.70 | ICD9CM | Chronic gastric ulcer without mention of hemorrhage or perforation, without mention of obstruction |  |
| 531.71 | ICD9CM | Chronic gastric ulcer without mention of hemorrhage or perforation, with obstruction |  |
| 531.90 | ICD9CM | Gastric ulcer, unspecified as acute or chronic, without mention of hemorrhage or perforation, without mention of obstruction |  |
| 531.91 | ICD9CM | Gastric ulcer, unspecified as acute or chronic, without mention of hemorrhage or perforation, with obstruction |  |
| 532.30 | ICD9CM | Acute duodenal ulcer without mention of hemorrhage or perforation, without mention of obstruction |  |
| 532.31 | ICD9CM | Acute duodenal ulcer without mention of hemorrhage or perforation, with obstruction |  |
| 532.50 | ICD9CM | Chronic or unspecified duodenal ulcer with perforation, without mention of obstruction |  |
| 532.51 | ICD9CM | Chronic or unspecified duodenal ulcer with perforation, with obstruction |  |
| 532.70 | ICD9CM | Chronic duodenal ulcer without mention of hemorrhage or perforation, without mention of obstruction |  |
| 532.71 | ICD9CM | Chronic duodenal ulcer without mention of hemorrhage or perforation, with obstruction |  |
| 532.90 | ICD9CM | Duodenal ulcer, unspecified as acute or chronic, without hemorrhage or perforation, without mention of obstruction |  |
| 532.91 | ICD9CM | Duodenal ulcer, unspecified as acute or chronic, without mention of hemorrhage or perforation, with obstruction |  |
| 533.10 | ICD9CM | Acute peptic ulcer of unspecified site with perforation, without mention of obstruction |  |
| 533.11 | ICD9CM | Acute peptic ulcer of unspecified site with perforation, with obstruction |  |
| 533.30 | ICD9CM | Acute peptic ulcer of unspecified site without mention of hemorrhage and perforation, without mention of obstruction |  |
| 533.31 | ICD9CM | Acute peptic ulcer of unspecified site without mention of hemorrhage and perforation, with obstruction |  |
| 533.50 | ICD9CM | Chronic or unspecified peptic ulcer of unspecified site with perforation, without mention of obstruction |  |
| 533.51 | ICD9CM | Chronic or unspecified peptic ulcer of unspecified site with perforation, with obstruction |  |
| 533.70 | ICD9CM | Chronic peptic ulcer of unspecified site without mention of hemorrhage or perforation, without mention of obstruction |  |
| 533.71 | ICD9CM | Chronic peptic ulcer of unspecified site without mention of hemorrhage or perforation, with obstruction |  |
| 533.90 | ICD9CM | Peptic ulcer of unspecified site, unspecified as acute or chronic, without mention of hemorrhage or perforation, without mention of obstruction |  |
| 533.91 | ICD9CM | Peptic ulcer of unspecified site, unspecified as acute or chronic, without mention of hemorrhage or perforation, with obstruction |  |
| 534.10 | ICD9CM | Acute gastrojejunal ulcer with perforation, without mention of obstruction |  |
| 534.11 | ICD9CM | Acute gastrojejunal ulcer with perforation, with obstruction |  |
| 534.30 | ICD9CM | Acute gastrojejunal ulcer without mention of hemorrhage or perforation, without mention of obstruction |  |
| 534.31 | ICD9CM | Acute gastrojejunal ulcer without mention of hemorrhage or perforation, with obstruction |  |
| 534.50 | ICD9CM | Chronic or unspecified gastrojejunal ulcer with perforation, without mention of obstruction |  |
| 534.51 | ICD9CM | Chronic or unspecified gastrojejunal ulcer with perforation, with obstruction |  |
| 534.70 | ICD9CM | Chronic gastrojejunal ulcer without mention of hemorrhage or perforation, without mention of obstruction |  |
| 534.71 | ICD9CM | Chronic gastrojejunal ulcer without mention of hemorrhage or perforation, with obstruction |  |
| 534.90 | ICD9CM | Gastrojejunal ulcer, unspecified as acute or chronic, without mention of hemorrhage or perforation, without mention of obstruction |  |
| 534.91 | ICD9CM | Gastrojejunal ulcer, unspecified as acute or chronic, without mention of hemorrhage or perforation, with obstruction |  |
| 535.00 | ICD9CM | Acute gastritis, without mention of hemorrhage |  |
| 535.10 | ICD9CM | Atrophic gastritis, without mention of hemorrhage |  |
| 535.20 | ICD9CM | Gastric mucosal hypertrophy, without mention of hemorrhage |  |
| 535.30 | ICD9CM | Alcoholic gastritis, without mention of hemorrhage |  |
| 535.40 | ICD9CM | Other specified gastritis, without mention of hemorrhage |  |
| 535.50 | ICD9CM | Unspecified gastritis and gastroduodenitis, without mention of hemorrhage |  |
| 535.60 | ICD9CM | Duodenitis, without mention of hemorrhage |  |
| 562.00 | ICD9CM | Diverticulosis of small intestine (without mention of hemorrhage) |  |
| 562.01 | ICD9CM | Diverticulitis of small intestine (without mention of hemorrhage) |  |
| 562.10 | ICD9CM | Diverticulosis of colon (without mention of hemorrhage) |  |
| 562.11 | ICD9CM | Diverticulitis of colon (without mention of hemorrhage) |  |
| 599.70 | ICD9CM | Hematuria, unspecified |  |
| 599.72 | ICD9CM | Microscopic hematuria |  |
| 790.92 | ICD9CM | Abnormal coagulation profile |  |
| D50.0 | ICD10CM | Iron deficiency anemia secondary to blood loss (chronic) |  |
| D62 | ICD10CM | Acute posthemorrhagic anemia |  |
| D64.9 | ICD10CM | Anemia, unspecified |  |
| K20.0 | ICD10CM | Eosinophilic esophagitis |  |
| K20.8 | ICD10CM | Other esophagitis |  |
| K20.9 | ICD10CM | Esophagitis, unspecified |  |
| K21.0 | ICD10CM | Gastro-esophageal reflux disease with esophagitis |  |
| K25.1 | ICD10CM | Acute gastric ulcer with perforation |  |
| K25.3 | ICD10CM | Acute gastric ulcer without hemorrhage or perforation |  |
| K25.5 | ICD10CM | Chronic or unspecified gastric ulcer with perforation |  |
| K25.7 | ICD10CM | Chronic gastric ulcer without hemorrhage or perforation |  |
| K25.9 | ICD10CM | Gastric ulcer, unspecified as acute or chronic, without hemorrhage or perforation |  |
| K26.3 | ICD10CM | Acute duodenal ulcer without hemorrhage or perforation |  |
| K26.5 | ICD10CM | Chronic or unspecified duodenal ulcer with perforation |  |
| K26.7 | ICD10CM | Chronic duodenal ulcer without hemorrhage or perforation |  |
| K26.9 | ICD10CM | Duodenal ulcer, unspecified as acute or chronic, without hemorrhage or perforation |  |
| K27.1 | ICD10CM | Acute peptic ulcer, site unspecified, with perforation |  |
| K27.3 | ICD10CM | Acute peptic ulcer, site unspecified, without hemorrhage or perforation |  |
| K27.5 | ICD10CM | Chronic or unspecified peptic ulcer, site unspecified, with perforation |  |
| K27.7 | ICD10CM | Chronic peptic ulcer, site unspecified, without hemorrhage or perforation |  |
| K27.9 | ICD10CM | Peptic ulcer, site unspecified, unspecified as acute or chronic, without hemorrhage or perforation |  |
| K28.1 | ICD10CM | Acute gastrojejunal ulcer with perforation |  |
| K28.3 | ICD10CM | Acute gastrojejunal ulcer without hemorrhage or perforation |  |
| K28.5 | ICD10CM | Chronic or unspecified gastrojejunal ulcer with perforation |  |
| K28.7 | ICD10CM | Chronic gastrojejunal ulcer without hemorrhage or perforation |  |
| K28.9 | ICD10CM | Gastrojejunal ulcer, unspecified as acute or chronic, without hemorrhage or perforation |  |
| K29.00 | ICD10CM | Acute gastritis without bleeding |  |
| K29.20 | ICD10CM | Alcoholic gastritis without bleeding |  |
| K29.30 | ICD10CM | Chronic superficial gastritis without bleeding |  |
| K29.40 | ICD10CM | Chronic atrophic gastritis without bleeding |  |
| K29.50 | ICD10CM | Unspecified chronic gastritis without bleeding |  |
| K29.60 | ICD10CM | Other gastritis without bleeding |  |
| K29.70 | ICD10CM | Gastritis, unspecified, without bleeding |  |
| K29.80 | ICD10CM | Duodenitis without bleeding |  |
| K29.90 | ICD10CM | Gastroduodenitis, unspecified, without bleeding |  |
| K56.699 | ICD10CM | Other intestinal obstruction unspecified as to partial versus complete obstruction |  |
| K57.00 | ICD10CM | Diverticulitis of small intestine with perforation and abscess without bleeding |  |
| K57.10 | ICD10CM | Diverticulosis of small intestine without perforation or abscess without bleeding |  |
| K57.12 | ICD10CM | Diverticulitis of small intestine without perforation or abscess without bleeding |  |
| K57.20 | ICD10CM | Diverticulitis of large intestine with perforation and abscess without bleeding |  |
| K57.30 | ICD10CM | Diverticulosis of large intestine without perforation or abscess without bleeding |  |
| K57.32 | ICD10CM | Diverticulitis of large intestine without perforation or abscess without bleeding |  |
| K57.40 | ICD10CM | Diverticulitis of both small and large intestine with perforation and abscess without bleeding |  |
| K57.50 | ICD10CM | Diverticulosis of both small and large intestine without perforation or abscess without bleeding |  |
| K57.52 | ICD10CM | Diverticulitis of both small and large intestine without perforation or abscess without bleeding |  |
| K57.80 | ICD10CM | Diverticulitis of intestine, part unspecified, with perforation and abscess without bleeding |  |
| K57.90 | ICD10CM | Diverticulosis of intestine, part unspecified, without perforation or abscess without bleeding |  |
| K57.92 | ICD10CM | Diverticulitis of intestine, part unspecified, without perforation or abscess without bleeding |  |
| K64.0 | ICD10CM | First degree hemorrhoids |  |
| K64.1 | ICD10CM | Second degree hemorrhoids |  |
| K64.2 | ICD10CM | Third degree hemorrhoids |  |
| K64.3 | ICD10CM | Fourth degree hemorrhoids |  |
| K64.4 | ICD10CM | Residual hemorrhoidal skin tags |  |
| K64.5 | ICD10CM | Perianal venous thrombosis |  |
| K64.8 | ICD10CM | Other hemorrhoids |  |
| K64.9 | ICD10CM | Unspecified hemorrhoids |  |
| R31.1 | ICD10CM | Benign essential microscopic hematuria |  |
| R31.2 | ICD10CM | Other microscopic hematuria |  |
| R31.21 | ICD10CM | Asymptomatic microscopic hematuria |  |
| R31.29 | ICD10CM | Other microscopic hematuria |  |
| R31.9 | ICD10CM | Hematuria, unspecified |  |
| R79.1 | ICD10CM | Abnormal coagulation profile |  |

# **Supplementary Table S1d. Red blood cell/whole blood transfusion procedural codes**

| **Code** | **Code Type** | **Description** |
| --- | --- | --- |
|  |  |  |
| 36430 | CPT | Transfusion, blood or blood components |
| P9010 | HCPCS | Blood (whole), for transfusion, per unit |
| P9011 | HCPCS | Blood, split unit |
| P9012 | HCPCS | Cryoprecipitate, each unit |
| P9016 | HCPCS | Red blood cells, leukocytes reduced, each unit |
| P9021 | HCPCS | Red blood cells, each unit |
| P9022 | HCPCS | Red blood cells, washed, each unit |
| 30273H1 | ICD-10-PCS | Transfusion of Nonautologous Whole Blood into Products of Conception, Circulatory, Percutaneous Approach |
| 30233H0 | ICD-10-PCS | Transfusion of Autologous Whole Blood into Peripheral Vein, Percutaneous Approach |
| 30233H1 | ICD-10-PCS | Transfusion of Nonautologous Whole Blood into Peripheral Vein, Percutaneous Approach |
| 30243H0 | ICD-10-PCS | Transfusion of Autologous Whole Blood into Central Vein, Percutaneous Approach |
| 30243H1 | ICD-10-PCS | Transfusion of Nonautologous Whole Blood into Central Vein, Percutaneous Approach |
| 30273N1 | ICD-10-PCS | Transfusion of Nonautologous Red Blood Cells into Products of Conception, Circulatory, Percutaneous Approach |
| 30273P1 | ICD-10-PCS | Transfusion of Nonautologous Frozen Red Cells into Products of Conception, Circulatory, Percutaneous Approach |
| 30233N0 | ICD-10-PCS | Transfusion of Autologous Red Blood Cells into Peripheral Vein, Percutaneous Approach |
| 30233N1 | ICD-10-PCS | Transfusion of Nonautologous Red Blood Cells into Peripheral Vein, Percutaneous Approach |
| 30243N0 | ICD-10-PCS | Transfusion of Autologous Red Blood Cells into Central Vein, Percutaneous Approach |
| 30233P0 | ICD-10-PCS | Transfusion of Autologous Frozen Red Cells into Peripheral Vein, Percutaneous Approach |
| 30243N1 | ICD-10-PCS | Transfusion of Nonautologous Red Blood Cells into Central Vein, Percutaneous Approach |
| 30243P0 | ICD-10-PCS | Transfusion of Autologous Frozen Red Cells into Central Vein, Percutaneous Approach |
| 30233P1 | ICD-10-PCS | Transfusion of Nonautologous Frozen Red Cells into Peripheral Vein, Percutaneous Approach |
| 30277H1 | ICD-10-PCS | Transfusion of Nonautologous Whole Blood into Products of Conception, Circulatory, Via Natural or Artificial Opening |
| 30243P1 | ICD-10-PCS | Transfusion of Nonautologous Frozen Red Cells into Central Vein, Percutaneous Approach |
| 30277N1 | ICD-10-PCS | Transfusion of Nonautologous Red Blood Cells into Products of Conception, Circulatory, Via Natural or Artificial Opening |
| 30277P1 | ICD-10-PCS | Transfusion of Nonautologous Frozen Red Cells into Products of Conception, Circulatory, Via Natural or Artificial Opening |
| 99.00 | ICD-9-CM | Perioperative autologous transfusion of whole blood or blood components |
| 99.01 | ICD-9-CM | Exchange transfusion |
| 99.02 | ICD-9-CM | Transfusion of previously collected autologous blood |
| 99.03 | ICD-9-CM | Other transfusion of whole blood |
| 99.04 | ICD-9-CM | Transfusion of packed cells |

**Supplementary Table S2. Chart Abstraction Adjudication Definitions for ISTH Major and CRNM Bleeding**

| **Bleed Type** | **Definition** |
| --- | --- |
| ISTH Major Bleeding | Based on ISTH major bleeding definition,^4^ ISTH major bleeding is defined as bleeding with a symptomatic presentation and:   1. fatal bleeding, and/or 2. bleeding in a critical area or organ (i.e. intracranial, intraspinal, intraocular, retroperitoneal, intra-articular or pericardial, or intramuscular with compartment syndrome), and/or 3. clinically overt bleeding resulting in a fall in hemoglobin levels by at least 2g/dL (1.24 mmol/L) leading to transfusion of ≥ 2 units of whole blood or red blood cells.   To match the criteria set from the electronic health record (EHR)-based algorithms, the chart reviewer assessed whether (1) a drop of ≥ 2g/dL (1.24 mmol/L) in hemoglobin levels occurred within 48 hours of admission when compared with the most recent hemoglobin lab up to 6 months prior to admission, (2) a drop of ≥ 2g/dL (1.24 mmol/L) in hemoglobin levels occurred within 48 hours of another hemoglobin lab value during admission when ≥ 2 values were available, or (3) ≥ 2 units of blood transfusion occurred within 48 hours during admission, for inpatient encounters (or within 2 days of encounter date for outpatient encounters).^14^  We classified fatal bleed as death that occurred within 45 days of a bleed related encounter per chart review, matching the EHR-based algorithm for fatal bleed.  Bleeding in the eye that led to impaired vision was classified as intraocular bleeding. |
| CRNM Bleeding | Based on ISTH CRNM bleeding definition,^5^ CRNM bleeding was defined as any sign or symptom of hemorrhage that does not meet the ISTH major bleeding definition but meets one of the following criteria:   1. requiring medical intervention by a healthcare professional, 2. leading to hospitalization or increased level of care, or 3. prompts a face-to-face visit for bleeding evaluation   We also included a change in antithrombotic therapy upon discharge for bleeding, an interruption/cessation, or a dose reduction of an antithrombotic therapy. |

ISTH = International Society on Thrombosis and Haemostasis; CRNM = clinically relevant non-major

**Supplementary Table S3. Performance of Bleed Categories within ISTH Major Bleeding per EHR-based Algorithms**

| **ISTH Major Bleeding Categories** | **Sensitivity**  **Estimate (95% CI)** | **Specificity**  **Estimate (95% CI)** | **PPV**  **Estimate (95% CI)** | **NPV**  **Estimate (95% CI)** |
| --- | --- | --- | --- | --- |
| ISTH major bleeding | 0.91 (0.86, 0.94) | 0.92 (0.88, 0.95) | 0.91 (0.87, 0.94) | 0.92 (0.89, 0.95) |
| (a) Fatal bleeding | 0.91 (0.78, 0.97) | 0.97 (0.95, 0.99) | 0.80 (0.70, 0.90) | 0.99 (0.98, 1.00) |
| (b) Critical organ bleeding | 0.86 (0.73, 0.95) | 0.97 (0.95, 0.98) | 0.76 (0.66, 0.86) | 0.98 (0.97, 1.00) |
| (c) Symptomatic bleeding | 0.85 (0.77, 0.91) | 0.98 (0.96, 0.99) | 0.95 (0.91, 0.99) | 0.95 (0.93, 0.97) |

ISTH = International Society on Thrombosis and Haemostasis; PPV = positive predictive value; NPV = negative predictive value

**Supplementary Table S4. Performance of ISTH Major Bleeding- Sensitivity Analyses**

| **ISTH Major Bleeding** | **Sensitivity**  **Estimate (95% CI)** | **Specificity**  **Estimate (95% CI)** | **PPV**  **Estimate (95% CI)** | **NPV**  **Estimate (95% CI)** |
| --- | --- | --- | --- | --- |
| **Primary analysis** | 0.91 (0.86, 0.94) | 0.92 (0.88, 0.95) | 0.91 (0.87, 0.94) | 0.92 (0.89, 0.95) |
| **Sensitivity analysis** |  |  |  |  |
| (5) Critical organ bleeding codes in the inpatient setting, *primary position only* (rather than allowing all positions) | 0.91 (0.86, 0.94) | 0.92 (0.88, 0.95) | 0.91 (0.87, 0.94) | 0.92 (0.89, 0.95) |
| (6) Critical organ bleeding codes in *any* setting, *any* position (rather than inpatient setting only) | 0.91 (0.86, 0.94) | 0.90 (0.85, 0.93) | 0.88 (0.84, 0.92) | 0.92 (0.88, 0.95) |
| (7) *Death occurring during encounter* (rather than within a 45-day window post encounter) | 0.91 (0.86, 0.94) | 0.92 (0.88, 0.95) | 0.91 (0.87, 0.94) | 0.92 (0.89, 0.95) |
| (a) Fatal bleeding | 0.82 (0.60, 0.95) | 0.92 (0.89, 0.95) | 0.36 (0.27, 0.45) | 0.99 (0.98, 1.00) |
| (b) Critical organ bleeding | 0.58 (0.45, 0.70) | 0.97 (0.94, 0.98) | 0.76 (0.65, 0.87) | 0.93 (0.91, 0.95) |
| (c) Symptomatic bleeding | 0.85 (0.77, 0.91) | 0.98 (0.96, 0.99) | 0.95 (0.91, 0.99) | 0.95 (0.93, 0.97) |

ISTH = International Society on Thrombosis and Haemostasis; PPV = positive predictive value; NPV = negative predictive value. CI = confidence interval;

^±^ Bleed verification per EHR-based algorithms is defined as a (1) drop of ≥ 2g/dL (1.24 mmol/L) in hemoglobin levels within 48 hours of encounter compared to closest value in the 6 months prior, (2) drop of ≥ 2g/dL (1.24 mmol/L) in hemoglobin levels within 48 hours during an encounter; or (3) blood transfusion codes within 48 hours during inpatient encounter or within 48 hours of outpatient encounter.

**Supplementary Table S5. Performance of CRNM Bleeding- Sensitivity Analyses**

| **CRNM Bleeding** | **Sensitivity**  **Estimate (95% CI)** | **Specificity**  **Estimate (95% CI)** | **PPV**  **Estimate (95% CI)** | **NPV**  **Estimate (95% CI)** |
| --- | --- | --- | --- | --- |
| **Primary analysis** | 0.66 (0.54, 0.76) | 0.86 (0.82, 0.90) | 0.52 (0.44, 0.60) | 0.92 (0.90, 0.94) |
| **Sensitivity analysis** |  |  |  |  |
| (5) Anemia-related possible bleeding codes, in the secondary position as no-bleed | 0.66 (0.54, 0.76) | 0.90 (0.87, 0.93) | 0.60 (0.52, 0.69) | 0.92 (0.90, 0.94) |
| (6) Overt bleeding codes in the inpatient setting, *any position* (rather than primary position) with bleed verification as ISTH Major bleeding | 0.66 (0.54, 0.76) | 0.89 (0.86, 0.92) | 0.58 (0.49, 0.66) | 0.92 (0.90, 0.94) |
| (7) Overt bleeding codes in the inpatient setting, any position with *or without* bleed verification (rather than requiring bleed verification) as ISTH Major bleeding | 0.29 (0.19, 0.40) | 0.94 (0.90, 0.96) | 0.50 (0.37, 0.63) | 0.86 (0.84, 0.87) |

ISTH = International Society on Thrombosis and Haemostasis; CRNM = clinically relevant non-major; PPV = positive predictive value; NPV = negative predictive value. CI = confidence interval

^±^ Bleed verification per EHR-based algorithms is defined as a (1) drop of ≥ 2g/dL (1.24 mmol/L) in hemoglobin levels within 48 hours of encounter compared to closest value in the 6 months prior, (2) drop of ≥ 2g/dL (1.24 mmol/L) in hemoglobin levels within 48 hours during an encounter; or (3) blood transfusion codes within 48 hours during inpatient encounter or within 48 hours of outpatient encounter.
